# Mitochondrial deficiency impairs hypoxic induction of HIF-1 transcriptional activity and retards tumor growth

**DOI:** 10.18632/oncotarget.14415

**Published:** 2017-01-02

**Authors:** Masaru Koido, Naomi Haga, Aki Furuno, Satomi Tsukahara, Junko Sakurai, Yuri Tani, Shigeo Sato, Akihiro Tomida

**Affiliations:** ^1^ Cancer Chemotherapy Center, Japanese Foundation for Cancer Research, Tokyo 135-8550, Japan; ^2^ Department of Medical Genome Sciences, Graduate School of Frontier Sciences, The University of Tokyo, Minato-ku, Tokyo 108-8639, Japan

**Keywords:** HIF-1, HIF-1α, hypoxia, ρ^0^, mitochondria

## Abstract

Mitochondria can be involved in regulating cellular stress response to hypoxia and tumor growth, but little is known about that mechanistic relationship. Here, we show that mitochondrial deficiency severely retards tumor xenograft growth with impairing hypoxic induction of HIF-1 transcriptional activity. Using mtDNA-deficient ρ^0^ cells, we found that HIF-1 pathway activation was comparable in slow-growing ρ^0^ xenografts and rapid-growing parental xenografts. Interestingly, we found that *ex vivo* ρ^0^ cells derived from ρ^0^ xenografts exhibited slightly increased HIF-1α expression and modest HIF-1 pathway activation regardless of oxygen concentration. Surprisingly, ρ^0^ cells, as well as parental cells treated with oxidative phosphorylation inhibitors, were unable to boost HIF-1 transcriptional activity during hypoxia, although HIF-1α protein levels were ordinarily increased in these cells under hypoxic conditions. These findings indicate that mitochondrial deficiency causes loss of hypoxia-induced HIF-1 transcriptional activity and thereby might lead to a constitutive HIF-1 pathway activation as a cellular adaptation mechanism in tumor microenvironment.

## INTRODUCTION

Hypoxia is a major microenvironmental stress that results from vascular insufficiency in solid tumors. To survive and to proliferate in the tumor microenvironment, cancer cells must therefore adapt to hypoxic stress [1, 2]. A key regulator of hypoxic adaptation in cancer cells is hypoxia-inducible factor (HIF), a transcription factor that activates glycolysis and stimulates remodeling of the extracellular tumor microenvironment, thereby promoting tumor survival, progression and malignancy [3]. HIF comprises HIF-α and HIF-β. Three mammalian α subunit isoforms are known, of which HIF-1α and EPAS1/HIF-2α are known to be related to tumor proliferation and progression [4]. HIF-β is constitutively expressed, whereas HIF-α is expressed in an oxygen concentration-dependent manner [3]. Under normoxia, HIF-α is hydroxylated by prolyl hydroxylase, ubiquitinated through interaction with von Hippel-Lindau protein (pVHL) and degraded by proteasomes [3–6]. Under hypoxia, HIF-α protein is stabilized and heterodimerizes with constitutively expressed HIF-β protein. This heterodimer complex subsequently translocates into the nucleus and binds to a DNA hypoxia response element (HRE), thereby inducing expression of hundreds of genes required for hypoxia adaptation [3, 7].

Mitochondria generate ATP via oxidative phosphorylation (OXPHOS), and produce several precursors for macromolecule synthesis [8]. Mitochondria also activate various cellular stress signaling pathways, such as redox signaling [8], unfolded protein response [9], and hypoxic response [10–14]. In keeping with this wide involvement in cellular function, mitochondria can accelerate tumor growth, as shown in studies using mitochondrial DNA (mtDNA)-deficient ρ^0^ cells in mouse xenograft models [15, 16]. In clinical studies, single nucleotide variants in mtDNA have been observed to be associated with cancer prognosis [17, 18]. However, reduced or absent mitochondrial function, resulting from mtDNA mutations or low copy number, or reduced mitochondrial content, have been observed in many cancers types, including thyroid, pancreatic, kidney and colon cancer [19–25]. This suggests that there are some adaptive mechanisms during tumor development in which mitochondrial activity is decreased.

In this study, we investigated how deficiency of mitochondrial function affects tumor phenotype in xenograft models. We transplanted HT-29 human colon cancer (HT-29 Pt) cells and mtDNA-deficient HT-29 ρ^0^ (ρ^0^n) cells into immunodeficient mice, and found that tumor growth rates differed significantly between the two cell types. However, hypoxia-responsive genes were similarly expressed in both xenograft types, despite the fact that mitochondria can contribute to hypoxia response activation [10–14]. To resolve this apparent discrepancy, we investigated adaptive mechanisms of HT-29 ρ^0^ xenografts by establishing xenograft-derived cells *ex vivo*. Finally, using ρ^0^ and its derivative cells, we found that mtDNA deficiency resulted in impaired HIF-1 transactivation during hypoxia.

## RESULTS

### Hypoxic response activation in xenografts with or without mtDNA

**Figure 1:**
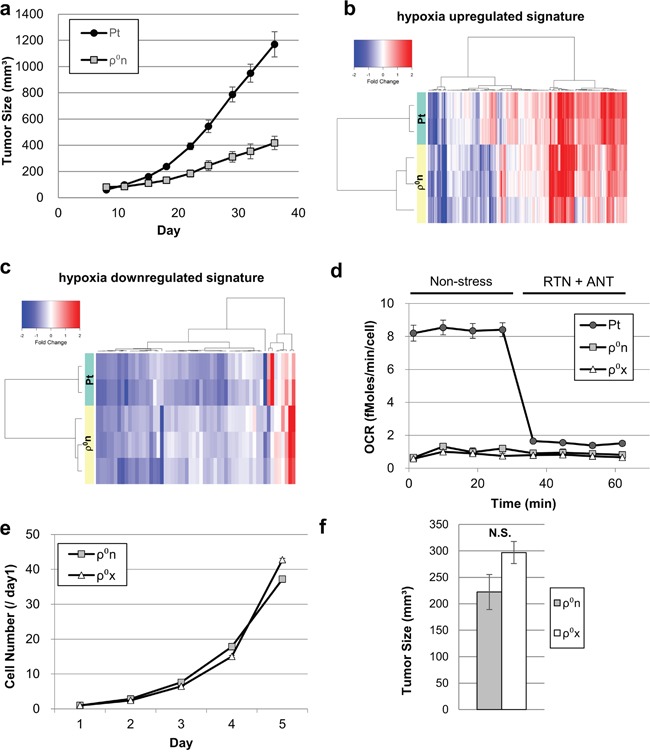
Hypoxic response activation in xenografted cells with or without mtDNA **a**. Growth of HT-29 ρ^0^n cell tumors (1 × 10^7^ cells/mouse) and HT-29 Pt cell tumors (1 × 10^6^ cells/mouse). **b, c**. Signal intensity ratios of gene expression. HT-29 Pt and ρ^0^n cells in xenograft were compared with those in culture. Comparisons were conducted using hypoxia-upregulated signatures in (b) and hypoxia-downregulated signatures in (c) (see gene lists in Supplementary Table 1 and 2). **d**. OCR in HT-29 Pt, ρ^0^n and ρ^0^x cells. After basal OCR was measured four times, rotenone (RTN, 0.5 μM) and antimycin A (ANT, 0.5 μg/ml) were added. **e**. *In vitro* growth ratio (/Day1) of HT-29 ρ^0^n cells and ρ^0^x cells. **f**. Tumor growth assessment comparing HT-29 ρ^0^n and ρ^0^x cells (1 × 10^7^ cells/mouse). Tumor volume was evaluated on day 24.

We first engrafted HT-29 Pt cells and HT-29 ρ^0^n cells into nude mice. Growth of HT-29 ρ^0^n cell tumors was markedly slower than that of HT-29 Pt cell tumors (Figure 1a). Gene expression profiling revealed that changes in gene expression patterns between cultured cells and xenografts were highly similar for both HT-29 ρ^0^n and HT-29 Pt cells. (Supplementary Figure 1a and 1b). Additionally, gene set enrichment analysis (GSEA) showed that hypoxia-responsive genes were significantly activated in both xenograft types (Supplementary Figure 1c). Hierarchical clustering of hypoxia-responsive genes indicated comparable activation of hypoxic gene expression in xenografts of HT-29 ρ^0^n and Pt cells (Figure 1b and 1c).

### Establishment of a cell line from xenografts of HT-29 ρ^0^ cells

Next, we cultured HT-29 ρ^0^n cells isolated from the murine xenografts *ex vivo*, and then established HT-29 ρ^0^x cells. The HT-29 ρ^0^x cells had no human or murine mtDNA (Supplementary Figure 2) and did not exhibit mitochondria-dependent oxygen consumption (Figure 1d). HT-29 ρ^0^x cells had almost the same proliferation rate in culture as the original HT-29 ρ^0^n cells (Figure 1e), and proliferated in nude mice with a slightly, but not significantly, increased rate (*p* = 0.1149) (Figure 1f).

**Figure 2:**
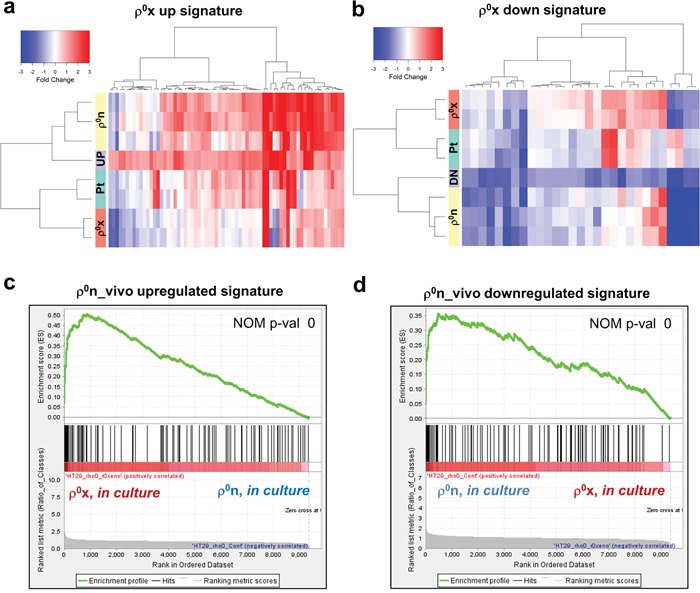
Cultured HT-29 ρ0x cells retained some characteristics of xenografted HT-29 ρ0n cells **a, b**. Signal intensity ratio of gene expression. HT-29 Pt, ρ^0^n and ρ^0^x cells in xenograft were compared with those in culture, using our defined ρ^0^x upregulated signature in (a), and ρ^0^x downregulated signature in (b) (see gene lists in Supplementary Table 3 and 4). Signal intensity ratio of gene expression in HT-29 ρ^0^x cells in culture, compared with HT-29 ρ^0^n cells in culture, are represented as “UP” or “DN”. **c, d**. Enrichment plots of ρ^0^n_vivo upregulated and downregulated signatures, for genes expressed highly in HT-29 ρ^0^x cells and ρ^0^n cells (see gene lists in Supplementary Table 5 and 6). GSEA indicated a nominal *P*-value of 0 (statistically significant enrichment).

Comparative microarray analysis of HT-29 ρ^0^x cells and the original ρ^0^n cells showed distinct differences in gene expression patterns (Figure 2a and 2b). We first compared HT-29 ρ^0^x cells with HT-29 ρ^0^n cells in culture, and identified 69 constitutively upregulated probe sets and 29 downregulated probe sets in the HT-29 ρ^0^x cells (Supplementary Table 3-4). The ρ^0^x signature of these gene sets showed that the gene expression patterns in HT-29 ρ^0^x cells in culture were similar to those in HT-29 ρ^0^n cells in xenograft (Figure 2a and 2b). Next, we compared HT-29 ρ^0^n cells in xenograft with those in culture and identified 213 upregulated probe sets and 161 downregulated probe sets in the xenograft cells, which we named ρ^0^n_vivo upregulated or downregulated signature gene sets, respectively (Supplementary Table 5 and 6). The GSEA algorithm revealed that these ρ^0^n_vivo upregulated and downregulated genes were significantly upregulated and downregulated in HT-29 ρ^0^x cells, respectively, compared with in HT-29 ρ^0^n cells (Figure 2c and 2d). Thus, cultured HT-29 ρ^0^x cells retained a similar expression phenotype to that of ρ^0^ xenograft cells, which was likely acquired during xenograft growth.

**Figure 3:**
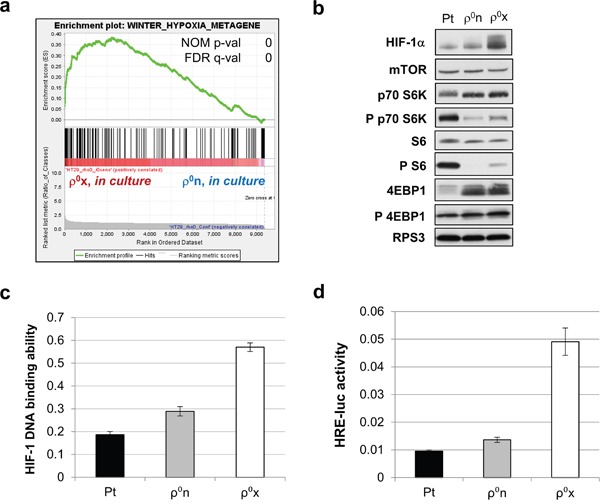
Constitutive activation of hypoxic response in HT-29 ρ0x cells **a**. Enrichment plot of “winter hypoxia metagene” [59] from MSigDB c2 curated collections, for genes expressed highly in HT-29 ρ^0^x cells and ρ^0^n cells (both in culture). **b**. Immunoblot analysis of HIF-1α, and phosphorylation status of proteins involved in mTOR signaling in HT-29 Pt, ρ^0^n and ρ^0^x cells under normoxia. RPS3 was used as a loading control. **c, d**. HIF-1 DNA-binding ability (c) and HRE-luc activity (d) in HT-29 Pt, ρ^0^n and ρ^0^x cells under normoxia.

GSEA of HT-29 ρ^0^x and the original ρ^0^n cells, both in culture, showed that several hypoxia-related signature gene sets were highly enriched in HT-29 ρ^0^x cells. (Figure 3a, Supplementary Table 7, 8 and Supplementary Figure 3). Furthermore, HIF-1α protein was constitutively expressed at higher levels in HT-29 ρ^0^x than in ρ^0^n cells (Figure 3b). Consistent with these findings, for the same amount of nuclear protein, HIF-1 DNA binding activity was twofold higher in HT-29 ρ^0^x cells than in HT-29 ρ^0^n cells (Figure 3c). HIF-1 transcriptional activity was also five times higher in HT-29 ρ^0^x cells than in HT-29 ρ^0^n cells, as determined by HIF-1 reporter assay (Figure 3d). These data suggest that HT-29 ρ^0^x cells show a constitutively activated hypoxic response with increased HIF-1α protein, even in normoxia.

Next, we evaluated the sequence of the gene encoding pVHL, *VHL*, and found that there was no mutation in this gene in HT-29 ρ^0^x, Pt or ρ^0^n cells (data not shown). We examined the phosphorylation status of mechanistic target of rapamycin (mTOR) signaling proteins, including ribosomal protein S6 (S6), 70 kDa ribosomal protein S6 kinase 1 (p70 S6K), and eukaryotic translation initiation factor 4E-binding protein 1 (4EBP1), which are involved in regulating HIF-1α translation [26, 27]. 4EBP1 phosphorylation was comparable in HT-29 ρ^0^x and HT-29 ρ^0^n cells; however, levels of phosphorylated p70 S6K and S6 were slightly higher in HT-29 ρ^0^x cells than in HT-29 ρ^0^n cells (Figure 3b). Notably, HT-29 Pt cells exhibited much higher levels of phosphorylated p70 S6K and S6 than HT-29 ρ^0^n or ρ^0^x cells (Figure 3b). Thus, mTOR signaling pathway activity was not consistent with constitutively expressed HIF-1α protein levels, and appeared to be differently regulated between cells with and without mtDNA.

### Mitochondria-dependent HIF-1 transcriptional activity under hypoxia

**Figure 4:**
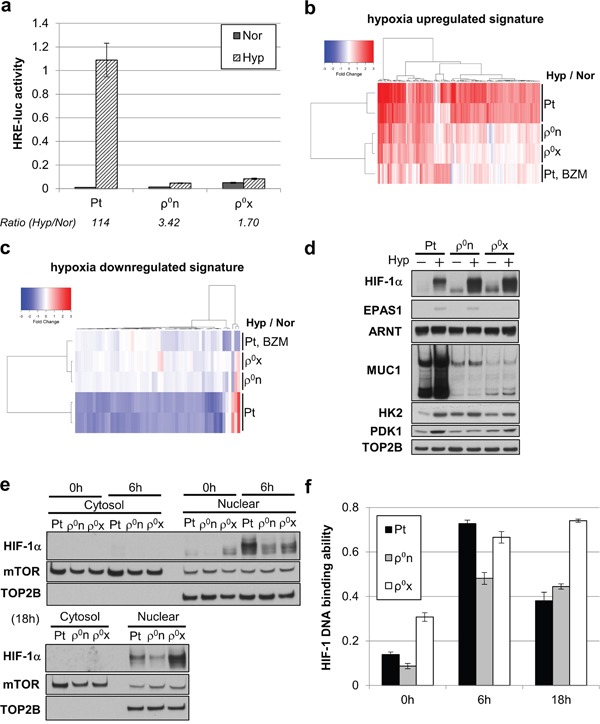
Mitochondria-enhanced HIF-1 transcriptional activity after HIF-1α stabilizing and translocating into the nucleus **a**. HRE-luc activity of HT-29 Pt, ρ^0^n and ρ^0^x cells after 18 hours of hypoxia (Hyp) and normoxia (Nor). **b, c**. Signal intensity ratio of gene expression, comparing HT-29 Pt, ρ^0^n, ρ^0^x and bortezomib (BZM)-treated Pt cells under hypoxia. Hypoxia-upregulated genes were used in (b) and hypoxia-downregulated genes were used in (c) (see gene lists in Supplementary Table 1 and 2). **d**. Immunoblot analysis of HIF-1α, EPAS1/HIF-2α, ARNT/HIF-1β and HIF-1-driven gene products (MUC1, HK2 and PDK1) after 18 hours of hypoxia. DNA topoisomerase IIβ (TOP2B) was used as a loading control. **e-f**. Immunoblot analysis of HIF-1α (e) and HIF-1 DNA-binding ability (f) in the same nuclear extracts of HT-29 Pt, ρ^0^n and ρ^0^x cells, at the indicated hypoxia durations. mTOR was used as a loading control, and TOP2B was used as a nuclear extract indicator.

Using a HIF-1 reporter assay system, we examined HIF-1 transcriptional activity during hypoxia. Hypoxia enhanced HIF-1 transcriptional activity by more than 100 times in HT-29 Pt cells, but at most 2–3 times in HT-29 ρ^0^n and ρ^0^x cells (Figure 4a). Consistent with this, in hypoxic conditions HT-29 Pt cells exhibited activated expression of many hypoxia-responsive genes, whereas both HT-29 ρ^0^n and ρ^0^x cells showed activation of only a small number of those genes (Figure 4b and 4c). Similarly, proteins encoded by HIF-1α–regulated genes, including hexokinase 2 (HK2), pyruvate dehydrogenase kinase (PDK1) [3] and mucin 1 (MUC1) [28], were clearly increased in hypoxic HT-29 Pt cells, whereas these proteins were hardly increased in HT-29 ρ^0^n or ρ^0^x cells under hypoxia (Figure 4d). These data indicate that HT-29 ρ^0^n and ρ^0^x cells show only marginal activation of HIF-1-induced gene expression under hypoxia.

Interestingly, HIF-1α stabilization in HT-29 ρ^0^n and ρ^0^x cells was comparable with that in HT-29 Pt cells under hypoxic conditions (Figure 4d). Meanwhile, levels of EPAS1/HIF-2α protein, another isoform of HIF-α, did not increase in HT-29 ρ^0^x cells, but did increase in HT-29 ρ^0^n and HT-29 Pt cells under hypoxia (Figure 4d). Nuclear accumulation of stabilized HIF-1α and its DNA binding activity were generally consistent in cellular nuclear extracts of HT-29 Pt, ρ^0^n, and ρ^0^x cells, after both 6 and 18 hours of hypoxia (Figure 4e and 4f). These results indicate that HIF-1 transcriptional activity was enhanced via mitochondrial activity after stabilization and nuclear translocation of HIF-1α protein occurred during hypoxia.

Phenomena similar to those observed in HT-29 ρ^0^n and ρ^0^x cells in our study have previously been seen in cells treated with bortezomib (BZM), a proteasome inhibitor [29–31]. BZM has been shown to stabilize HIF-1α proteins, even in normoxia, but to suppress HIF-1 transcriptional activity under hypoxia [29–31]. We found that BZM-treated HT-29 Pt cells exhibited changed expression levels of a broad range of genes, including some hypoxia-responsive genes (Supplementary Figure 4). Interestingly, the patterns of hypoxia-induced gene expression changes in BZM-treated HT-29 Pt cells were similar to those in HT-29 ρ^0^n and ρ^0^x cells (Figure 4b and 4c), suggesting that the mechanisms underlying HIF-1 transcriptional activity inhibition overlapped in mitochondria-deficient cells and BZM-treated cells.

### Inhibition of mitochondrial activity suppresses HIF-1 transcriptional activity

**Figure 5:**
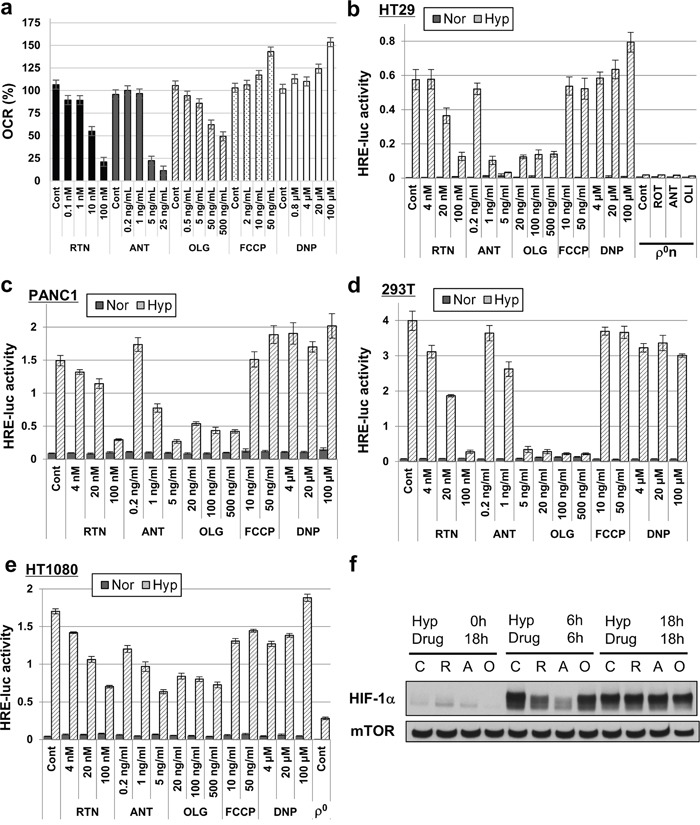
HIF-1 transcriptional activity suppression by mitochondrial inhibitors **a**. OCR of HT-29 Pt cells treated with the indicated concentrations of rotenone (RTN), antimycin A (ANT), oligomycin (OLG), FCCP or DNP. **b-e**. HRE-luc activity of indicated cells (HT-29, PANC1, 293T and HT1080) after 18 hours of hypoxia (Hyp) or normoxia (Nor), after treating with the indicated concentrations of RTN, ANT, OLG, FCCP or DNP. **f**. Immunoblot analysis of HIF-1α. HT-29 Pt cells were treated at the indicated times and under hypoxic stress (Hyp) with no inhibitor (control, C) rotenone (R, 100 nM), antimycin A (A, 5 ng/ml), or oligomycin (O, 0.5 μg/ml). mTOR was used as a loading control.

We examined the effects of mitochondria on hypoxic induction of HIF-1 transcriptional activity by pharmacologically inhibiting OXPHOS in human pancreatic cancer PANC1, human sarcoma HT1080 and human embryonic kidney 293T cells as well as in HT-29 Pt cells. Rotenone (a complex 1 inhibitor), antimycin A (a complex 3 inhibitor), and oligomycin (a complex 5 inhibitor) suppressed hypoxic induction of HIF-1 transcriptional activity at the same concentration range as reduced oxygen consumption rate (OCR) (Figure 5a–5e and Supplementary Figure 5). The compounds did not decrease intracellular ATP levels (Supplementary Figure 6) and did not inhibit HIF-1 transcriptional activity in normoxia (Supplementary Figure 7). Consistent with the results using HT-29 ρ^0^n and ρ^0^x cells (Figure 4d), pharmacological inhibition of OXPHOS in HT-29 Pt cells did not inhibit stabilization of HIF-1α after 18 hours under hypoxia (Figure 5f). After 6 hours under hypoxia, HIF-1α protein stabilization was not suppressed by oligomycin, although we saw slight stabilization inhibition with both rotenone and antimycin A (Figure 5f). It is important to note that HT1080 ρ^0^ cells also did not show activation of HIF-1 transcriptional activity under hypoxia (Figure 5e). In contrast to the OXPHOS inhibitors, chemical uncouplers of OXPHOS, trifluoromethoxy carbonyl cyanide phenylhydrazone (FCCP) and 2, 4-dinitrophenol (DNP), somewhat increased oxygen consumption (Figure 5a) and did not inhibit hypoxia-activated HIF-1 transcriptional activity in PANC1, HT1080, 293T or HT-29 Pt cells (Figure 5b–5e).

**Figure 6:**
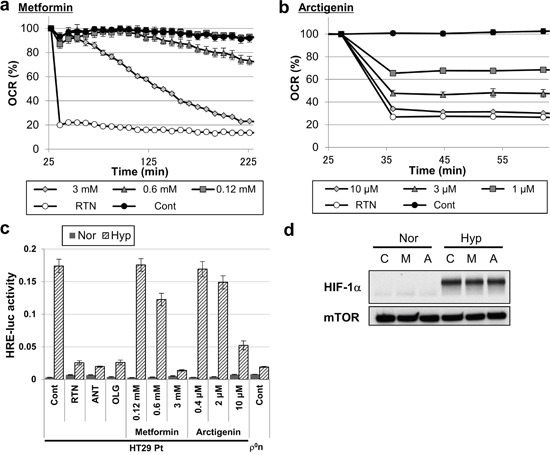
Inhibition of mitochondrial respiration and hypoxic responses by treatment with antitumor compounds **a, b**. OCR of HT-29 Pt cells treated with the indicated concentrations of metformin (a) or arctigenin (b). Rotenone (RTN, 200 nM) was used as a positive control of OCR inhibition. **c**. HRE-luc activity of HT-29 Pt cells after 18 hours of hypoxia (Hyp) or normoxia (Nor), treated with the indicated concentrations of metformin or arctigenin. Cells treated with rotenone (RTN, 100 nM), antimycin A (ANT, 5 ng/ml) or oligomycin (OLG, 5 μg/ml); HT-29 ρ^0^n cells were used as positive controls. **d**. Immunoblot analysis of HIF-1α in HT-29 Pt cells treated with metformin (M, 3mM) or arctigenin (A, 10 μM) after 18 hours of hypoxia (Hyp) or normoxia (Nor). mTOR was used as a loading control.

Finally, we used metformin [32] and arctigenin (registered on the UMIN clinical trial registry, UMIN000010111), which are promising antitumor compounds used in clinical trials, both of which inhibit OXPHOS by inhibiting mitochondrial complex 1 [33, 34]. Metformin and arctigenin reduced the OCR of HT-29 Pt cells (Figure 6a and 6b) and inhibited induction of HIF-1 transcriptional activity under hypoxia (Figure 6c), without preventing HIF-1α stabilization (Figure 6d). Collectively, these results demonstrate that hypoxic induction of HIF-1 transcriptional activity can be suppressed by pharmacological inhibition of OXPHOS in mitochondria-containing cancer cells.

## DISCUSSION

We have shown that mitochondrial function is critical for rapid tumor growth and for HIF-1 transcriptional activity. HIF-1 pathway activation is a general adaptive mechanism during development of solid tumors with particular microenvironment [1, 2], including HT-29 xenografts [35, 36]. Consistent with these previous studies, we found that HT-29 Pt and ρ^0^n cells in xenografts exhibited similar activation of hypoxia-responsive gene expression, despite the fact that ρ^0^n cells in culture only showed minimal HIF-1 transcriptional activity under hypoxia as well as normoxia. Instead, we found that HT-29 ρ^0^x cells, established from xenografts of HT-29 ρ^0^n cells, exhibited a modest increase in constitutive HIF-1α expression and HIF-1 pathway activation. These findings suggested the importance of the HIF-1 pathway for cellular adaptation to xenograft microenvironment. However, both ρ^0^n and ρ^0^x cells, which are both mtDNA-deficient, showed loss of HIF-1 transactivation activity during hypoxia. Such a loss of HIF-1 transactivation could lead to the failure of proper response to the dynamically fluctuating oxygen tension that occurs in proliferating tumors [1, 2]. Thus, it is conceivable that difference in capacity for cellular HIF-1 transcriptional activity, which depends on mitochondrial function, could be a determinant of adaptability in xenograft microenvironment. Further studies will be needed to establish a link between the capacity of HIF-1 transcriptional activity and tumor growth rate.

In agreement with our present study, a previous study showed that mtDNA depletion retarded growth of human glioblastoma DBTRG-O5MG and human breast cancer MCF-7 cell tumors in mouse xenograft models [15]. A recent study using murine cancer cells showed that although mtDNA depletion delayed tumor growth, engrafted murine ρ^0^ cells acquired mtDNA from host cells during tumor growth, and these mtDNA-recovered cells exhibited faster tumor growth than the original ρ^0^ cells [16]. Our findings together with these published data reinforce the importance of mitochondria for rapid tumor growth.

It is likely that constitutive HIF-1 activation in HT-29 ρ^0^x cells may be an adaptive mechanism acquired during tumor growth in mice, possibly to compensate for loss of mitochondria-dependent induction of HIF-1 transcriptional activity during hypoxia. It should be noted that in clinical settings, clear cell renal cell carcinoma (ccRCC) is known to exhibit significant loss of mitochondrial proteins and often shows somatic VHL mutation, resulting in constitutive HIF-α subunit stabilization [37, 38]. Thus, HIF activation and mitochondrial dysfunction can occur simultaneously in clinical settings and the findings of the following ccRCC studies may be useful for interpreting our study. In ccRCC, development of xenografts with mutated pVHL has been shown to require HIF-α activity [39, 40], and HIF-1 pathway upregulation suppressed biosynthesis of mitochondria [41, 42]. These observations indicate that the constitutively activated HIF-1 pathway can maintain tumor tissues, despite lowered mitochondrial activity. By analogy, it is conceivable that constitutive HIF-1 activation is involved in the tissue maintenance in HT-29 ρ^0^ cell xenografts.

It is known that the relationship between HIF-1α stabilization and mitochondria varies with oxygen concentration. Under mild hypoxia (1%–1.5% O_2_), mitochondrial dysfunction inhibits HIF-1α stabilization, possibly through loss of reactive oxidative species generated by mitochondria [10–14]. Conversely, in keeping with our present study, previous studies showed that HIF-1α stabilization normally occurred in severe hypoxia (0–0.1% O_2_), even after mtDNA depletion or rotenone treatment [10, 43]. As demonstrated in this study, mitochondria are also required for hypoxic induction of HIF-1 transcriptional activity after sufficient amounts of hypoxia-stabilized HIF-1α protein is produced. Taken together, these findings indicate that mitochondria can stimulate activation of the HIF-1 pathway at multiple steps, from HIF-1α stabilization to HIF-1 transcriptional activity, depending on the severity of hypoxia.

Gene expression analysis showed that HIF-1 transcriptional activity may be inhibited by mechanisms that overlap between mitochondria-deficient and proteasome-inhibited cells. Recently, it has been reported that HIF-1 has several co-activators, including pyruvate kinase isozymes M2 [44], RuvB-like AAA ATPase 1 [45] and lysine-specific demethylase 4C [46]. Moreover, one study identified 190 HIF-1α-binding proteins [46]. Thus, as-yet-undetermined mechanisms may be involved in regulating HIF-1 transcriptional activity. Considering the mechanism of action of BZM, it is likely that some proteasome-degraded proteins may be negative regulators of HIF-1 transcriptional activity. Further study will be needed to clarify the overlapping mechanisms between mitochondria- and proteasome-dependent enhancements in HIF-1 transcriptional activity during hypoxia.

Metformin and arctigenin are potential anticancer drugs [34, 47] with unfolded protein response-inhibiting activity via OXPHOS inhibition [9, 48, 49]. These compounds also inhibited hypoxic induction of HIF-1 transcriptional activity in HT-29 Pt cells in our study. In addition to these compounds, several compounds that inhibit both mitochondria and the HIF-1 pathway are currently being developed. For example, one study screened 691,200 compounds for HIF-1 pathway inhibitors, and identified alkyliminophenylacetate inhibitors, which inhibited mitochondrial reactive oxygen species generation under hypoxia [50]. Other studies reported that a potent anticancer drug, BAY 87-2243, inhibited HIF-1α stabilization and mitochondrial complex 1 under hypoxia [51, 52]. Taken together, our findings indicate that the mitochondria-regulated mechanisms of HIF-1 transactivation during hypoxia may be good targets for novel cancer chemotherapy development.

## MATERIALS AND METHODS

### Cell cultures and treatments

We used human colorectal adenocarcinoma HT-29 [9], human pancreatic carcinoma PANC1 [9], human fibrosarcoma HT1080 [53] and human embryonic kidney 293T [54] cells. HT-29 ρ^0^n cells and HT1080 ρ^0^ cells were established as previously described [9], and establishment of HT-29 ρ^0^x cells is described below. HT-29, PANC1 and HT1080 cells were cultured in PRMI-1640 (Wako, Osaka, Japan), and 293T cells were cultured in DMEM (Wako). Each medium was supplemented with 10% heat-inactivated FBS and 100 μg/ml of kanamycin (growth medium). To culture HT-29 ρ^0^n cells, ρ^0^x cells and HT1080 ρ^0^ cells, 50 μg/mL uridine (Sigma, St. Louis, MO, USA) and 1mM sodium pyruvate (Sigma) were supplemented in growth medium (ρ^0^ growth medium). To compare HT-29 Pt cells and ρ^0^n or ρ^0^x cells under the same conditions, HT-29 Pt cells and HT1080 parental cells were also cultured in ρ^0^ growth medium after preculture. All cells used in this study were pretreated with MC-210 (DS Pharma Biomedical, Osaka, Japan).

### Isolation of HT-29 ρ^0^x cells

We first transplanted HT-29 ρ^0^n cells (1×10^7^ cells) into 6-week-old BALB/c-nu/nu mice (Charles River Japan, Kanagawa, Japan). After 148 days, we transplanted 2 mm^3^ of cells from the HT-29 ρ^0^n–derived tumor directly into new BALB/c-nu/nu mice. A third transplantation was conducted after another 35 days. After a further 47 days, we enzymatically digested mouse fibroblasts using dispase 1 (Wako) and collagenase (Sigma), and isolated HT-29 ρ^0^x cells.

### Chemicals

Rotenone (Calbiochem, Darmstadt, Germany), antimycin A (Santa Cruz Biotechnology, Santa Cruz, CA, USA), oligomycin (Calbiochem), FCCP (Sigma) and DNP (Nacalai, Kyoto, Japan) were dissolved in DMSO (Wako). Metformin (Sigma) was dissolved in sterilized distilled water. AnaeroPack-Kenki 5% (Mitsubishi Gas Chemical, Tokyo, Japan), an oxygen absorber, was used to create hypoxic conditions in a 2.5-L AnaeroPack-Rectangular Jar (Mitsubishi Gas Chemical). According to the manufacturer, this oxygen absorber reduces O_2_ levels in air to under 1% within 1 hour, and ultimately produces nearly anoxic conditions. It is possible that a small amount of dissolved oxygen remained in the medium in our hypoxic conditions.

### HRE reporter assay

Cells were transfected with firefly luciferase–containing 5× HRE reporter vectors (kindly provided by Dr Toru Shibata [55]) and *Renilla* luciferase-containing phRL-CMV (Promega, Fitchburg, WI, USA) using Lipofectamine 2000 (Invitrogen, Carlsbad, CA, USA). *Renilla* luciferase was used as an internal control, and activity of firefly luciferase relative to *Renilla* luciferase (mean ± SEM, N = 4) was determined using the Dual-Glo Luciferase Assay System (Promega), according to the manufacturer's standard protocols.

### HIF-1 DNA-binding assay

Cells were fractionated into cytoplasmic and nuclear material using a Nuclear Extract Kit (Active Motif, Carlsbad, CA, USA), and HIF-1 DNA-binding ability (mean ± SEM, N = 3) was measured and compared by using equal amounts of the nuclear compartment with TransAM HIF-1 (Active Motif), according to the manufacturer's standard protocols.

### Immunoblot analysis

Immunoblot analysis was conducted as described previously [48]. Briefly, equal amounts of proteins were resolved on an SDS-polyacrylamide gradient gel and transferred by electroblotting onto a nitrocellulose membrane. Membranes were probed with the indicated primary antibodies. The specific signals were visualized with a chemiluminescence detection system using appropriate secondary antibodies (Perkin-Elmer, Waltham, MA, USA). The following antibodies were used for immunoblotting: anti-TOP2B (Abcam, Cambridge, MA, USA), anti-HIF-1α, anti-ARNT/HIF-1β (BD Transduction, San Jose, CA, USA), anti-EPAS1/HIF-2α, anti-mTOR, anti-MUC1, anti-HK2, anti-PDK1, anti-4EBP1, anti-phospho-4EBP1 (Ser65), anti-S6, anti-phospho-S6 (Ser235/236), anti-S6K, anti-phospho-S6K (Thr389) and anti-RPS3 (Cell Signaling Technology, Danvers, MA, USA). TOP2B, mTOR and RPS3 were used as controls.

### RNA preparation

Total RNA from cultured cells was extracted using an RNeasy RNA purification kit (Qiagen, CA, USA). Xenografted cells stabilized in Allprotect Tissue Reagent (Qiagen) were fractured by TissueLyser (Qiagen), and their RNA was extracted in the same way. RNA quality was checked with a 2100 Bioanalyzer (Agilent Technologies, Santa Clara, CA, USA). For microarray analysis, we transplanted HT-29 Pt cells into nude mice and collected two samples of xenografts from different nude mice, 13 days and 37 days after transplantation. Similarly, we transplanted HT-29 ρ^0^n cells into nude mice and collected samples of xenografted HT-29 ρ^0^n cells. Two samples were collected, from different nude mice, 20 days and 148 days after transplantation. A third sample was collected as follows: HT-29 ρ^0^n cells were transplanted into a nude mouse; after 148 days, HT-29 ρ^0^n cells from this xenograft were transplanted into a different nude mouse; and the sample was collected after a further 35 days. We also transplanted HT-29 ρ^0^x cells into nude mice and collected two samples of xenografts from different nude mice, 13 days and 20 days after transplantation.

### Microarray analysis

Microarray analysis was conducted using GeneChip Human Genome U133 Plus 2.0 arrays (Affymetrix, Santa Clara, CA, USA) following standard protocols. Normalization was carried out using Frozen Robust Multiarray Analysis using the R package ‘frma’ (version 1.14.0) [56] with default parameters. We defined upregulated and downregulated probes by calculating the signal intensity (SI) ratio to each control sample as follows: if the SI of the denominator was ≥100 and the SI ratio was ≥2, then we defined the probe as upregulated; if the SI of the numerator was ≥100 and the SI ratio was ≤1/2, then we defined the probe as downregulated. If the SI of denominator was <100 and the SI of the numerator was ≥300, then we defined the probe as upregulated; if the SI of the numerator was <100 and the SI of the denominator was ≤300, then we defined the probe as downregulated. Based on these definitions, we defined five signature gene sets for each direction change of gene expression as follows: (1) hypoxia upregulated (downregulated) signature, upregulated (downregulated) in any one of HT-29 Pt cells, HT-29 ρ^0^n cells, HT-29 ρ^0^x cells and HT-29 Pt cells treated with BZM, (2) ρ^0^x upregulated (downregulated) signature, upregulated (downregulated) in HT-29 ρ^0^x cells compared with in HT-29 ρ^0^n cells, (3) ρ^0^n_vivo upregulated (downregulated) signature, upregulated (downregulated) in HT-29 ρ^0^n cells in xenograft compared with in these cells in culture, (4) BZM upregulated (downregulated) signature, upregulated (downregulated) in BZM-treated HT-29 Pt cells compared with in inhibitor-untreated HT-29 cells, (5) xenograft upregulated (downregulated) signature, upregulated (downregulated) in HT-29 Pt or HT-29 ρ^0^n cells in xenograft compared with in these cells in culture. Probe sets targeting mtDNA transcripts were selected based on annotations provided by the manufacturer. The lists and numbers of probe sets are provided in the Supplementary Tables 1-6 and 9-13. Unsupervised hierarchical clustering was performed using the Euclidean distance and Ward's linkage method. GSEA was performed with GSEA software (v2.0.14, Broad Institute) [57, 58] using the Molecular Signatures Database (MSigDB, v4.0) c2 curated gene [58] or our defined signature gene sets. For GSEA, we used probes with ≥100 SI to compare samples. The microarray data sets were deposited in the NCBI Gene Expression Omnibus under the series accession no. GSE80320.

### OCR measurement

OCR (mean ± SEM, N = 4 at each time point) was determined using an XF24 Extracellular Flux Analyzer (Seahorse Bioscience, North Billerica, MA, USA), following the manufacturer's standard protocols. To compare OCR between different cells, after the XF24 assay we counted the cell number per well using a CDA-500 particle counter (Sysmex, Hyogo, Japan). We evaluated OCR in non-stressed conditions at the fourth measurement time point.

### Real-time quantitative PCR

Purified RNA was examined by real-time quantitative PCR using an ABI Prism 700 (Applied Biosystems, Foster City, CA, USA), according to the manufacturer's standard protocols. Human or murine FAM-labeled D-LUX primer sets (Invitrogen) for *mitochondrially encoded NADH dehydrogenase 6* (*MTND6*) and *mitochondrially encoded cytochrome c oxidase II* (*MTCO2*) were designed by D-LUX Designer software (Invitrogen) as follows: *MTND6* (Forward), 5′-TTCACCCACAGCACCAATCCTA-3′; *MTND6* (Reverse), 5′-CGCTATGAGTGTTTTAGTGGGGTTAG[FAM]G-3′; *MTCO2* (Forward), 5′-CGTC CACAGATGCAATTCCCGGA[FAM]G-3′; *MTCO2* (Reverse), 5′-CGGTCGTGTAGCGGTGAAAG-3′; *mtnd6* (Forward), 5′-CGCTAAAGGAGGGATTGGGGTAG [FAM]G-3′; *mtnd6* (Reverse), 5′-AATACCCGCAAACA AAGATCACC-3′; *mtco2* (Forward), 5′-CGGTTCAAGCAACAGTAACATCAAAC[FAM]G-3′; *mtco2* (Reverse), 5′-GACAATGGGCATAAAGCTATGGTT-3′.

### Tumor growth assessment

Cells were implanted subcutaneously in the right flank region of 6-week-old BALB/cAJcl-nu/nu mice (Charles River). The experiments were started 8 days after the implantation. The length (L) and width (W) of the tumor were measured, and tumor volume (TV) was calculated as TV = (LWW)/2 (mean ± SEM, N = 7). We performed Welch's two-sample t-test on the data for HT-29 ρ^0^n and ρ^0^x cells using R (version 3.0.3).

### Intracellular ATP level assessment

Cells were seeded at 5 × 10^3^ cells/well in a 96-well plate and cultured for 24 h. The cells were then cultured for a further 18 h in hypoxic or normoxic conditions. ATP content was quantified using a CellTiter-Glo luminescent cell viability assay (Promega), according to the manufacturer's protocol. ATP levels were normalized by cell numbers at the measurement point.

## SUPPLEMENTARY MATERIALS FIGURES AND TABLES





## Abbreviations

4EBP1: eukaryotic translation initiation factor 4E-binding protein 1
ATP: adenosine triphosphate
BZM: bortezomib
ccRCC: clear cell renal cell carcinoma
DNP: 2, 4-dinitrophenol
FCCP: trifluoromethoxy carbonyl cyanide phenylhydrazone
GSEA: gene set enrichment analysis
HIF: hypoxia-inducible factor
HK2: hexokinase 2
HRE: hypoxia response element
mtDNA: mitochondrial DNA
mTOR: mechanistic target of rapamycin
MUC1: Mucin 1
OCR: oxygen consumption rate
OXPHOS: oxidative phosphorylation
p70: S6K 70 kDa ribosomal protein S6 kinase 1
PDK1: pyruvate dehydrogenase kinase
pVHL: von Hippel-Lindau protein
S6: ribosomal protein S6
