# Supplementary material for: Mitochondrial deficiency impairs hypoxic induction of HIF-1 transcriptional activity and retards tumor growth
Source: Oncotarget. 2017 Jan 2;8(7):11841–54. doi: 10.18632/oncotarget.14415 (PMC5355308; doi:10.18632/oncotarget.14415)
Supplement: Supplementary file 1 [file oncotarget-08-11841-s001.pdf]

Mitochondrial deficiency impairs hypoxic induction of HIF-1 transcriptional activity and retards tumor growth

SUPPLEMENTARY DATA

SUPPLEMENTARY REFERENCES

1. Elvidge GP, Glenny L, Appelhoff RJ, Ratcliffe PJ, Ragoussis J, Gleadle JM. Concordant regulation of gene expression by hypoxia and 2-oxoglutarate-dependent dioxygenase inhibition: the role of HIF-1alpha, HIF-2alpha, and other pathways. J Biol Chem. 2006; 281: 15215–26. doi: 10.1074/jbc.M511408200.

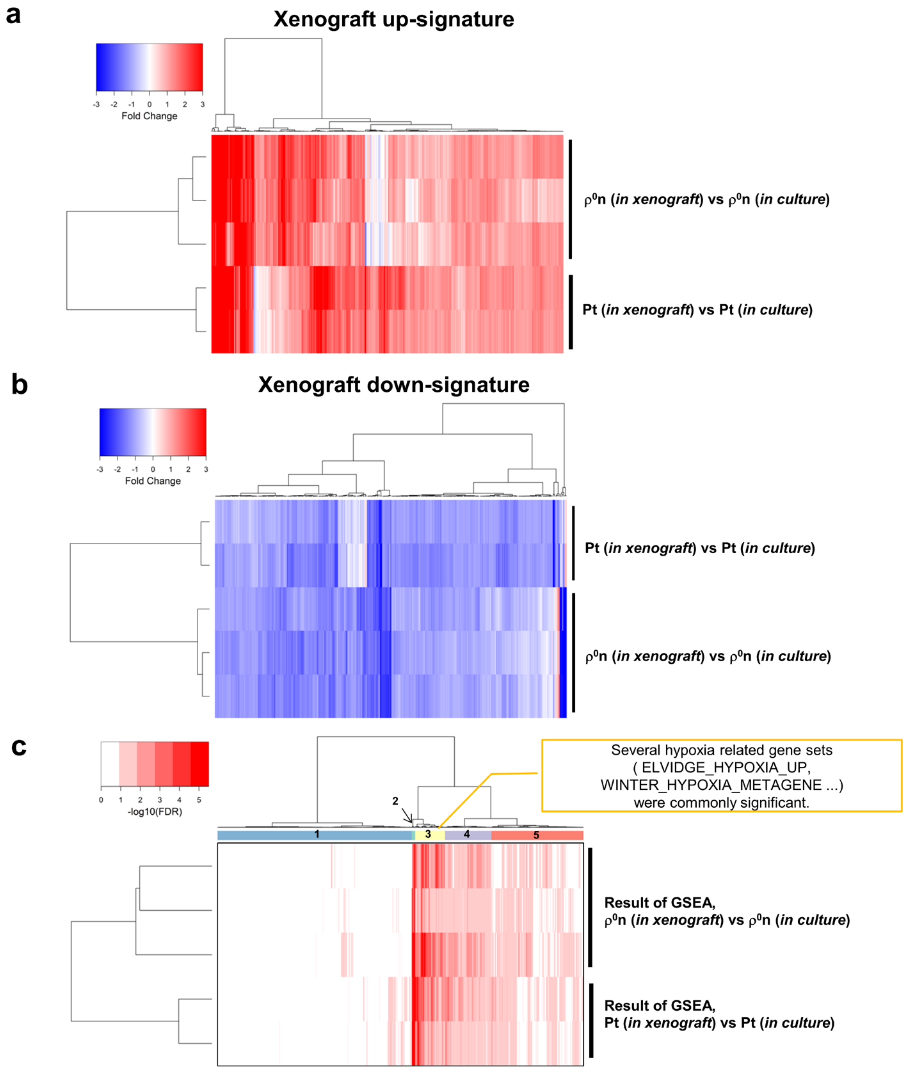

Supplementary Figure 1: Similarity of gene expression change of HT-29 Pt cells or ρ^0n cells in xenograft with those in culture. a, b. Signal intensity ratio of gene expression. HT-29 Pt and HT-29 ρ^0n cells in xenograft were compared with those cells in culture, respectively. We selected 496 up-regulated probe sets (303 down-regulated probe sets) as follows:

Step 1. Selected commonly up-regulated (down-regulated) probe sets in two samples of xenografts of HT-29 Pt cells.

Step 2. Selected commonly up-regulated (down-regulated) probe sets in three samples of HT-29 Pt cell xenografts.

Step 3. Selected any of the probe sets in Steps 1 and 2 in each direction, and named them xenograft up-signature (down-signature).

Xenograft up-signature or down-signature were used in (a) and (b), respectively (see gene lists in Supplementary Table 10 and 11.). **c.** Summary of GSEA comparing xenografts of HT-29 Pt or HT-29 ρ^0n cells with those in culture, using the gene sets of MSigDB c2 collections. The log-scaled FDR of gene set enrichment was showed in the heat map with unsupervised clustering, where the numbers in the upper color bar indicate the clustered signatures. If the antilogarithm of FDR was 0, we replaced the value by the second smallest FDR.

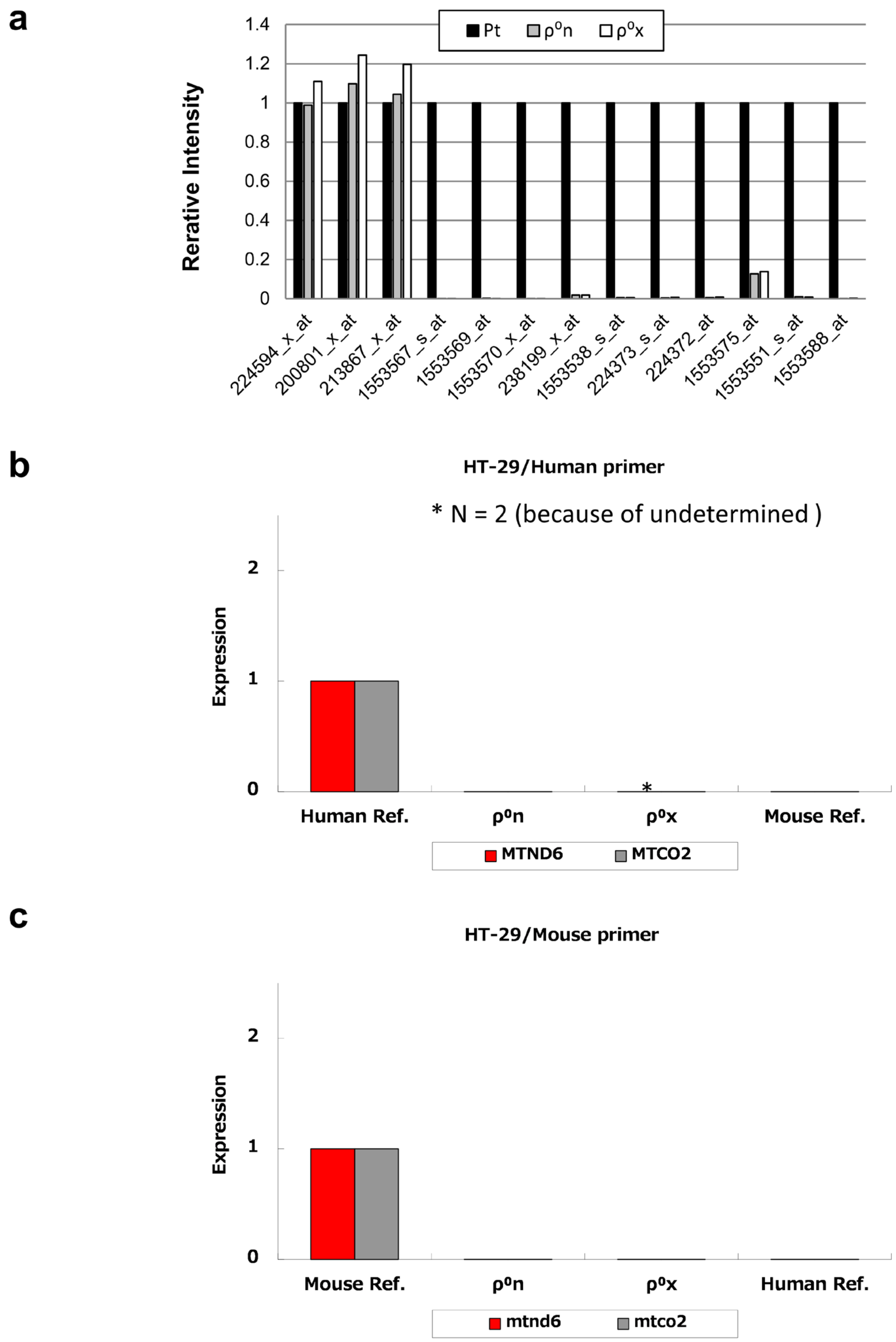

Supplementary Figure 2: Absence of mtDNA in HT-29 ρ^{0}x cells. a. Probe set of mtDNA in HT-29 cells. The signal intensity was normalized by HT-29 Pt cells. Probes representing 224594_x_at, 200801_x_at and 213867_x_at detected *ACTB*. Other probes detected several mtDNA genes (Supplementary Table 9). **b, c.** qRT-PCR analysis of *MTCO2*, using primers designed for human in (b) or mouse in (c). Expression levels were compared with reference RNA of human in (b) or mouse in (c).
Error bars indicate SD, * one sample was undetermined, because signal intensity was severely low.

a

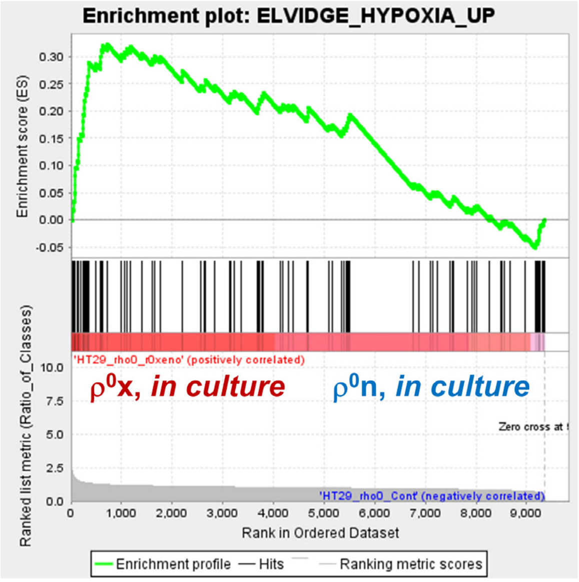

Supplementary Figure 3: Enriched hypoxia-related signature in HT-29 ρ^0x cells compared with HT-29 ρ^0n cells. Enrichment plot of GSEA, using a signature of ‘ELVIDGE_HYPOXIA_UP’, up-regulated in breast cancer MCF7 cells under hypoxic conditions [1]. Detail result of GSEA was shown in Supplementary Table 7 and 8.

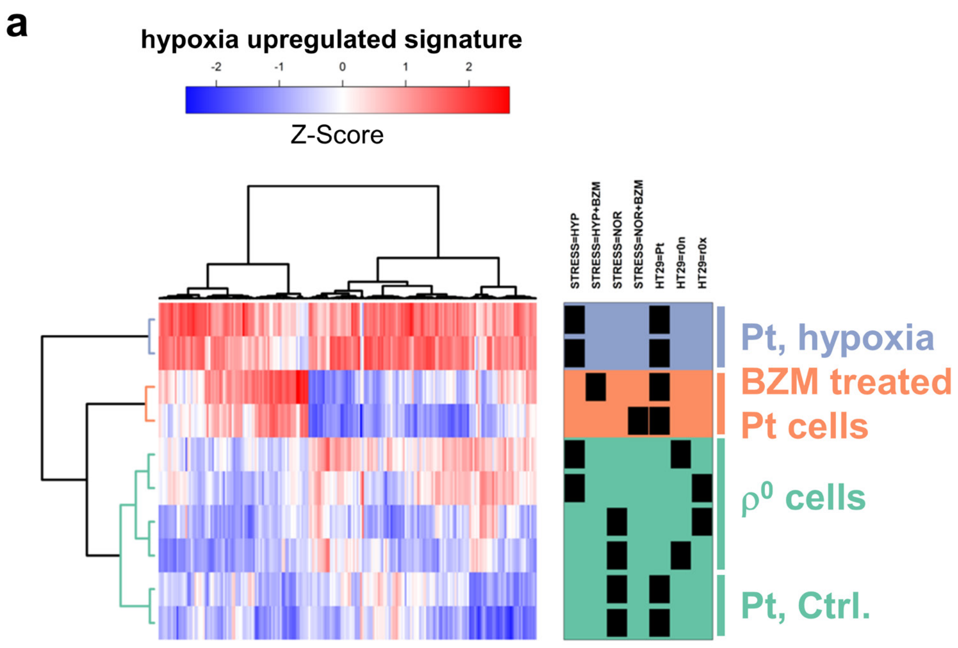
a
hypoxia upregulated signature
-2
-1
0
1
2
Z-Score
STRESS=HYP
STRESS=HYP+BZM
STRESS=NOR
STRESS=NOR+BZM
HT29=Pt
HT29=r0n
HT29=r0x
Pt, hypoxia
BZM treated
Pt cells
ρ0 cells
Pt, Ctrl.

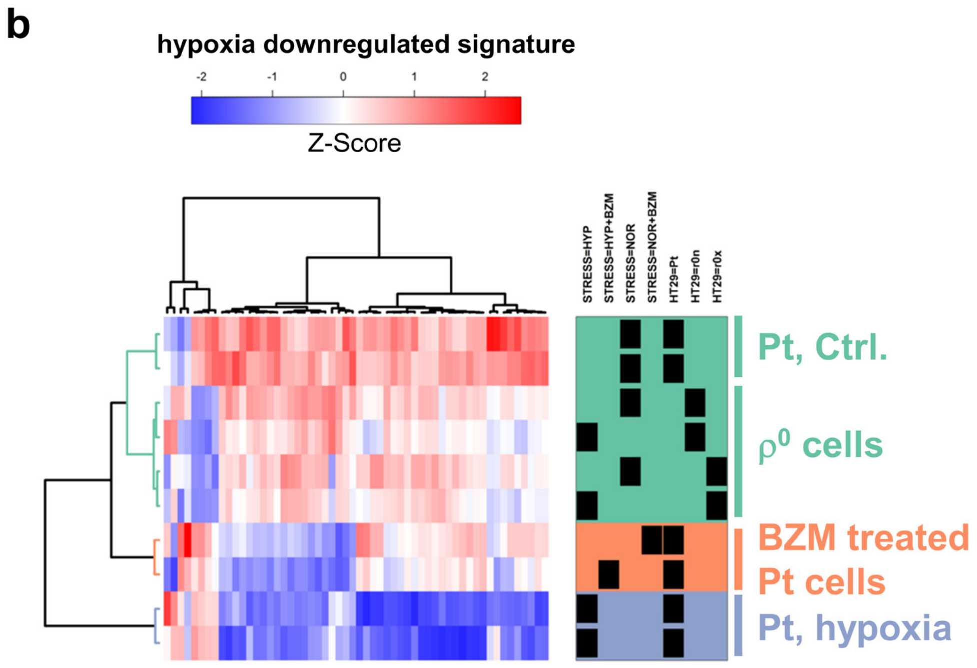
b
hypoxia downregulated signature
-2
-1
0
1
2
Z-Score
STRESS=HYP
STRESS=HYP+BZM
STRESS=NOR
STRESS=NOR+BZM
HT29=Pt
HT29=r0n
HT29=r0x
Pt, Ctrl.
ρ0 cells
BZM treated
Pt cells
Pt, hypoxia

(Continued)

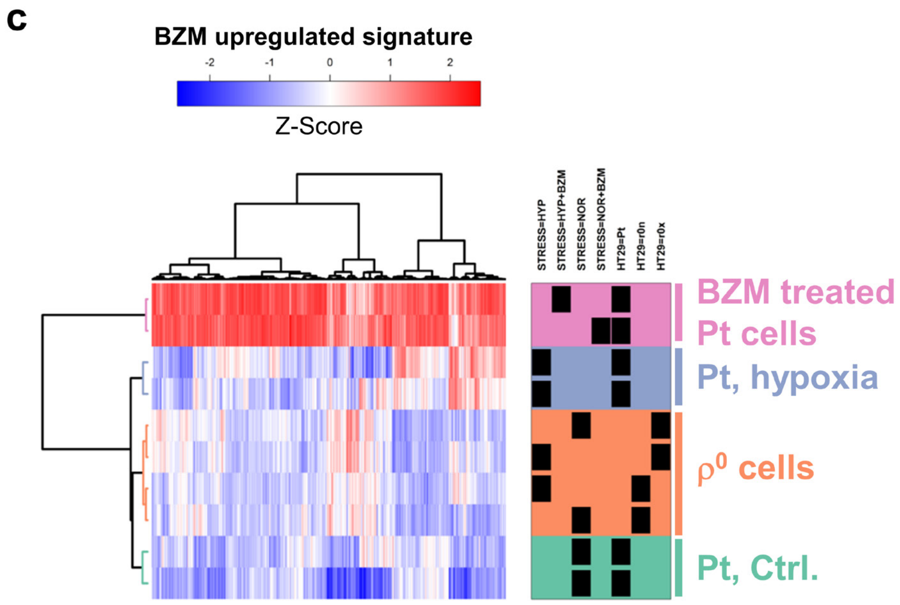

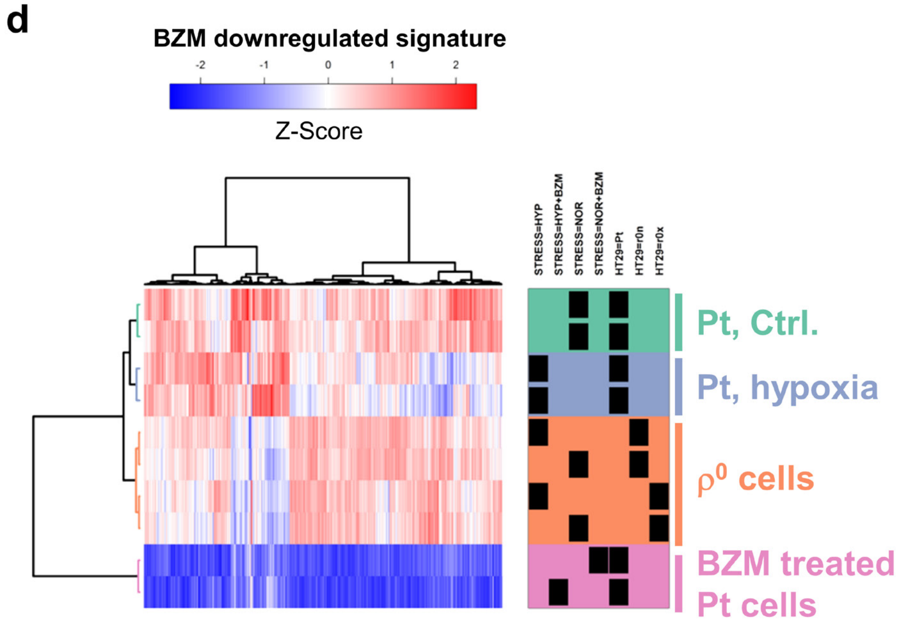

Supplementary Figure 4: Gene expression levels of HT29 cells in hypoxia signature. a-d. Gene expression levels of samples used in Figure 4 (b) and (c). Signal intensities were converted to a z-score by each probe and shown using an hypoxia upregulated signature in (a), hypoxia downregulated signature in (b), BZM upregulated signature in (c) and BZM downregulated signature in (d). Right panel shows the annotation of samples.

STRESS=HYP: cells were cultured under 18 hours of hypoxia.
STRESS=HYP+BZM: cells treated with bortezomib (BZM) were cultured under 18 hours of hypoxia.
STRESS=NOR: cells were cultured under 18 hours of normoxia.
STRESS=NOR+BZM: cells treated with bortezomib (BZM) were cultured under 18 hours of normoxia
HT29=Pt: HT-29 Pt cells.
HT29=r0n: HT-29 ρ^0n cells.
HT29=r0x: HT-29 ρ^0x cells.

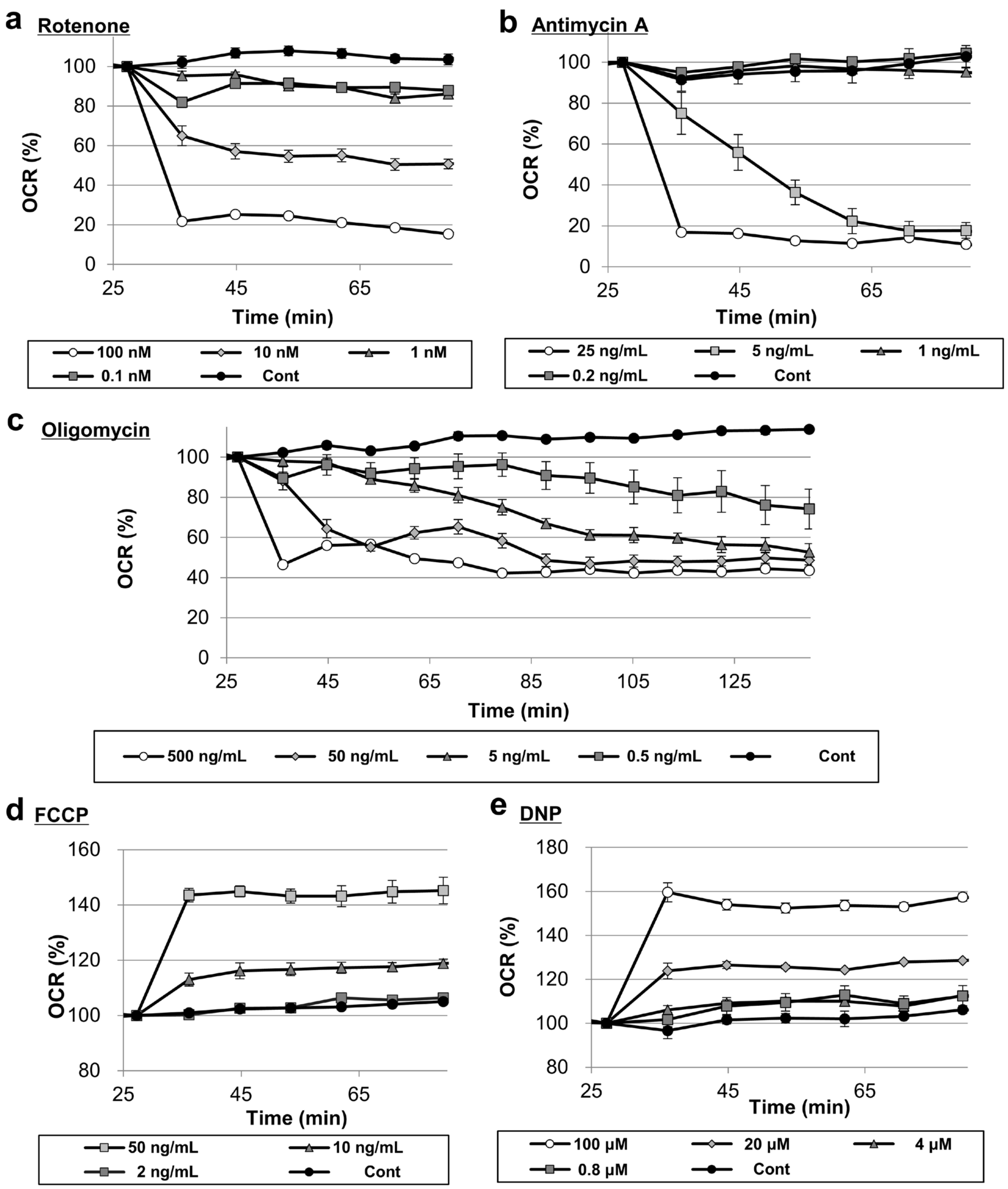

Supplementary Figure 5: Inhibition of mitochondrial oxidative phosphorylation by several compounds. Supporting information of Figure 5. **a-e.** OCR of HT-29 Pt cells was measured after treatment with the indicated concentrations of rotenone in (a), antimycin A in (b), oligomycin in (c), FCCP in (d) and DNP in (e). OCR was described as relative value to the untreated state.

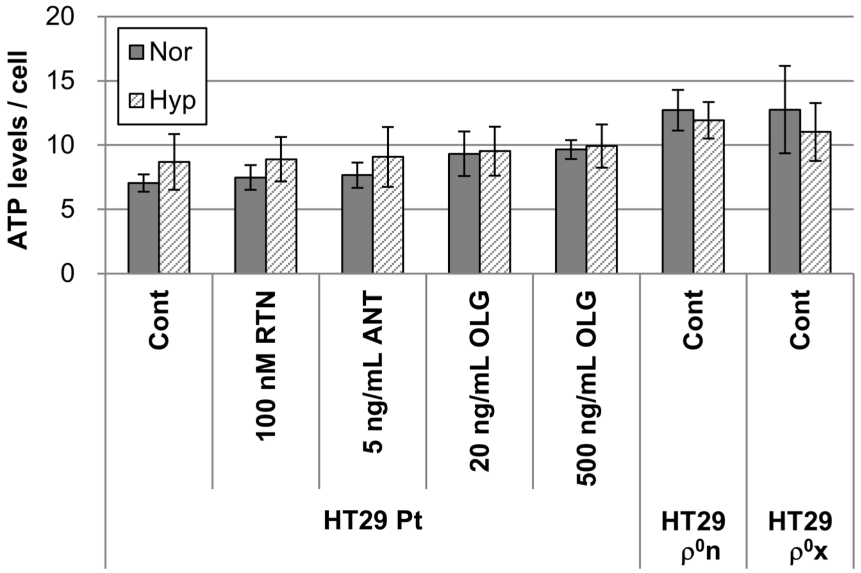

Supplementary Figure 6: Keeping intra-cellular ATP levels under hypoxia with mitochondrial inhibition. Intra-cellular ATP levels of HT-29 Pt, ρ^0n and ρ^0x cells, normalized by each cell numbers. Cells were treated with 18 hours hypoxia (Hyp) and normoxia (Nor). RTN, rotenone; ANT, antimycin A; OLG, oligomycin. Data were presented as mean ± SD of three independent experiments performed in triplicate.

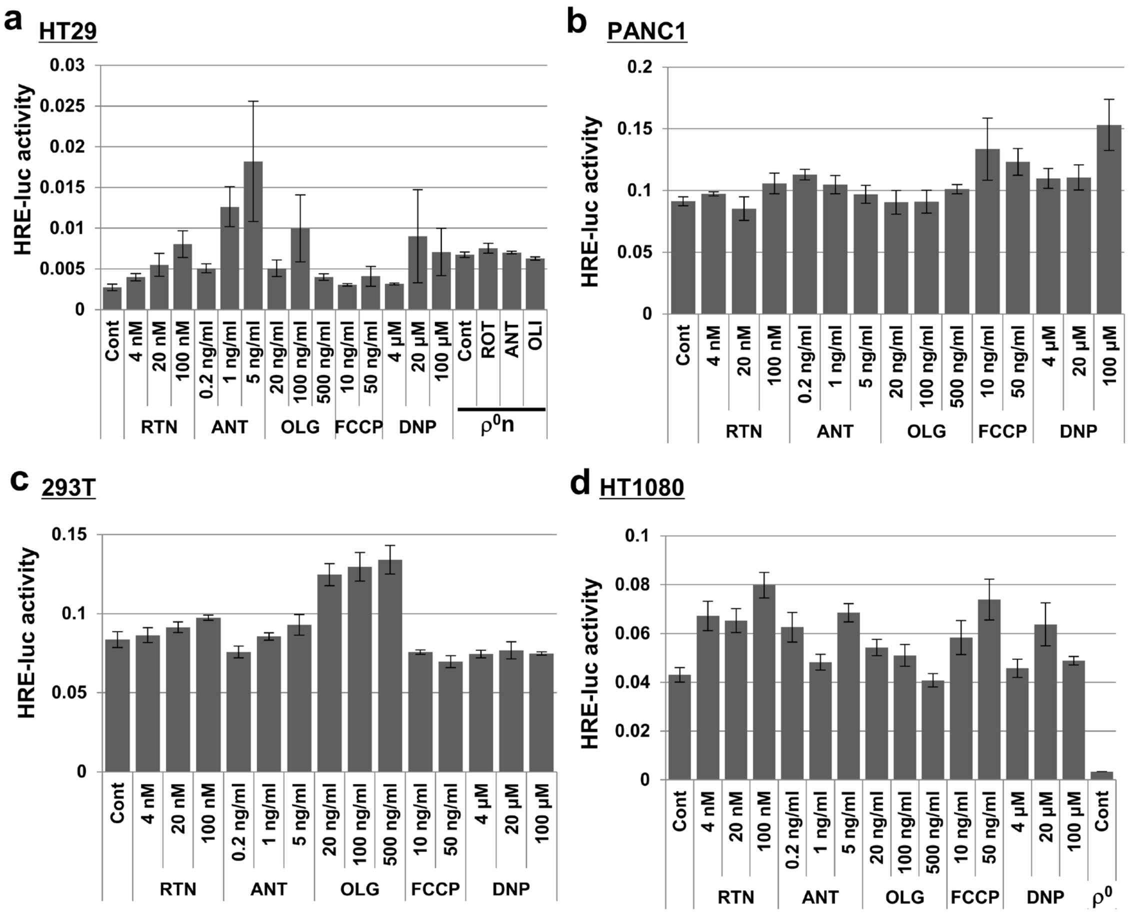

Supplementary Figure 7: HRE-luc activity treating mitochondrial inhibitors under normoxia. a-d. HRE-luc activity in HT-29 (a), PANC1 (b), 293T (c) and HT1080 cells (d) under normoxia. These data were from Figure 6a–d.

Supplementary Table 1: Hypoxia up-regulated signature

See Supplementary File 1

Supplementary Table 2: Hypoxia down-regulated signature

See Supplementary File 1

Supplementary Table 3: ρ^0x Up-regulated signature

See Supplementary File 1

Supplementary Table 4: ρ^0x Down-regulated signature

Probe.ID	NAME
1554997_a_at	PTGS2
1569454_a_at	LOC283352
202437_s_at	CYP1B1
203065_s_at	CAV1
203323_at	CAV2
203324_s_at	CAV2
203691_at	PI3
203963_at	CA12
204151_x_at	AKR1C1
204446_s_at	ALOX5
204508_s_at	CA12
204588_s_at	SLC7A7
204607_at	HMGCS2
204748_at	PTGS2
205111_s_at	PLCE1
205919_at	HBE1
206561_s_at	AKR1B10
206737_at	WNT11
207076_s_at	ASS1
210145_at	PLA2G4A
212097_at	CAV1
219508_at	GCNT3
221530_s_at	BHLHE41
223204_at	FAM198B
225270_at	NEO1
226038_at	LONRF1
230250_at	PTPRB
242093_at	SYTL5
41469_at	PI3

The 29 probe sets down-regulated in HT-29 ρ^0x cells compared with HT-29 ρ^0n cells.

Supplementary Table 5: ρ^0n_vivo Up-regulated signature

See Supplementary File 1

Supplementary Table 6: ρ^0n_vivo Down-regulated signature

See Supplementary File 1

Supplementary Table 7: Enrichment result, up-regulated c2 signatures in HT-29 ρ^0x cells compared with in HT-29 ρ^0n cells

See Supplementary File 1

Supplementary Table 8: Enrichment result, up-regulated c2 signatures in HT-29 ρ^0n cells compared with in HT-29 ρ^0x cells.

See Supplementary File 1

Supplementary Table 9: Probe sets recognizing mtDNA

Probe.ID	NAME
1553567_s_at	ATP6
1553569_at	COX2
1553570_x_at	COX2
238199_x_at	COX3
1553538_s_at	COX1
224373_s_at	DCAF6///HNRNPM///ND4
224372_at	DCAF6///ND4
1553575_at	ND6
1553551_s_at	ND2
1553588_at	ND3///SH3KBP1

Supporting information of Supplementary Figure 2a. These probe sets recognize the mRNA of mtDNA.

Supplementary Table 10: Xenograft up-regulated signature

See Supplementary File 1

Supplementary Table 11: Xenograft down-regulated signature

See Supplementary File 1

Supplementary Table 12: BZM upregulated signature

See Supplementary File 1

Supplementary Table 13: BZM downregulated signature

See Supplementary File 1
