# Supplementary material for: Mitochondrial deficiency impairs hypoxic induction of HIF-1 transcriptional activity and retards tumor growth
Source: Oncotarget. 2017 Jan 2;8(7):11841–54. doi: 10.18632/oncotarget.14415 (PMC5355308; doi:10.18632/oncotarget.14415)
Supplement: Supplementary file 2 [file oncotarget-08-11841-s002.docx]

## Supplementary Table 1. Hypoxia up-regulated signature

212 hypoxia-dependent up-regulated probe sets, in HT-29 Pt cells, HT-29 Pt cells with BZM, HT-29 ρ^0^n cells or HT-29 ρ^0^x cells.

| **Probe.ID** | **NAME** |
| --- | --- |
| 1552799_at | TSNARE1 |
| 1553538_s_at | COX1 |
| 1554436_a_at | REG4 |
| 1555832_s_at | KLF6 |
| 1556123_a_at | RAB11B-AS1 |
| 1568611_at | --- |
| 200632_s_at | NDRG1 |
| 200696_s_at | GSN |
| 200737_at | PGK1 |
| 200878_at | EPAS1 |
| 200920_s_at | BTG1 |
| 200985_s_at | CD59 |
| 201008_s_at | LOC101060503 /// TXNIP |
| 201009_s_at | LOC101060503 /// TXNIP |
| 201010_s_at | LOC101060503 /// TXNIP |
| 201105_at | LGALS1 |
| 201170_s_at | BHLHE40 |
| 201242_s_at | ATP1B1 |
| 201243_s_at | ATP1B1 |
| 201250_s_at | SLC2A1 |
| 201313_at | ENO2 |
| 201464_x_at | JUN |
| 201465_s_at | JUN |
| 201466_s_at | JUN |
| 201482_at | QSOX1 |
| 201565_s_at | ID2 |
| 201578_at | PODXL |
| 201625_s_at | INSIG1 |
| 201626_at | INSIG1 |
| 201627_s_at | INSIG1 |
| 201650_at | KRT19 |
| 201694_s_at | EGR1 |
| 202022_at | ALDOC |
| 202130_at | RIOK3 |
| 202131_s_at | RIOK3 |
| 202219_at | SLC6A8 |
| 202336_s_at | PAM |
| 202364_at | MXI1 |
| 202388_at | RGS2 |
| 202464_s_at | PFKFB3 |
| 202489_s_at | FXYD3 |
| 202619_s_at | PLOD2 |
| 202620_s_at | PLOD2 |
| 202668_at | EFNB2 |
| 202672_s_at | ATF3 |
| 202733_at | P4HA2 |
| 202769_at | CCNG2 |
| 202770_s_at | CCNG2 |
| 202856_s_at | SLC16A3 |
| 202859_x_at | IL8 |
| 202887_s_at | DDIT4 |
| 202912_at | ADM |
| 202934_at | HK2 |
| 202973_x_at | FAM13A |
| 203065_s_at | CAV1 |
| 203108_at | GPRC5A |
| 203282_at | GBE1 |
| 203509_at | SORL1 |
| 203585_at | ZNF185 |
| 203726_s_at | LAMA3 |
| 203757_s_at | CEACAM6 |
| 203851_at | IGFBP6 |
| 204030_s_at | IQCJ-SCHIP1 /// SCHIP1 |
| 204194_at | BACH1 |
| 204268_at | S100A2 |
| 204446_s_at | ALOX5 |
| 204607_at | HMGCS2 |
| 204698_at | ISG20 |
| 204790_at | SMAD7 |
| 204818_at | HSD17B2 |
| 204990_s_at | ITGB4 |
| 205076_s_at | MTMR11 |
| 205128_x_at | PTGS1 |
| 205199_at | CA9 |
| 205398_s_at | SMAD3 |
| 205759_s_at | SULT2B1 |
| 205765_at | CYP3A5 |
| 205780_at | BIK |
| 205807_s_at | TUFT1 |
| 205822_s_at | HMGCS1 |
| 205846_at | PTPRB |
| 206172_at | IL13RA2 |
| 206969_at | KRT34 /// LOC100653049 |
| 207196_s_at | TNIP1 |
| 207543_s_at | P4HA1 |
| 207574_s_at | GADD45B |
| 208296_x_at | TNFAIP8 |
| 208483_x_at | KRT33A |
| 208937_s_at | ID1 |
| 208961_s_at | KLF6 |
| 209125_at | KRT6A |
| 209189_at | FOS |
| 209270_at | LAMB3 |
| 209373_at | MALL |
| 209383_at | DDIT3 |
| 209403_at | LOC100510707 /// LOC101060287 /// LOC101060303 /// LOC101060321 /// LOC101060351 /// LOC101060367 /// LOC101060376 /// LOC101060389 /// LOC101060403 /// LOC101060421 /// LOC101060440 /// LOC101060471 /// LOC101060489 /// LOC101060506 /// LOC101060522 /// TBC1D3 /// TBC1D3C /// TBC1D3F /// TBC1D3G /// TBC1D3H |
| 209546_s_at | APOL1 |
| 209566_at | INSIG2 |
| 209608_s_at | ACAT2 |
| 210260_s_at | TNFAIP8 |
| 210276_s_at | TRIOBP |
| 210512_s_at | VEGFA |
| 210827_s_at | ELF3 |
| 210845_s_at | PLAUR |
| 210854_x_at | SLC6A8 |
| 211657_at | CEACAM6 |
| 211719_x_at | FN1 |
| 211924_s_at | PLAUR |
| 212097_at | CAV1 |
| 212274_at | LPIN1 |
| 212276_at | LPIN1 |
| 212444_at | GPRC5A |
| 212463_at | CD59 |
| 212496_s_at | KDM4B |
| 212560_at | SORL1 |
| 212689_s_at | KDM3A |
| 212958_x_at | PAM |
| 213349_at | TMCC1 |
| 213351_s_at | TMCC1 |
| 213680_at | KRT6B |
| 213693_s_at | MUC1 |
| 213843_x_at | SLC6A8 |
| 213953_at | KRT20 |
| 214079_at | DHRS2 |
| 214620_x_at | PAM |
| 215812_s_at | LOC653562 /// SLC6A10P /// SLC6A8 |
| 215813_s_at | PTGS1 |
| 216210_x_at | TRIOBP |
| 217047_s_at | FAM13A |
| 217356_s_at | PGK1 |
| 217691_x_at | SLC16A3 |
| 217875_s_at | PMEPA1 |
| 217997_at | PHLDA1 |
| 218205_s_at | MKNK2 |
| 218484_at | NDUFA4L2 |
| 218498_s_at | ERO1L |
| 218559_s_at | MAFB |
| 218791_s_at | KATNBL1 |
| 218858_at | DEPTOR |
| 219529_at | CLIC3 |
| 219634_at | CHST11 |
| 219888_at | SPAG4 |
| 220030_at | STYK1 |
| 221478_at | BNIP3L |
| 221479_s_at | BNIP3L |
| 221577_x_at | GDF15 |
| 221653_x_at | APOL2 |
| 221841_s_at | KLF4 |
| 222108_at | AMIGO2 |
| 222408_s_at | YPEL5 |
| 222449_at | PMEPA1 |
| 222450_at | PMEPA1 |
| 222646_s_at | ERO1L |
| 222745_s_at | KATNBL1 |
| 222847_s_at | EGLN3 |
| 223046_at | EGLN1 |
| 223193_x_at | FAM162A |
| 223199_at | MKNK2 |
| 223447_at | REG4 |
| 223541_at | HAS3 |
| 224314_s_at | EGLN1 |
| 224345_x_at | FAM162A |
| 224565_at | MIR612 /// NEAT1 |
| 224566_at | MIR612 /// NEAT1 |
| 224602_at | C4orf3 |
| 224604_at | C4orf3 |
| 224605_at | C4orf3 |
| 224606_at | KLF6 |
| 224657_at | ERRFI1 |
| 224797_at | ARRDC3 |
| 225136_at | PLEKHA2 |
| 225239_at | MIR612 /// NEAT1 |
| 225262_at | FOSL2 |
| 225342_at | AK4 /// LOC100507855 |
| 225544_at | TBX3 |
| 225626_at | PAG1 |
| 225750_at | ERO1L |
| 225898_at | WDR54 |
| 226226_at | TMEM45B |
| 226347_at | --- |
| 226348_at | --- |
| 226403_at | TMC4 |
| 226452_at | PDK1 |
| 226535_at | ITGB6 |
| 226706_at | FLJ23867 /// QSOX1 |
| 227020_at | YPEL2 |
| 227068_at | PGK1 |
| 227112_at | TMCC1 |
| 227314_at | ITGA2 |
| 227337_at | ANKRD37 |
| 227556_at | NME7 |
| 227759_at | PCSK9 |
| 228188_at | FOSL2 |
| 228483_s_at | TAF9B |
| 228499_at | PFKFB4 |
| 228919_at | --- |
| 229030_at | CAPN8 |
| 230250_at | PTPRB |
| 230323_s_at | TMEM45B |
| 230710_at | MIR210HG |
| 234312_s_at | ACSS2 |
| 234725_s_at | SEMA4B |
| 235153_at | RNF183 |
| 235419_at | --- |
| 235857_at | KCTD11 |
| 238542_at | ULBP2 |
| 239202_at | RAB3B |
| 239370_at | LOC100505633 |
| 242722_at | LMO7 |
| 36711_at | MAFF |
| 37152_at | PPARD |
| 91826_at | EPS8L1 |

## Supplementary Table 2. Hypoxia down-regulated signature

56 hypoxia-dependent down-regulated probe sets, in HT-29 Pt cells, HT-29 Pt cells with BZM, HT-29 ρ^0^n cells or HT-29 ρ^0^x cells.

| **Probe.ID** | **NAME** |
| --- | --- |
| 1553551_s_at | ND2 |
| 200894_s_at | FKBP4 |
| 200895_s_at | FKBP4 |
| 200987_x_at | PSME3 |
| 201516_at | SRM |
| 203023_at | NOP16 |
| 203119_at | CCDC86 |
| 203622_s_at | PNO1 |
| 204405_x_at | DIMT1 |
| 205129_at | NPM3 |
| 205136_s_at | NUFIP1 |
| 205264_at | CD3EAP |
| 205311_at | DDC |
| 205767_at | EREG |
| 205895_s_at | NOLC1 |
| 206755_at | CYP2B6 |
| 209567_at | RRS1 |
| 209735_at | ABCG2 |
| 209853_s_at | PSME3 |
| 210802_s_at | DIMT1 |
| 212510_at | GPD1L |
| 212973_at | LOC101060545 /// RPIA |
| 213320_at | PRMT3 |
| 214011_s_at | NOP16 |
| 217106_x_at | DIMT1 |
| 217127_at | CTH |
| 218512_at | WDR12 |
| 218695_at | EXOSC4 |
| 218866_s_at | POLR3K |
| 218868_at | ACTR3B |
| 218882_s_at | WDR3 |
| 218886_at | PAK1IP1 |
| 218889_at | NOC3L |
| 219006_at | NDUFAF4 |
| 219037_at | RRP15 |
| 219420_s_at | SELRC1 |
| 220468_at | ARL14 |
| 222108_at | AMIGO2 |
| 222883_at | SELRC1 |
| 223403_s_at | POLR1B |
| 223414_s_at | LYAR |
| 224467_s_at | PDCD2L |
| 224632_at | GPATCH4 |
| 224634_at | GPATCH4 |
| 225291_at | PNPT1 |
| 225593_at | LSM10 |
| 225712_at | GEMIN5 |
| 225748_at | LTV1 |
| 226037_s_at | TAF9B |
| 226410_at | CTU2 |
| 226479_at | KBTBD6 |
| 226998_at | NAA15 |
| 228050_at | UTP15 |
| 229332_at | HPDL |
| 242093_at | SYTL5 |
| 242260_at | MATR3 |

## Supplementary Table 3. ρ^0^x Up-regulated signature.

The 69 probe sets up-regulated in HT-29 ρ^0^x cells compared with HT-29 ρ^0^n cells.

| **Probe.ID** | **NAME** |
| --- | --- |
| 201311_s_at | SH3BGRL |
| 201312_s_at | SH3BGRL |
| 201315_x_at | IFITM2 |
| 201426_s_at | VIM |
| 201506_at | TGFBI |
| 201939_at | PLK2 |
| 202207_at | ARL4C |
| 202458_at | PRSS23 |
| 202686_s_at | AXL |
| 203083_at | THBS2 |
| 203139_at | DAPK1 |
| 203180_at | ALDH1A3 |
| 203638_s_at | FGFR2 |
| 203675_at | NUCB2 |
| 203988_s_at | FUT8 |
| 204222_s_at | GLIPR1 |
| 204235_s_at | GULP1 |
| 204237_at | GULP1 |
| 205780_at | BIK |
| 206463_s_at | DHRS2 |
| 206560_s_at | MIA |
| 206632_s_at | APOBEC3B |
| 207325_x_at | MAGEA1 |
| 209276_s_at | GLRX |
| 209310_s_at | CASP4 |
| 209576_at | GNAI1 |
| 209792_s_at | KLK10 |
| 210139_s_at | PMP22 |
| 210538_s_at | BIRC3 |
| 211518_s_at | BMP4 |
| 212077_at | CALD1 |
| 212098_at | MGAT5 |
| 212203_x_at | IFITM3 |
| 212224_at | ALDH1A1 |
| 212509_s_at | MXRA7 |
| 214079_at | DHRS2 |
| 214085_x_at | GLIPR1 |
| 214774_x_at | TOX3 |
| 215108_x_at | TOX3 |
| 215913_s_at | GULP1 |
| 216623_x_at | TOX3 |
| 217691_x_at | SLC16A3 |
| 217995_at | SQRDL |
| 218025_s_at | ECI2 |
| 218633_x_at | ABHD10 |
| 218705_s_at | SNX24 |
| 218872_at | TESC |
| 218967_s_at | PTER |
| 219387_at | CCDC88A |
| 220180_at | CCDC68 |
| 221577_x_at | GDF15 |
| 222572_at | PDP1 |
| 222697_s_at | ABHD10 |
| 222803_at | PRTFDC1 |
| 223551_at | PKIB |
| 224560_at | TIMP2 |
| 224920_x_at | MYADM |
| 225496_s_at | SYTL2 |
| 225673_at | MYADM |
| 226279_at | PRSS23 |
| 227692_at | GNAI1 |
| 228489_at | TM4SF18 |
| 230061_at | TM4SF18 |
| 231579_s_at | TIMP2 |
| 232914_s_at | SYTL2 |
| 235696_at | OTTHUMG00000163952 /// RP11-11N9.4 |
| 235700_at | CT45A1 /// CT45A2 /// CT45A3 /// CT45A4 /// CT45A5 /// CT45A6 /// LOC101060208 /// LOC101060210 /// LOC101060211 |
| 238017_at | SDR16C5 |
| 39248_at | AQP3 |

## Supplementary Table 5. ρ^0^n_vivo Up-regulated signature.

The 213 probe sets up-regulated in xenografts of HT-29 ρ^0^n cells compared with HT-29 ρ^0^n cells in culture.

| **Probe.ID** | **NAME** |
| --- | --- |
| 1553185_at | RASEF |
| 1553186_x_at | RASEF |
| 1553611_s_at | KLHL35 |
| 1554600_s_at | LMNA |
| 1555745_a_at | LYZ |
| 1555832_s_at | KLF6 |
| 1555851_s_at | SEPW1 |
| 1557987_at | --- |
| 1569454_a_at | LOC283352 |
| 200634_at | PFN1 |
| 200660_at | S100A11 |
| 200771_at | LAMC1 |
| 200852_x_at | GNB2 |
| 200859_x_at | FLNA |
| 200920_s_at | BTG1 |
| 200921_s_at | BTG1 |
| 201010_s_at | LOC101060503 /// TXNIP |
| 201040_at | GNAI2 |
| 201162_at | IGFBP7 |
| 201188_s_at | ITPR3 |
| 201194_at | SEPW1 |
| 201242_s_at | ATP1B1 |
| 201243_s_at | ATP1B1 |
| 201310_s_at | NREP |
| 201311_s_at | SH3BGRL |
| 201312_s_at | SH3BGRL |
| 201315_x_at | IFITM2 |
| 201369_s_at | ZFP36L2 |
| 201426_s_at | VIM |
| 201427_s_at | SEPP1 |
| 201464_x_at | JUN |
| 201564_s_at | FSCN1 |
| 201601_x_at | IFITM1 /// IFITM2 |
| 201655_s_at | HSPG2 |
| 201694_s_at | EGR1 |
| 201841_s_at | HSPB1 |
| 201876_at | PON2 |
| 201939_at | PLK2 |
| 202102_s_at | BRD4 |
| 202411_at | IFI27 |
| 202761_s_at | SYNE2 |
| 202833_s_at | SERPINA1 |
| 203020_at | RABGAP1L |
| 203139_at | DAPK1 |
| 203186_s_at | S100A4 |
| 203304_at | BAMBI |
| 203394_s_at | HES1 |
| 203638_s_at | FGFR2 |
| 203688_at | PKD2 |
| 203725_at | GADD45A |
| 203752_s_at | JUND |
| 203753_at | TCF4 |
| 203799_at | CD302 /// LY75-CD302 |
| 203882_at | IRF9 |
| 203953_s_at | CLDN3 |
| 203961_at | NEBL |
| 204235_s_at | GULP1 |
| 204237_at | GULP1 |
| 204259_at | MMP7 |
| 204272_at | LGALS4 |
| 204379_s_at | FGFR3 |
| 204415_at | IFI6 |
| 204622_x_at | NR4A2 |
| 204713_s_at | F5 |
| 204714_s_at | F5 |
| 204806_x_at | HLA-F |
| 205137_x_at | USH1C |
| 205506_at | VIL1 |
| 205513_at | TCN1 |
| 206239_s_at | SPINK1 |
| 206560_s_at | MIA |
| 206632_s_at | APOBEC3B |
| 206858_s_at | HOXC6 |
| 207011_s_at | PTK7 |
| 207173_x_at | CDH11 |
| 208228_s_at | FGFR2 |
| 208436_s_at | IRF7 |
| 208540_x_at | S100A11P1 /// S100A11P1 |
| 208729_x_at | HLA-B |
| 208960_s_at | KLF6 |
| 208961_s_at | KLF6 |
| 208997_s_at | UCP2 |
| 209173_at | AGR2 |
| 209201_x_at | CXCR4 |
| 209212_s_at | KLF5 |
| 209403_at | LOC100510707 /// LOC101060287 /// LOC101060303 /// LOC101060321 /// LOC101060351 /// LOC101060367 /// LOC101060376 /// LOC101060389 /// LOC101060403 /// LOC101060421 /// LOC101060440 /// LOC101060471 /// LOC101060489 /// LOC101060506 /// LOC101060522 /// TBC1D3 /// TBC1D3C /// TBC1D3F /// TBC1D3G /// TBC1D3H |
| 209417_s_at | IFI35 |
| 209498_at | CEACAM1 |
| 209504_s_at | PLEKHB1 |
| 209911_x_at | HIST1H2BD |
| 210046_s_at | IDH2 |
| 210105_s_at | FYN |
| 210117_at | SPAG1 |
| 210230_at | --- |
| 210445_at | FABP6 |
| 210592_s_at | SAT1 |
| 210797_s_at | OASL |
| 210830_s_at | PON2 |
| 211160_x_at | ACTN1 |
| 211184_s_at | USH1C |
| 211429_s_at | SERPINA1 |
| 211528_x_at | HLA-G |
| 211919_s_at | CXCR4 |
| 211950_at | UBR4 |
| 211964_at | COL4A2 |
| 212076_at | KMT2A |
| 212077_at | CALD1 |
| 212080_at | KMT2A |
| 212099_at | RHOB |
| 212151_at | PBX1 |
| 212203_x_at | IFITM3 |
| 212224_at | ALDH1A1 |
| 212236_x_at | JUP /// KRT17 |
| 212263_at | QKI |
| 212338_at | MYO1D |
| 212386_at | TCF4 |
| 213142_x_at | GSAP |
| 213158_at | OTTHUMG00000175755 /// RP11-553L6.5 |
| 213605_s_at | --- |
| 213695_at | PON3 |
| 213746_s_at | FLNA |
| 214022_s_at | IFITM1 |
| 214290_s_at | HIST2H2AA3 /// HIST2H2AA4 |
| 214459_x_at | HLA-C |
| 214693_x_at | LOC101059961 /// LOC101060202 /// LOC101060362 /// LOC101060684 /// NBPF10 /// NBPF12 /// NBPF9 |
| 214752_x_at | FLNA |
| 214774_x_at | TOX3 |
| 215071_s_at | HIST1H2AC |
| 215076_s_at | COL3A1 |
| 215108_x_at | TOX3 |
| 215127_s_at | RBMS1 |
| 215383_x_at | SPG21 |
| 216405_at | --- |
| 216563_at | ANKRD12 |
| 216623_x_at | TOX3 |
| 217028_at | CXCR4 |
| 218164_at | SPATA20 |
| 218309_at | CAMK2N1 |
| 218322_s_at | ACSL5 |
| 218831_s_at | FCGRT |
| 218872_at | TESC |
| 218962_s_at | TMEM168 |
| 219878_s_at | KLF13 |
| 220755_s_at | C6orf48 |
| 221004_s_at | ITM2C |
| 221577_x_at | GDF15 |
| 221764_at | R3HDM4 |
| 221875_x_at | HLA-F |
| 222150_s_at | GSAP |
| 222408_s_at | YPEL5 |
| 222803_at | PRTFDC1 |
| 223103_at | STARD10 |
| 223125_s_at | C1orf21 |
| 223218_s_at | NFKBIZ |
| 223497_at | FAM135A |
| 224159_x_at | TRIM4 |
| 224344_at | COX6A1 |
| 224428_s_at | CDCA7 |
| 224549_x_at | --- |
| 224559_at | MALAT1 |
| 224606_at | KLF6 |
| 224741_x_at | GAS5 /// SNORD44 /// SNORD47 /// SNORD76 /// SNORD77 /// SNORD79 /// SNORD80 /// SNORD81 |
| 224841_x_at | GAS5 /// SNORD44 /// SNORD47 /// SNORD76 /// SNORD77 /// SNORD79 /// SNORD80 /// SNORD81 |
| 224915_x_at | ZFAS1 |
| 225033_at | ST3GAL1 |
| 225056_at | SIPA1L2 |
| 225093_at | UTRN |
| 225239_at | MIR612 /// NEAT1 |
| 225496_s_at | SYTL2 |
| 225628_s_at | MLLT6 |
| 225698_at | EPB41L4A-AS1 |
| 226123_at | CHD7 |
| 226227_x_at | ZFAS1 |
| 226275_at | MXD1 |
| 226459_at | PIK3AP1 |
| 226560_at | --- |
| 226575_at | ZNF462 |
| 226835_s_at | ZFAS1 |
| 226844_at | MOB3B |
| 226886_at | GFPT1 |
| 227039_at | AKAP13 |
| 227384_s_at | LINC00623 /// LOC727820 /// LOC728875 |
| 227443_at | LURAP1L |
| 227527_at | KMT2D |
| 227556_at | NME7 |
| 227616_at | BCL9L |
| 228051_at | KIAA1244 |
| 228232_s_at | VSIG2 |
| 228912_at | VIL1 |
| 228933_at | NHS |
| 229256_at | PGM2L1 |
| 229435_at | GLIS3 |
| 230271_at | ONECUT2 |
| 231628_s_at | SERPINB6 |
| 231823_s_at | SH3PXD2B |
| 231832_at | GALNT4 /// POC1B-GALNT4 |
| 232615_at | --- |
| 232889_at | --- |
| 232914_s_at | SYTL2 |
| 234675_x_at | --- |
| 234723_x_at | --- |
| 237475_x_at | CCDC152 |
| 238632_at | LOC100505946 |
| 239911_at | ONECUT2 |
| 37028_at | PPP1R15A |
| 39248_at | AQP3 |
| AFFX-BioB-M_at | --- |
| AFFX-HSAC07/X00351_5_at | ACTB |
| AFFX-HSAC07/X00351_M_at | ACTB |
| AFFX-M27830_5_at | --- |
| AFFX-M27830_M_at | --- |
| AFFX-r2-Ec-bioB-3_at | --- |
| AFFX-r2-Ec-bioB-M_at | --- |

## Supplementary Table 6. ρ^0^n_vivo Down-regulated signature.

The 161 probe sets down-regulated in xenografts of HT-29 ρ^0^n cells compared with HT-29 ρ^0^n cells in culture.

| **Probe.ID** | **NAME** |
| --- | --- |
| 1552680_a_at | CASC5 |
| 1553613_s_at | FOXC1 |
| 1554433_a_at | ZNF146 |
| 1554997_a_at | PTGS2 |
| 1555274_a_at | EPT1 |
| 1555910_at | PTCD2 |
| 1556551_s_at | SLC39A6 |
| 1558014_s_at | FAR1 |
| 1558093_s_at | MATR3 /// SNHG4 |
| 1558152_at | LOC100131262 |
| 201043_s_at | ANP32A |
| 201297_s_at | MOB1A |
| 201299_s_at | MOB1A |
| 201324_at | EMP1 |
| 201325_s_at | EMP1 |
| 201436_at | EIF4E |
| 201476_s_at | RRM1 |
| 201512_s_at | TOMM70A |
| 201831_s_at | USO1 |
| 201872_s_at | ABCE1 |
| 202013_s_at | EXT2 |
| 202053_s_at | ALDH3A2 |
| 202069_s_at | IDH3A |
| 202118_s_at | CPNE3 |
| 202536_at | CHMP2B |
| 202671_s_at | PDXK |
| 202886_s_at | PPP2R1B |
| 202912_at | ADM |
| 203065_s_at | CAV1 |
| 203105_s_at | DNM1L |
| 203180_at | ALDH1A3 |
| 203209_at | RFC5 |
| 203210_s_at | RFC5 |
| 203225_s_at | RFK |
| 203323_at | CAV2 |
| 203324_s_at | CAV2 |
| 203328_x_at | IDE |
| 203910_at | ARHGAP29 |
| 203967_at | CDC6 |
| 203968_s_at | CDC6 |
| 203971_at | SLC31A1 |
| 204025_s_at | PDCD2 |
| 204059_s_at | ME1 |
| 204120_s_at | ADK |
| 204493_at | BID |
| 204532_x_at | UGT1A1 /// UGT1A10 /// UGT1A4 /// UGT1A6 /// UGT1A8 /// UGT1A9 |
| 204716_at | CCDC6 |
| 204748_at | PTGS2 |
| 205047_s_at | ASNS |
| 205048_s_at | PSPH |
| 205194_at | PSPH |
| 205321_at | EIF2S3 |
| 205463_s_at | PDGFA |
| 205543_at | HSPA4L |
| 206094_x_at | UGT1A1 /// UGT1A10 /// UGT1A3 /// UGT1A4 /// UGT1A5 /// UGT1A6 /// UGT1A7 /// UGT1A8 /// UGT1A9 |
| 206205_at | MPHOSPH9 |
| 206561_s_at | AKR1B10 |
| 206737_at | WNT11 |
| 207126_x_at | UGT1A1 /// UGT1A10 /// UGT1A4 /// UGT1A6 /// UGT1A8 /// UGT1A9 |
| 207332_s_at | TFRC |
| 208209_s_at | C4BPB |
| 208290_s_at | EIF5 |
| 208510_s_at | PPARG |
| 208596_s_at | UGT1A1 /// UGT1A10 /// UGT1A3 /// UGT1A4 /// UGT1A5 /// UGT1A6 /// UGT1A7 /// UGT1A8 /// UGT1A9 |
| 208691_at | TFRC |
| 208708_x_at | EIF5 |
| 208839_s_at | CAND1 |
| 208853_s_at | CANX |
| 209513_s_at | HSDL2 |
| 209520_s_at | NCBP1 |
| 209773_s_at | RRM2 |
| 210076_x_at | SERBP1 |
| 211015_s_at | HSPA4 |
| 211016_x_at | HSPA4 |
| 211212_s_at | ORC5 |
| 212097_at | CAV1 |
| 212107_s_at | DHX9 |
| 212398_at | RDX |
| 212529_at | LSM12 |
| 212570_at | ENDOD1 |
| 212720_at | PAPOLA |
| 213286_at | ZFR |
| 213379_at | COQ2 |
| 213734_at | RFC5 |
| 213899_at | METAP2 |
| 214007_s_at | TWF1 |
| 214205_x_at | GLRX3 |
| 214316_x_at | CTC-425F1.4 /// OTTHUMG00000180575 |
| 214649_s_at | MTMR2 |
| 214697_s_at | PTBP3 |
| 214934_at | ATP9B |
| 215125_s_at | UGT1A1 /// UGT1A10 /// UGT1A3 /// UGT1A4 /// UGT1A5 /// UGT1A6 /// UGT1A7 /// UGT1A8 /// UGT1A9 |
| 215207_x_at | NUS1 /// NUS1P3 |
| 216841_s_at | LOC100129518 /// SOD2 |
| 217127_at | CTH |
| 217496_s_at | IDE |
| 217834_s_at | SYNCRIP |
| 217878_s_at | CDC27 |
| 218018_at | PDXK |
| 218073_s_at | NDC1 |
| 218100_s_at | IFT57 |
| 218392_x_at | SFXN1 |
| 218737_at | SBNO1 |
| 218772_x_at | TMEM38B |
| 218817_at | SPCS3 |
| 219014_at | PLAC8 |
| 219201_s_at | TWSG1 |
| 219320_at | MYO19 |
| 219508_at | GCNT3 |
| 220060_s_at | PARPBP |
| 220238_s_at | KLHL7 |
| 220239_at | KLHL7 |
| 220892_s_at | PSAT1 |
| 221027_s_at | PLA2G12A |
| 221059_s_at | COTL1 |
| 221305_s_at | UGT1A8 /// UGT1A9 |
| 221521_s_at | GINS2 |
| 221987_s_at | TSR1 |
| 222037_at | MCM4 |
| 222499_at | MRPS16 |
| 222519_s_at | IFT57 |
| 222600_s_at | UBA6 |
| 222646_s_at | ERO1L |
| 222654_at | IMPAD1 |
| 222781_s_at | C9orf40 |
| 223062_s_at | PSAT1 |
| 223308_s_at | WDR5 |
| 223839_s_at | --- |
| 224461_s_at | AIFM2 |
| 224744_at | IMPAD1 |
| 225366_at | PGM2 |
| 225686_at | SKA2 |
| 225688_s_at | PHLDB2 |
| 225736_at | FBXO22 |
| 226661_at | CDCA2 |
| 226826_at | LSM11 |
| 226907_at | PPP1R14C |
| 227211_at | PHF19 |
| 227741_at | PTPLB |
| 228204_at | PSMB4 |
| 228868_x_at | CDT1 |
| 228986_at | OSBPL8 |
| 229083_at | HNRNPA0 |
| 229181_s_at | HAUS2 |
| 229795_at | --- |
| 230250_at | PTPRB |
| 230265_at | --- |
| 230306_at | VPS26B |
| 231837_at | USP28 |
| 231995_at | CAAP1 |
| 233208_x_at | CPSF2 |
| 233827_s_at | SUPT16H |
| 234304_s_at | IPO11 /// IPO11-LRRC70 |
| 234915_s_at | DENR |
| 235134_at | LOC100996578 |
| 235694_at | TCFL5 |
| 236957_at | CDCA2 |
| 238034_at | CANX |
| 238419_at | PHLDB2 |
| 238623_at | --- |
| 242787_at | --- |

## Supplementary Table 7. Enrichment result, up-regulated c2 signatures in HT-29 ρ^0^x cells compared with in HT-29 ρ^0^n cells.

The detail results of GSEA (TOP50) shown in Figure 3a and Supplementary Figure 2. HT-29 ρ^0^x cells were assigned to class A, and HT-29 ρ^0^n cells were assigned to class B in GSEA software [2, 3].

| **RANK** | **NAME** | **NOMp-val** | **FDRq-val** |
| --- | --- | --- | --- |
| 1 | NIKOLSKY_BREAST_CANCER_17Q21_Q25_AMPLICON | 0.000 | 0.000 |
| 2 | WINTER_HYPOXIA_METAGENE | 0.000 | 0.000 |
| 3 | CHARAFE_BREAST_CANCER_LUMINAL_VS_MESENCHYMAL_DN | 0.000 | 0.000 |
| 4 | LASTOWSKA_NEUROBLASTOMA_COPY_NUMBER_UP | 0.000 | 0.000 |
| 5 | VECCHI_GASTRIC_CANCER_ADVANCED_VS_EARLY_UP | 0.000 | 0.000 |
| 6 | GOZGIT_ESR1_TARGETS_DN | 0.000 | 0.000 |
| 7 | AMIT_SERUM_RESPONSE_60_MCF10A | 0.000 | 0.000 |
| 8 | SCHUETZ_BREAST_CANCER_DUCTAL_INVASIVE_UP | 0.000 | 0.000 |
| 9 | TAKEDA_TARGETS_OF_NUP98_HOXA9_FUSION_8D_UP | 0.000 | 0.000 |
| 10 | PASINI_SUZ12_TARGETS_DN | 0.000 | 0.000 |
| 11 | DUTERTRE_ESTRADIOL_RESPONSE_24HR_DN | 0.000 | 0.000 |
| 12 | POTTI_TOPOTECAN_SENSITIVITY | 0.000 | 0.000 |
| 13 | HUPER_BREAST_BASAL_VS_LUMINAL_DN | 0.000 | 0.000 |
| 14 | TAKEDA_TARGETS_OF_NUP98_HOXA9_FUSION_10D_UP | 0.000 | 0.000 |
| 15 | BOYAULT_LIVER_CANCER_SUBCLASS_G3_UP | 0.000 | 0.000 |
| 16 | MIYAGAWA_TARGETS_OF_EWSR1_ETS_FUSIONS_DN | 0.000 | 0.000 |
| 17 | NIKOLSKY_BREAST_CANCER_17Q11_Q21_AMPLICON | 0.000 | 0.000 |
| 18 | ONDER_CDH1_TARGETS_2_UP | 0.000 | 0.000 |
| 19 | CHARAFE_BREAST_CANCER_LUMINAL_VS_BASAL_DN | 0.000 | 0.000 |
| 20 | BRUECKNER_TARGETS_OF_MIRLET7A3_DN | 0.000 | 0.000 |
| 21 | BAELDE_DIABETIC_NEPHROPATHY_DN | 0.000 | 0.000 |
| 22 | LINDGREN_BLADDER_CANCER_CLUSTER_2B | 0.000 | 0.000 |
| 23 | WINZEN_DEGRADED_VIA_KHSRP | 0.000 | 0.000 |
| 24 | ELVIDGE_HYPOXIA_BY_DMOG_UP | 0.000 | 0.000 |
| 25 | KOBAYASHI_EGFR_SIGNALING_24HR_DN | 0.000 | 0.000 |
| 26 | SWEET_KRAS_TARGETS_UP | 0.000 | 0.001 |
| 27 | ELVIDGE_HYPOXIA_UP | 0.000 | 0.001 |
| 28 | ROZANOV_MMP14_TARGETS_UP | 0.000 | 0.001 |
| 29 | AMIT_EGF_RESPONSE_60_MCF10A | 0.000 | 0.001 |
| 30 | CHIANG_LIVER_CANCER_SUBCLASS_PROLIFERATION_UP | 0.000 | 0.001 |
| 31 | CHIANG_LIVER_CANCER_SUBCLASS_CTNNB1_DN | 0.000 | 0.001 |
| 32 | NAKAMURA_ADIPOGENESIS_EARLY_DN | 0.000 | 0.001 |
| 33 | WANG_SMARCE1_TARGETS_UP | 0.000 | 0.001 |
| 34 | GRAHAM_CML_DIVIDING_VS_NORMAL_QUIESCENT_UP | 0.000 | 0.001 |
| 35 | MARKEY_RB1_ACUTE_LOF_UP | 0.000 | 0.001 |
| 36 | LU_AGING_BRAIN_UP | 0.000 | 0.001 |
| 37 | SOTIRIOU_BREAST_CANCER_GRADE_1_VS_3_UP | 0.000 | 0.001 |
| 38 | TONKS_TARGETS_OF_RUNX1_RUNX1T1_FUSION_HSC_UP | 0.000 | 0.001 |
| 39 | DANG_REGULATED_BY_MYC_DN | 0.000 | 0.001 |
| 40 | KATSANOU_ELAVL1_TARGETS_UP | 0.000 | 0.001 |
| 41 | THUM_SYSTOLIC_HEART_FAILURE_UP | 0.000 | 0.001 |
| 42 | ZWANG_CLASS_1_TRANSIENTLY_INDUCED_BY_EGF | 0.000 | 0.001 |
| 43 | BENPORATH_PROLIFERATION | 0.000 | 0.001 |
| 44 | SMID_BREAST_CANCER_BASAL_DN | 0.000 | 0.001 |
| 45 | JOHNSTONE_PARVB_TARGETS_3_UP | 0.000 | 0.001 |
| 46 | GRAHAM_CML_DIVIDING_VS_NORMAL_QUIESCENT_DN | 0.000 | 0.001 |
| 47 | BOYLAN_MULTIPLE_MYELOMA_C_D_DN | 0.000 | 0.001 |
| 48 | LIU_PROSTATE_CANCER_DN | 0.000 | 0.001 |
| 49 | CHICAS_RB1_TARGETS_CONFLUENT | 0.000 | 0.001 |
| 50 | BLUM_RESPONSE_TO_SALIRASIB_DN | 0.000 | 0.001 |

## Supplementary Table 8. Enrichment result, up-regulated c2 signatures in HT-29 ρ^0^n cells compared with in HT-29 ρ^0^x cells.

The detail results of GSEA (TOP50), shown in Figure 3a and Supplementary Figure 2. HT-29 ρ^0^n cells were assigned to class A and HT-29 ρ^0^x cells were assigned to class B in GSEA software.

| **RANK** | **NAME** | **NOMp-val** | **FDRq-val** |
| --- | --- | --- | --- |
| 1 | NIKOLSKY_BREAST_CANCER_7Q21_Q22_AMPLICON | 0.000 | 0.000 |
| 2 | LINDGREN_BLADDER_CANCER_WITH_LOH_IN_CHR9Q | 0.000 | 0.000 |
| 3 | KRIGE_AMINO_ACID_DEPRIVATION | 0.000 | 0.000 |
| 4 | TSUNODA_CISPLATIN_RESISTANCE_DN | 0.000 | 0.000 |
| 5 | SENESE_HDAC2_TARGETS_DN | 0.000 | 0.000 |
| 6 | JAEGER_METASTASIS_DN | 0.000 | 0.000 |
| 7 | ZHAN_MULTIPLE_MYELOMA_CD1_VS_CD2_UP | 0.000 | 0.000 |
| 8 | HUPER_BREAST_BASAL_VS_LUMINAL_UP | 0.000 | 0.000 |
| 9 | HELLER_SILENCED_BY_METHYLATION_DN | 0.000 | 0.000 |
| 10 | LIANG_SILENCED_BY_METHYLATION_2 | 0.000 | 0.000 |
| 11 | WU_CELL_MIGRATION | 0.000 | 0.000 |
| 12 | PACHER_TARGETS_OF_IGF1_AND_IGF2_UP | 0.000 | 0.000 |
| 13 | HOOI_ST7_TARGETS_UP | 0.000 | 0.000 |
| 14 | REACTOME_3_UTR_MEDIATED_TRANSLATIONAL_REGULATION | 0.000 | 0.000 |
| 15 | REACTOME_PEPTIDE_CHAIN_ELONGATION | 0.000 | 0.001 |
| 16 | ROVERSI_GLIOMA_COPY_NUMBER_UP | 0.000 | 0.001 |
| 17 | SMID_BREAST_CANCER_LUMINAL_B_DN | 0.000 | 0.001 |
| 18 | KAN_RESPONSE_TO_ARSENIC_TRIOXIDE | 0.000 | 0.001 |
| 19 | KEGG_RIBOSOME | 0.000 | 0.001 |
| 20 | REACTOME_RESPIRATORY_ELECTRON_TRANSPORT_ATP_SYNTHESIS_BY_CHEMIOSMOTIC_COUPLING_AND_HEAT_PRODUCTION_BY_UNCOUPLING_PROTEINS_ | 0.000 | 0.001 |
| 21 | REACTOME_NONSENSE_MEDIATED_DECAY_ENHANCED_BY_THE_EXON_JUNCTION_COMPLEX | 0.000 | 0.001 |
| 22 | STOSSI_RESPONSE_TO_ESTRADIOL | 0.000 | 0.001 |
| 23 | SENESE_HDAC1_AND_HDAC2_TARGETS_DN | 0.000 | 0.001 |
| 24 | VECCHI_GASTRIC_CANCER_ADVANCED_VS_EARLY_DN | 0.000 | 0.001 |
| 25 | WOOD_EBV_EBNA1_TARGETS_DN | 0.000 | 0.002 |
| 26 | CHNG_MULTIPLE_MYELOMA_HYPERPLOID_UP | 0.000 | 0.002 |
| 27 | REACTOME_RESPIRATORY_ELECTRON_TRANSPORT | 0.000 | 0.002 |
| 28 | TAKEDA_TARGETS_OF_NUP98_HOXA9_FUSION_3D_UP | 0.000 | 0.002 |
| 29 | IGARASHI_ATF4_TARGETS_DN | 0.000 | 0.003 |
| 30 | MCLACHLAN_DENTAL_CARIES_DN | 0.000 | 0.003 |
| 31 | RIGGI_EWING_SARCOMA_PROGENITOR_UP | 0.000 | 0.003 |
| 32 | NIKOLSKY_BREAST_CANCER_7P22_AMPLICON | 0.000 | 0.004 |
| 33 | LI_PROSTATE_CANCER_EPIGENETIC | 0.000 | 0.004 |
| 34 | BOQUEST_STEM_CELL_UP | 0.000 | 0.004 |
| 35 | COLDREN_GEFITINIB_RESISTANCE_UP | 0.000 | 0.005 |
| 36 | ZHAN_MULTIPLE_MYELOMA_CD1_UP | 0.000 | 0.005 |
| 37 | ONDER_CDH1_TARGETS_3_DN | 0.000 | 0.006 |
| 38 | ONDER_CDH1_TARGETS_1_UP | 0.000 | 0.006 |
| 39 | BHAT_ESR1_TARGETS_VIA_AKT1_UP | 0.000 | 0.006 |
| 40 | CHARAFE_BREAST_CANCER_LUMINAL_VS_MESENCHYMAL_UP | 0.000 | 0.006 |
| 41 | KOYAMA_SEMA3B_TARGETS_UP | 0.000 | 0.006 |
| 42 | KEGG_VALINE_LEUCINE_AND_ISOLEUCINE_DEGRADATION | 0.000 | 0.006 |
| 43 | SMID_BREAST_CANCER_RELAPSE_IN_BONE_DN | 0.000 | 0.006 |
| 44 | MOOTHA_VOXPHOS | 0.000 | 0.006 |
| 45 | GARY_CD5_TARGETS_UP | 0.000 | 0.006 |
| 46 | ENGELMANN_CANCER_PROGENITORS_DN | 0.000 | 0.007 |
| 47 | MOHANKUMAR_TLX1_TARGETS_DN | 0.000 | 0.007 |
| 48 | MCLACHLAN_DENTAL_CARIES_UP | 0.000 | 0.007 |
| 49 | REACTOME_BRANCHED_CHAIN_AMINO_ACID_CATABOLISM | 0.000 | 0.007 |
| 50 | ISSAEVA_MLL2_TARGETS | 0.000 | 0.007 |

## Supplementary Table 10. Xenograft up-regulated signature.

The 496 probe sets, which were up-regulated in xenografts of HT-29 Pt or HT-29 ρ^0^n cells, compared with HT-29 Pt or HT-29 ρ^0^n cells in culture, respectively.

| **Probe.ID** | **NAME** |
| --- | --- |
| 1552621_at | POLR2J2 /// POLR2J3 /// UPK3BL |
| 1553185_at | RASEF |
| 1553186_x_at | RASEF |
| 1553538_s_at | COX1 |
| 1553551_s_at | ND2 |
| 1553567_s_at | ATP6 |
| 1553569_at | COX2 /// OAF /// TLE1 |
| 1553570_x_at | COX2 /// OAF /// TLE1 |
| 1553575_at | ND6 |
| 1553611_s_at | KLHL35 |
| 1554600_s_at | LMNA |
| 1555653_at | --- |
| 1555730_a_at | CFL1 |
| 1555745_a_at | LYZ |
| 1555832_s_at | KLF6 |
| 1555851_s_at | SEPW1 |
| 1557987_at | --- |
| 1558678_s_at | MALAT1 |
| 1566887_x_at | --- |
| 1569454_a_at | LOC283352 |
| 200001_at | CAPNS1 |
| 200601_at | ACTN4 |
| 200606_at | DSP |
| 200632_s_at | NDRG1 |
| 200634_at | PFN1 |
| 200645_at | GABARAP |
| 200660_at | S100A11 |
| 200696_s_at | GSN |
| 200742_s_at | TPP1 |
| 200771_at | LAMC1 |
| 200852_x_at | GNB2 |
| 200859_x_at | FLNA |
| 200887_s_at | STAT1 |
| 200904_at | HLA-E |
| 200905_x_at | HLA-E |
| 200920_s_at | BTG1 |
| 200921_s_at | BTG1 |
| 200923_at | LGALS3BP |
| 200964_at | UBA1 |
| 201008_s_at | LOC101060503 /// TXNIP |
| 201009_s_at | LOC101060503 /// TXNIP |
| 201010_s_at | LOC101060503 /// TXNIP |
| 201040_at | GNAI2 |
| 201041_s_at | DUSP1 |
| 201105_at | LGALS1 |
| 201162_at | IGFBP7 |
| 201188_s_at | ITPR3 |
| 201194_at | SEPW1 |
| 201234_at | ILK |
| 201236_s_at | BTG2 |
| 201242_s_at | ATP1B1 |
| 201243_s_at | ATP1B1 |
| 201310_s_at | NREP |
| 201311_s_at | SH3BGRL |
| 201312_s_at | SH3BGRL |
| 201313_at | ENO2 |
| 201315_x_at | IFITM2 |
| 201369_s_at | ZFP36L2 |
| 201416_at | SOX4 |
| 201418_s_at | SOX4 |
| 201426_s_at | VIM |
| 201427_s_at | SEPP1 |
| 201464_x_at | JUN |
| 201465_s_at | JUN |
| 201506_at | TGFBI |
| 201564_s_at | FSCN1 |
| 201601_x_at | IFITM1 /// IFITM2 |
| 201641_at | BST2 |
| 201655_s_at | HSPG2 |
| 201666_at | TIMP1 |
| 201669_s_at | MARCKS |
| 201670_s_at | MARCKS |
| 201694_s_at | EGR1 |
| 201841_s_at | HSPB1 |
| 201850_at | CAPG |
| 201876_at | PON2 |
| 201884_at | CEACAM5 |
| 201908_at | DVL3 |
| 201939_at | PLK2 |
| 202074_s_at | OPTN |
| 202102_s_at | BRD4 |
| 202237_at | NNMT |
| 202238_s_at | NNMT |
| 202411_at | IFI27 |
| 202430_s_at | PLSCR1 |
| 202446_s_at | PLSCR1 |
| 202489_s_at | FXYD3 |
| 202672_s_at | ATF3 |
| 202761_s_at | SYNE2 |
| 202826_at | SPINT1 |
| 202833_s_at | SERPINA1 |
| 202859_x_at | IL8 |
| 202869_at | OAS1 |
| 203020_at | RABGAP1L |
| 203080_s_at | BAZ2B |
| 203139_at | DAPK1 |
| 203153_at | IFIT1 |
| 203186_s_at | S100A4 |
| 203304_at | BAMBI |
| 203394_s_at | HES1 |
| 203455_s_at | SAT1 |
| 203595_s_at | IFIT5 |
| 203638_s_at | FGFR2 |
| 203640_at | MBNL2 |
| 203688_at | PKD2 |
| 203691_at | PI3 |
| 203725_at | GADD45A |
| 203726_s_at | LAMA3 |
| 203752_s_at | JUND |
| 203753_at | TCF4 |
| 203799_at | CD302 /// LY75-CD302 |
| 203882_at | IRF9 |
| 203911_at | RAP1GAP |
| 203939_at | NT5E |
| 203953_s_at | CLDN3 |
| 203954_x_at | CLDN3 |
| 203961_at | NEBL |
| 204070_at | RARRES3 |
| 204073_s_at | MYRF |
| 204235_s_at | GULP1 |
| 204237_at | GULP1 |
| 204259_at | MMP7 |
| 204272_at | LGALS4 |
| 204306_s_at | CD151 |
| 204379_s_at | FGFR3 |
| 204415_at | IFI6 |
| 204538_x_at | NPIPA1 |
| 204622_x_at | NR4A2 |
| 204713_s_at | F5 |
| 204714_s_at | F5 |
| 204805_s_at | H1FX |
| 204806_x_at | HLA-F |
| 204972_at | OAS2 |
| 204990_s_at | ITGB4 |
| 205044_at | GABRP |
| 205137_x_at | USH1C |
| 205157_s_at | JUP /// KRT17 |
| 205483_s_at | ISG15 |
| 205506_at | VIL1 |
| 205513_at | TCN1 |
| 205552_s_at | OAS1 |
| 205559_s_at | PCSK5 |
| 205660_at | OASL |
| 205927_s_at | CTSE |
| 205969_at | AADAC |
| 206239_s_at | SPINK1 |
| 206323_x_at | OPHN1 |
| 206560_s_at | MIA |
| 206632_s_at | APOBEC3B |
| 206858_s_at | HOXC6 |
| 207011_s_at | PTK7 |
| 207173_x_at | CDH11 |
| 207214_at | SPINK4 |
| 207365_x_at | USP34 |
| 207435_s_at | SRRM2 |
| 207436_x_at | --- |
| 207730_x_at | --- |
| 207808_s_at | PROS1 |
| 207847_s_at | MUC1 |
| 208082_x_at | MKRN4P /// MKRN4P |
| 208228_s_at | FGFR2 |
| 208246_x_at | OTTHUMG00000176438 /// RP11-403P17.4 |
| 208436_s_at | IRF7 |
| 208540_x_at | S100A11P1 /// S100A11P1 |
| 208610_s_at | SRRM2 |
| 208637_x_at | ACTN1 |
| 208723_at | USP11 |
| 208729_x_at | HLA-B |
| 208749_x_at | FLOT1 |
| 208798_x_at | GOLGA8A |
| 208812_x_at | HLA-C |
| 208829_at | TAPBP |
| 208960_s_at | KLF6 |
| 208961_s_at | KLF6 |
| 208997_s_at | UCP2 |
| 209140_x_at | HLA-B |
| 209173_at | AGR2 |
| 209189_at | FOS |
| 209201_x_at | CXCR4 |
| 209212_s_at | KLF5 |
| 209216_at | WDR45 |
| 209270_at | LAMB3 |
| 209386_at | TM4SF1 |
| 209387_s_at | TM4SF1 |
| 209403_at | LOC100510707 /// LOC101060287 /// LOC101060303 /// LOC101060321 /// LOC101060351 /// LOC101060367 /// LOC101060376 /// LOC101060389 /// LOC101060403 /// LOC101060421 /// LOC101060440 /// LOC101060471 /// LOC101060489 /// LOC101060506 /// LOC101060522 /// TBC1D3 /// TBC1D3C /// TBC1D3F /// TBC1D3G /// TBC1D3H |
| 209417_s_at | IFI35 |
| 209498_at | CEACAM1 |
| 209504_s_at | PLEKHB1 |
| 209546_s_at | APOL1 |
| 209641_s_at | ABCC3 |
| 209761_s_at | SP110 |
| 209911_x_at | HIST1H2BD |
| 209969_s_at | STAT1 |
| 210046_s_at | IDH2 |
| 210105_s_at | FYN |
| 210117_at | SPAG1 |
| 210218_s_at | SP100 |
| 210230_at | --- |
| 210445_at | FABP6 |
| 210512_s_at | VEGFA |
| 210514_x_at | HLA-G |
| 210592_s_at | SAT1 |
| 210679_x_at | --- |
| 210797_s_at | OASL |
| 210827_s_at | ELF3 |
| 210830_s_at | PON2 |
| 210978_s_at | TAGLN2 |
| 211074_at | --- |
| 211160_x_at | ACTN1 |
| 211184_s_at | USH1C |
| 211429_s_at | SERPINA1 |
| 211528_x_at | HLA-G |
| 211529_x_at | HLA-G |
| 211530_x_at | HLA-G |
| 211600_at | PTPRO |
| 211799_x_at | HLA-C |
| 211911_x_at | HLA-B |
| 211919_s_at | CXCR4 |
| 211934_x_at | GANAB |
| 211950_at | UBR4 |
| 211964_at | COL4A2 |
| 211986_at | AHNAK |
| 211996_s_at | LOC101060455 /// LOC101060564 /// LOC595101 /// LOC613037 /// NPIPB11 /// NPIPB3 /// NPIPB4 /// NPIPB5 |
| 212076_at | KMT2A |
| 212077_at | CALD1 |
| 212080_at | KMT2A |
| 212099_at | RHOB |
| 212151_at | PBX1 |
| 212203_x_at | IFITM3 |
| 212224_at | ALDH1A1 |
| 212236_x_at | JUP /// KRT17 |
| 212254_s_at | DST |
| 212263_at | QKI |
| 212338_at | MYO1D |
| 212386_at | TCF4 |
| 212472_at | MICAL2 |
| 213142_x_at | GSAP |
| 213158_at | OTTHUMG00000175755 /// RP11-553L6.5 |
| 213294_at | EIF2AK2 |
| 213359_at | HNRNPD |
| 213397_x_at | RNASE4 |
| 213517_at | PCBP2 |
| 213605_s_at | --- |
| 213668_s_at | SOX4 |
| 213693_s_at | MUC1 |
| 213695_at | PON3 |
| 213746_s_at | FLNA |
| 213932_x_at | HLA-A |
| 213988_s_at | SAT1 |
| 214022_s_at | IFITM1 |
| 214035_x_at | LOC399491 |
| 214041_x_at | RPL37A |
| 214218_s_at | XIST |
| 214234_s_at | CYP3A5 |
| 214290_s_at | HIST2H2AA3 /// HIST2H2AA4 |
| 214453_s_at | IFI44 |
| 214459_x_at | HLA-C |
| 214594_x_at | ATP8B1 |
| 214693_x_at | LOC101059961 /// LOC101060202 /// LOC101060362 /// LOC101060684 /// NBPF10 /// NBPF12 /// NBPF9 |
| 214707_x_at | ALMS1 |
| 214715_x_at | ZNF160 |
| 214722_at | NOTCH2NL |
| 214752_x_at | FLNA |
| 214774_x_at | TOX3 |
| 214870_x_at | LOC101060412 /// NPIPA1 /// NPIPA2 /// NPIPA3 /// NPIPA5 /// NPIPA7 /// NPIPA8 /// PKD1P1 |
| 214902_x_at | --- |
| 214989_x_at | --- |
| 215016_x_at | DST |
| 215034_s_at | TM4SF1 |
| 215071_s_at | HIST1H2AC |
| 215076_s_at | COL3A1 |
| 215108_x_at | TOX3 |
| 215127_s_at | RBMS1 |
| 215179_x_at | PGF |
| 215313_x_at | HLA-A |
| 215383_x_at | SPG21 |
| 215504_x_at | ANKRD10-IT1 |
| 215553_x_at | --- |
| 215588_x_at | RIOK3 |
| 215600_x_at | FBXW12 |
| 215978_x_at | ZNF721 |
| 216032_s_at | ERGIC3 |
| 216153_x_at | RECK |
| 216187_x_at | --- |
| 216231_s_at | B2M |
| 216405_at | --- |
| 216524_x_at | --- |
| 216526_x_at | HLA-C |
| 216563_at | ANKRD12 |
| 216565_x_at | OTTHUMG00000003319 /// RP4-781L3.1 |
| 216623_x_at | TOX3 |
| 216836_s_at | ERBB2 |
| 217028_at | CXCR4 |
| 217436_x_at | HLA-J |
| 217456_x_at | HLA-E |
| 217523_at | CD44 |
| 217586_x_at | --- |
| 217673_x_at | GNAS |
| 217683_at | HBE1 |
| 217703_x_at | --- |
| 217715_x_at | --- |
| 217783_s_at | YPEL5 |
| 217867_x_at | BACE2 |
| 218084_x_at | FXYD5 |
| 218164_at | SPATA20 |
| 218309_at | CAMK2N1 |
| 218322_s_at | ACSL5 |
| 218400_at | OAS3 |
| 218471_s_at | BBS1 |
| 218831_s_at | FCGRT |
| 218858_at | DEPTOR |
| 218872_at | TESC |
| 218962_s_at | TMEM168 |
| 218986_s_at | DDX60 |
| 219209_at | IFIH1 |
| 219352_at | HERC6 |
| 219630_at | PDZK1IP1 |
| 219878_s_at | KLF13 |
| 220755_s_at | C6orf48 |
| 220796_x_at | SLC35E1 |
| 221004_s_at | ITM2C |
| 221501_x_at | NPIPA2 /// NPIPA3 /// NPIPA5 /// PKD1P1 |
| 221577_x_at | GDF15 |
| 221728_x_at | XIST |
| 221748_s_at | TNS1 |
| 221764_at | R3HDM4 |
| 221768_at | LOC100996496 /// SFPQ |
| 221816_s_at | PHF11 |
| 221875_x_at | HLA-F |
| 221899_at | N4BP2L2 |
| 222067_x_at | HIST1H2BD |
| 222150_s_at | GSAP |
| 222175_s_at | MED15 |
| 222408_s_at | YPEL5 |
| 222728_s_at | MIR1304 /// SNORA1 /// SNORA18 /// SNORA32 /// SNORA40 /// SNORA8 /// SNORD5 /// TAF1D |
| 222803_at | PRTFDC1 |
| 222986_s_at | SHISA5 |
| 223044_at | SLC40A1 |
| 223103_at | STARD10 |
| 223125_s_at | C1orf21 |
| 223218_s_at | NFKBIZ |
| 223220_s_at | PARP9 |
| 223251_s_at | ANKRD10 |
| 223451_s_at | CKLF |
| 223494_at | MGEA5 |
| 223497_at | FAM135A |
| 223662_x_at | DDX59 |
| 223939_at | SUCNR1 |
| 223940_x_at | MALAT1 |
| 224159_x_at | TRIM4 |
| 224344_at | COX6A1 |
| 224372_at | C10orf99 /// CCDC104 /// ND4 |
| 224373_s_at | C10orf99 /// CCDC104 /// HNRNPM /// ND4 |
| 224428_s_at | CDCA7 |
| 224549_x_at | --- |
| 224558_s_at | MALAT1 |
| 224559_at | MALAT1 |
| 224565_at | MIR612 /// NEAT1 |
| 224566_at | MIR612 /// NEAT1 |
| 224567_x_at | MALAT1 |
| 224588_at | XIST |
| 224606_at | KLF6 |
| 224667_x_at | ANAPC16 |
| 224701_at | PARP14 |
| 224741_x_at | GAS5 /// SNORD44 /// SNORD47 /// SNORD76 /// SNORD77 /// SNORD79 /// SNORD80 /// SNORD81 |
| 224841_x_at | GAS5 /// SNORD44 /// SNORD47 /// SNORD76 /// SNORD77 /// SNORD79 /// SNORD80 /// SNORD81 |
| 224915_x_at | ZFAS1 |
| 224917_at | MIR21 /// VMP1 |
| 224992_s_at | CMIP |
| 225033_at | ST3GAL1 |
| 225056_at | SIPA1L2 |
| 225093_at | UTRN |
| 225105_at | C12orf75 |
| 225155_at | SNHG5 /// SNORD50A /// SNORD50B |
| 225239_at | MIR612 /// NEAT1 |
| 225368_at | HIPK2 |
| 225415_at | DTX3L |
| 225496_s_at | SYTL2 |
| 225628_s_at | MLLT6 |
| 225636_at | STAT2 |
| 225698_at | EPB41L4A-AS1 |
| 225786_at | HNRNPU-AS1 |
| 225899_x_at | FLJ45340 /// FLJ45445 /// LOC100287894 /// LOC100996328 /// LOC100996502 /// LOC101059936 /// LOC101060494 /// LOC101060495 /// LOC729737 |
| 225929_s_at | RNF213 |
| 226123_at | CHD7 |
| 226145_s_at | FRAS1 |
| 226158_at | KLHL24 |
| 226227_x_at | ZFAS1 |
| 226275_at | MXD1 |
| 226403_at | TMC4 |
| 226446_at | HES6 |
| 226459_at | PIK3AP1 |
| 226535_at | ITGB6 |
| 226560_at | --- |
| 226575_at | ZNF462 |
| 226663_at | ANKRD10-IT1 |
| 226675_s_at | MALAT1 |
| 226757_at | IFIT2 |
| 226835_s_at | ZFAS1 |
| 226844_at | MOB3B |
| 226886_at | GFPT1 |
| 227020_at | YPEL2 |
| 227039_at | AKAP13 |
| 227129_x_at | FLJ45340 |
| 227223_at | RBM39 |
| 227383_at | LOC727820 |
| 227384_s_at | LINC00623 /// LOC727820 /// LOC728875 |
| 227443_at | LURAP1L |
| 227527_at | KMT2D |
| 227556_at | NME7 |
| 227616_at | BCL9L |
| 227667_at | CUEDC1 |
| 227671_at | XIST |
| 227891_s_at | TAF15 |
| 227996_at | FARP1 /// FARP1-IT1 |
| 228051_at | KIAA1244 |
| 228157_at | ZNF207 |
| 228232_s_at | VSIG2 |
| 228531_at | SAMD9 |
| 228912_at | VIL1 |
| 228933_at | NHS |
| 228999_at | CHD2 /// LOC100507217 |
| 229030_at | CAPN8 |
| 229256_at | PGM2L1 |
| 229434_at | --- |
| 229435_at | GLIS3 |
| 229450_at | IFIT3 |
| 229553_at | PGM2L1 |
| 229765_at | ZNF207 |
| 229966_at | EWSR1 |
| 230271_at | ONECUT2 |
| 231579_s_at | TIMP2 |
| 231628_s_at | SERPINB6 |
| 231735_s_at | LOC100996467 /// MALAT1 |
| 231770_x_at | WDPCP |
| 231823_s_at | SH3PXD2B |
| 231825_x_at | ATF7IP |
| 231832_at | GALNT4 /// POC1B-GALNT4 |
| 232174_at | --- |
| 232266_x_at | CDK13 |
| 232597_x_at | SCAF11 |
| 232615_at | --- |
| 232683_s_at | PARP6 |
| 232889_at | --- |
| 232914_s_at | SYTL2 |
| 233296_x_at | --- |
| 233319_x_at | --- |
| 233702_x_at | --- |
| 233775_x_at | LOC100289333 |
| 234675_x_at | --- |
| 234723_x_at | --- |
| 234725_s_at | SEMA4B |
| 234762_x_at | NLN |
| 234788_x_at | --- |
| 234987_at | SAMHD1 |
| 234989_at | MIR612 /// NEAT1 |
| 235028_at | --- |
| 235060_at | LOC100190986 /// LOC101060564 |
| 235419_at | --- |
| 235529_x_at | SAMHD1 |
| 235716_at | --- |
| 235763_at | SLC44A5 |
| 235879_at | --- |
| 235964_x_at | SAMHD1 |
| 237475_x_at | CCDC152 |
| 237768_x_at | --- |
| 238199_x_at | COX3 |
| 238558_at | --- |
| 238632_at | LOC100505946 |
| 238701_x_at | C11orf92 |
| 239332_at | LOC100506373 |
| 239748_x_at | OCIAD1 |
| 239911_at | ONECUT2 |
| 241681_at | --- |
| 241993_x_at | --- |
| 242121_at | --- |
| 242235_x_at | --- |
| 242669_at | UFM1 |
| 242961_x_at | DDX58 |
| 244197_x_at | --- |
| 32837_at | AGPAT2 |
| 36564_at | RNF19B |
| 36994_at | ATP6V0C |
| 37028_at | PPP1R15A |
| 39248_at | AQP3 |
| 41469_at | PI3 |
| 53720_at | C19orf66 |
| AFFX-BioB-M_at | --- |
| AFFX-HSAC07/X00351_5_at | ACTB |
| AFFX-HSAC07/X00351_M_at | ACTB |
| AFFX-HUMISGF3A/M97935_3_at | STAT1 |
| AFFX-M27830_5_at | --- |
| AFFX-M27830_M_at | --- |
| AFFX-r2-Ec-bioB-3_at | --- |
| AFFX-r2-Ec-bioB-5_at | --- |
| AFFX-r2-Ec-bioB-M_at | --- |

## Supplementary Table 11. Xenograft down-regulated signature.

The 303 probe sets, which were down-regulated in xenografts of HT-29 Pt or HT-29 ρ^0^n cells, compared with HT-29 Pt or HT-29 ρ^0^n cells in culture, respectively.

| **Probe.ID** | **NAME** |
| --- | --- |
| 1552680_a_at | CASC5 |
| 1553528_a_at | TAF5 |
| 1553613_s_at | FOXC1 |
| 1554101_a_at | TMTC4 |
| 1554390_s_at | ACTR2 |
| 1554414_a_at | OSGIN2 |
| 1554433_a_at | ZNF146 |
| 1554997_a_at | PTGS2 |
| 1555058_a_at | LPGAT1 |
| 1555225_at | C1orf43 |
| 1555274_a_at | EPT1 |
| 1555334_s_at | SLC30A5 |
| 1555383_a_at | POF1B |
| 1555910_at | PTCD2 |
| 1556551_s_at | SLC39A6 |
| 1558014_s_at | FAR1 |
| 1558093_s_at | MATR3 /// SNHG4 |
| 1558152_at | LOC100131262 |
| 1560916_a_at | DPY19L1 |
| 200727_s_at | ACTR2 |
| 200841_s_at | EPRS |
| 201043_s_at | ANP32A |
| 201211_s_at | DDX3X |
| 201297_s_at | MOB1A |
| 201299_s_at | MOB1A |
| 201324_at | EMP1 |
| 201325_s_at | EMP1 |
| 201436_at | EIF4E |
| 201476_s_at | RRM1 |
| 201512_s_at | TOMM70A |
| 201562_s_at | SORD |
| 201569_s_at | SAMM50 |
| 201626_at | INSIG1 |
| 201627_s_at | INSIG1 |
| 201661_s_at | ACSL3 |
| 201831_s_at | USO1 |
| 201872_s_at | ABCE1 |
| 201963_at | ACSL1 |
| 202013_s_at | EXT2 |
| 202053_s_at | ALDH3A2 |
| 202054_s_at | ALDH3A2 |
| 202069_s_at | IDH3A |
| 202118_s_at | CPNE3 |
| 202170_s_at | AASDHPPT |
| 202435_s_at | CYP1B1 |
| 202436_s_at | CYP1B1 |
| 202437_s_at | CYP1B1 |
| 202536_at | CHMP2B |
| 202671_s_at | PDXK |
| 202886_s_at | PPP2R1B |
| 202912_at | ADM |
| 203032_s_at | FH |
| 203033_x_at | FH |
| 203065_s_at | CAV1 |
| 203105_s_at | DNM1L |
| 203180_at | ALDH1A3 |
| 203209_at | RFC5 |
| 203210_s_at | RFC5 |
| 203211_s_at | MTMR2 |
| 203225_s_at | RFK |
| 203283_s_at | HS2ST1 |
| 203323_at | CAV2 |
| 203324_s_at | CAV2 |
| 203328_x_at | IDE |
| 203339_at | SLC25A12 |
| 203343_at | UGDH |
| 203418_at | CCNA2 |
| 203474_at | IQGAP2 |
| 203899_s_at | CRCP |
| 203910_at | ARHGAP29 |
| 203967_at | CDC6 |
| 203968_s_at | CDC6 |
| 203971_at | SLC31A1 |
| 204025_s_at | PDCD2 |
| 204058_at | ME1 |
| 204059_s_at | ME1 |
| 204120_s_at | ADK |
| 204127_at | RFC3 |
| 204128_s_at | RFC3 |
| 204493_at | BID |
| 204532_x_at | UGT1A1 /// UGT1A10 /// UGT1A4 /// UGT1A6 /// UGT1A8 /// UGT1A9 |
| 204603_at | EXO1 |
| 204615_x_at | IDI1 |
| 204716_at | CCDC6 |
| 204748_at | PTGS2 |
| 204822_at | TTK |
| 205047_s_at | ASNS |
| 205048_s_at | PSPH |
| 205194_at | PSPH |
| 205321_at | EIF2S3 |
| 205401_at | AGPS |
| 205463_s_at | PDGFA |
| 205543_at | HSPA4L |
| 205763_s_at | DDX18 |
| 205822_s_at | HMGCS1 |
| 205909_at | POLE2 |
| 206094_x_at | UGT1A1 /// UGT1A10 /// UGT1A3 /// UGT1A4 /// UGT1A5 /// UGT1A6 /// UGT1A7 /// UGT1A8 /// UGT1A9 |
| 206205_at | MPHOSPH9 |
| 206500_s_at | MIS18BP1 |
| 206561_s_at | AKR1B10 |
| 206653_at | POLR3G |
| 206737_at | WNT11 |
| 207126_x_at | UGT1A1 /// UGT1A10 /// UGT1A4 /// UGT1A6 /// UGT1A8 /// UGT1A9 |
| 207275_s_at | ACSL1 |
| 207332_s_at | TFRC |
| 207469_s_at | PIR |
| 207891_s_at | HAUS7 /// TREX2 |
| 208079_s_at | AURKA |
| 208097_s_at | TMX1 |
| 208209_s_at | C4BPB |
| 208290_s_at | EIF5 |
| 208309_s_at | MALT1 |
| 208447_s_at | PRPS1 |
| 208510_s_at | PPARG |
| 208596_s_at | UGT1A1 /// UGT1A10 /// UGT1A3 /// UGT1A4 /// UGT1A5 /// UGT1A6 /// UGT1A7 /// UGT1A8 /// UGT1A9 |
| 208624_s_at | EIF4G1 |
| 208691_at | TFRC |
| 208708_x_at | EIF5 |
| 208839_s_at | CAND1 |
| 208840_s_at | G3BP2 |
| 208853_s_at | CANX |
| 208881_x_at | IDI1 |
| 209513_s_at | HSDL2 |
| 209520_s_at | NCBP1 |
| 209608_s_at | ACAT2 |
| 209616_s_at | CES1 /// LOC100653057 |
| 209735_at | ABCG2 |
| 209773_s_at | RRM2 |
| 209838_at | COPS2 |
| 210076_x_at | SERBP1 |
| 210544_s_at | ALDH3A2 |
| 210567_s_at | SKP2 |
| 210596_at | MAGT1 |
| 210868_s_at | ELOVL6 |
| 211015_s_at | HSPA4 |
| 211016_x_at | HSPA4 |
| 211212_s_at | ORC5 |
| 212030_at | RBM25 |
| 212097_at | CAV1 |
| 212106_at | FAF2 |
| 212107_s_at | DHX9 |
| 212190_at | SERPINE2 |
| 212220_at | PSME4 |
| 212274_at | LPIN1 |
| 212398_at | RDX |
| 212529_at | LSM12 |
| 212570_at | ENDOD1 |
| 212650_at | EHBP1 |
| 212720_at | PAPOLA |
| 213286_at | ZFR |
| 213334_x_at | HAUS7 |
| 213379_at | COQ2 |
| 213427_at | RPP40 |
| 213449_at | POP1 |
| 213470_s_at | HNRNPH1 |
| 213562_s_at | SQLE |
| 213734_at | RFC5 |
| 213880_at | LGR5 |
| 213899_at | METAP2 |
| 213913_s_at | TBC1D30 |
| 213971_s_at | SUZ12 /// SUZ12P1 |
| 214007_s_at | TWF1 |
| 214205_x_at | GLRX3 |
| 214316_x_at | CTC-425F1.4 /// OTTHUMG00000180575 |
| 214331_at | TSFM |
| 214358_at | ACACA |
| 214649_s_at | MTMR2 |
| 214697_s_at | PTBP3 |
| 214895_s_at | ADAM10 |
| 214934_at | ATP9B |
| 215125_s_at | UGT1A1 /// UGT1A10 /// UGT1A3 /// UGT1A4 /// UGT1A5 /// UGT1A6 /// UGT1A7 /// UGT1A8 /// UGT1A9 |
| 215207_x_at | NUS1 /// NUS1P3 |
| 216266_s_at | ARFGEF1 |
| 216521_s_at | BRCC3 |
| 216607_s_at | CYP51A1 /// LRRD1 |
| 216841_s_at | LOC100129518 /// SOD2 |
| 217127_at | CTH |
| 217457_s_at | RAP1GDS1 |
| 217496_s_at | IDE |
| 217834_s_at | SYNCRIP |
| 217878_s_at | CDC27 |
| 218018_at | PDXK |
| 218073_s_at | NDC1 |
| 218100_s_at | IFT57 |
| 218156_s_at | TSR1 |
| 218349_s_at | ZWILCH |
| 218392_x_at | SFXN1 |
| 218512_at | WDR12 |
| 218716_x_at | MTO1 |
| 218737_at | SBNO1 |
| 218772_x_at | TMEM38B |
| 218817_at | SPCS3 |
| 218868_at | ACTR3B |
| 218888_s_at | NETO2 |
| 219014_at | PLAC8 |
| 219201_s_at | TWSG1 |
| 219212_at | HSPA14 |
| 219307_at | PDSS2 |
| 219311_at | CEP76 |
| 219320_at | MYO19 |
| 219399_at | LIN7C |
| 219459_at | POLR3B |
| 219508_at | GCNT3 |
| 219558_at | ATP13A3 |
| 219756_s_at | POF1B |
| 219850_s_at | EHF |
| 219990_at | E2F8 |
| 220060_s_at | PARPBP |
| 220238_s_at | KLHL7 |
| 220239_at | KLHL7 |
| 220892_s_at | PSAT1 |
| 221027_s_at | PLA2G12A |
| 221059_s_at | COTL1 |
| 221305_s_at | UGT1A8 /// UGT1A9 |
| 221521_s_at | GINS2 |
| 221727_at | SUB1 |
| 221987_s_at | TSR1 |
| 222037_at | MCM4 |
| 222393_s_at | NAA50 |
| 222499_at | MRPS16 |
| 222519_s_at | IFT57 |
| 222600_s_at | UBA6 |
| 222646_s_at | ERO1L |
| 222654_at | IMPAD1 |
| 222736_s_at | TMEM38B |
| 222781_s_at | C9orf40 |
| 223062_s_at | PSAT1 |
| 223230_at | PRPF38A |
| 223308_s_at | WDR5 |
| 223320_s_at | ABCB10 |
| 223542_at | ANKRD32 |
| 223570_at | MCM10 |
| 223839_s_at | --- |
| 223879_s_at | OXR1 |
| 223888_s_at | LARS |
| 224209_s_at | GDA |
| 224311_s_at | CAB39 |
| 224352_s_at | CFL2 |
| 224461_s_at | AIFM2 |
| 224480_s_at | AGPAT9 |
| 224509_s_at | RTN4IP1 |
| 224582_s_at | NUCKS1 |
| 224634_at | GPATCH4 |
| 224744_at | IMPAD1 |
| 225161_at | GFM1 |
| 225366_at | PGM2 |
| 225686_at | SKA2 |
| 225688_s_at | PHLDB2 |
| 225736_at | FBXO22 |
| 225943_at | NLN |
| 226438_at | SNTB1 |
| 226661_at | CDCA2 |
| 226826_at | LSM11 |
| 226907_at | PPP1R14C |
| 227211_at | PHF19 |
| 227741_at | PTPLB |
| 227977_at | ZADH2 |
| 227993_at | METAP2 |
| 228050_at | UTP15 |
| 228168_at | ATP5G3 |
| 228204_at | PSMB4 |
| 228291_s_at | PLK1S1 |
| 228485_s_at | SLC44A1 |
| 228810_at | CCNYL1 |
| 228868_x_at | CDT1 |
| 228986_at | OSBPL8 |
| 229083_at | HNRNPA0 |
| 229181_s_at | HAUS2 |
| 229426_at | COX5A |
| 229644_at | PREP |
| 229676_at | MTPAP |
| 229795_at | --- |
| 229908_s_at | UNKL |
| 230250_at | PTPRB |
| 230265_at | --- |
| 230306_at | VPS26B |
| 231837_at | USP28 |
| 231995_at | CAAP1 |
| 233208_x_at | CPSF2 |
| 233665_x_at | MTO1 |
| 233819_s_at | LTN1 |
| 233827_s_at | SUPT16H |
| 234304_s_at | IPO11 /// IPO11-LRRC70 |
| 234915_s_at | DENR |
| 234992_x_at | ECT2 |
| 235134_at | LOC100996578 |
| 235244_at | CCDC58 |
| 235545_at | DEPDC1 |
| 235644_at | CCDC138 |
| 235694_at | TCFL5 |
| 235783_at | MRTO4 |
| 236957_at | CDCA2 |
| 238010_at | C1orf174 |
| 238034_at | CANX |
| 238419_at | PHLDB2 |
| 238623_at | --- |
| 238677_at | WDR36 |
| 238778_at | MPP7 |
| 239143_x_at | RNF138 |
| 240110_at | HMGCS2 |
| 241937_s_at | WDR4 |
| 242787_at | --- |
| 44065_at | C12orf52 |

## Supplementary Table 12. BZM upregulated signature.

The 682 probe sets, which were upregulated in HT-29 Pt cells treated with BZM.

| **Probe.ID** | **NAME** |
| --- | --- |
| 117_at | HSPA6 |
| 1552472_a_at | ACAP2 |
| 1552477_a_at | IRF6 |
| 1553274_a_at | SNRNP48 |
| 1553300_a_at | DGKH |
| 1553581_s_at | SREK1IP1 |
| 1554026_a_at | MYO10 |
| 1554149_at | CLDND1 |
| 1554287_at | TRIM4 |
| 1554334_a_at | DNAJA4 |
| 1554441_a_at | WAPAL |
| 1554462_a_at | DNAJB9 |
| 1554539_a_at | RHOF |
| 1555137_a_at | FGD6 |
| 1555193_a_at | ZNF277 |
| 1555274_a_at | EPT1 |
| 1555355_a_at | ETS1 |
| 1555832_s_at | KLF6 |
| 1556035_s_at | ZNF207 |
| 1557910_at | HSP90AB1 |
| 1558080_s_at | DNAJC3 |
| 1558678_s_at | MALAT1 |
| 1558924_s_at | CLIP1 |
| 1564053_a_at | YTHDF3 |
| 1567107_s_at | TPM4 |
| 1568954_s_at | C16orf72 |
| 200096_s_at | ATP6V0E1 |
| 200606_at | DSP |
| 200615_s_at | AP2B1 |
| 200632_s_at | NDRG1 |
| 200648_s_at | GLUL |
| 200664_s_at | DNAJB1 |
| 200666_s_at | DNAJB1 |
| 200730_s_at | PTP4A1 |
| 200776_s_at | BZW1 |
| 200786_at | PSMB7 |
| 200787_s_at | PEA15 |
| 200788_s_at | PEA15 |
| 200798_x_at | MCL1 |
| 200799_at | HSPA1A /// HSPA1B |
| 200800_s_at | HSPA1A /// HSPA1B |
| 200815_s_at | PAFAH1B1 |
| 200825_s_at | HYOU1 |
| 200830_at | PSMD2 |
| 200866_s_at | PSAP |
| 200868_s_at | RNF114 |
| 200871_s_at | PSAP |
| 200897_s_at | PALLD |
| 200898_s_at | MGEA5 |
| 200904_at | HLA-E |
| 200905_x_at | HLA-E |
| 200906_s_at | PALLD |
| 200907_s_at | PALLD |
| 200920_s_at | BTG1 |
| 200921_s_at | BTG1 |
| 200976_s_at | TAX1BP1 |
| 200977_s_at | TAX1BP1 |
| 201010_s_at | LOC101060503 /// TXNIP |
| 201012_at | ANXA1 |
| 201041_s_at | DUSP1 |
| 201057_s_at | GOLGB1 |
| 201131_s_at | CDH1 |
| 201170_s_at | BHLHE40 |
| 201204_s_at | RRBP1 |
| 201206_s_at | RRBP1 |
| 201207_at | TNFAIP1 |
| 201208_s_at | TNFAIP1 |
| 201252_at | PSMC4 |
| 201266_at | TXNRD1 |
| 201289_at | CYR61 |
| 201353_s_at | BAZ2A |
| 201375_s_at | PPP2CB |
| 201399_s_at | TRAM1 |
| 201428_at | CLDN4 /// LOC100996451 |
| 201464_x_at | JUN |
| 201465_s_at | JUN |
| 201466_s_at | JUN |
| 201471_s_at | SQSTM1 |
| 201531_at | ZFP36 |
| 201543_s_at | SAR1A |
| 201546_at | TRIP12 |
| 201636_at | FXR1 |
| 201637_s_at | FXR1 |
| 201711_x_at | RANBP2 |
| 201739_at | SGK1 |
| 201837_s_at | SUPT7L |
| 201856_s_at | ZFR |
| 201881_s_at | ARIH1 |
| 201898_s_at | UBE2A |
| 201975_at | CLIP1 |
| 202014_at | PPP1R15A |
| 202021_x_at | EIF1 |
| 202033_s_at | RB1CC1 |
| 202034_x_at | RB1CC1 |
| 202062_s_at | SEL1L |
| 202068_s_at | LDLR |
| 202083_s_at | SEC14L1 |
| 202087_s_at | CTSL1 |
| 202129_s_at | RIOK3 |
| 202130_at | RIOK3 |
| 202131_s_at | RIOK3 |
| 202146_at | IFRD1 |
| 202147_s_at | IFRD1 |
| 202166_s_at | PPP1R2 |
| 202195_s_at | TMED5 |
| 202211_at | ARFGAP3 |
| 202241_at | TRIB1 |
| 202267_at | LAMC2 |
| 202284_s_at | CDKN1A |
| 202286_s_at | TACSTD2 |
| 202352_s_at | PSMD12 |
| 202439_s_at | IDS |
| 202537_s_at | CHMP2B |
| 202557_at | HSPA13 |
| 202581_at | HSPA1A /// HSPA1B |
| 202636_at | RNF103 |
| 202672_s_at | ATF3 |
| 202679_at | NPC1 |
| 202684_s_at | RNMT |
| 202693_s_at | STK17A |
| 202719_s_at | TES |
| 202720_at | TES |
| 202721_s_at | GFPT1 |
| 202722_s_at | GFPT1 |
| 202730_s_at | MIR4680 /// PDCD4 |
| 202731_at | MIR4680 /// PDCD4 |
| 202733_at | P4HA2 |
| 202769_at | CCNG2 |
| 202776_at | DNTTIP2 |
| 202842_s_at | DNAJB9 |
| 202843_at | DNAJB9 |
| 202859_x_at | IL8 |
| 202880_s_at | CYTH1 |
| 202887_s_at | DDIT4 |
| 202912_at | ADM |
| 202949_s_at | FHL2 |
| 203068_at | KLHL21 |
| 203072_at | MYO1E |
| 203097_s_at | RAPGEF2 |
| 203108_at | GPRC5A |
| 203127_s_at | SPTLC2 |
| 203234_at | UPP1 |
| 203242_s_at | PDLIM5 |
| 203258_at | DRAP1 |
| 203282_at | GBE1 |
| 203320_at | SH2B3 |
| 203350_at | AP1G1 |
| 203403_s_at | RNF6 |
| 203429_s_at | SUCO |
| 203455_s_at | SAT1 |
| 203538_at | CAMLG |
| 203575_at | CSNK2A2 |
| 203588_s_at | TFDP2 |
| 203594_at | RTCA |
| 203665_at | HMOX1 |
| 203675_at | NUCB2 |
| 203725_at | GADD45A |
| 203726_s_at | LAMA3 |
| 203810_at | DNAJB4 |
| 203821_at | HBEGF |
| 203840_at | BLZF1 |
| 203853_s_at | GAB2 |
| 203925_at | GCLM |
| 203944_x_at | BTN2A1 |
| 203992_s_at | KDM6A |
| 204005_s_at | PAWR |
| 204030_s_at | IQCJ-SCHIP1 /// SCHIP1 |
| 204032_at | BCAR3 |
| 204058_at | ME1 |
| 204059_s_at | ME1 |
| 204106_at | TESK1 |
| 204137_at | GPR137B |
| 204151_x_at | AKR1C1 |
| 204157_s_at | SIK3 |
| 204180_s_at | ZBTB43 |
| 204181_s_at | ZBTB43 |
| 204182_s_at | ZBTB43 |
| 204235_s_at | GULP1 |
| 204237_at | GULP1 |
| 204285_s_at | PMAIP1 |
| 204286_s_at | PMAIP1 |
| 204346_s_at | RASSF1 |
| 204420_at | FOSL1 |
| 204435_at | NUPL1 |
| 204507_s_at | PPP3R1 |
| 204568_at | ATG14 |
| 204614_at | SERPINB2 |
| 204633_s_at | RPS6KA5 |
| 204678_s_at | KCNK1 |
| 204679_at | KCNK1 |
| 204750_s_at | DSC2 |
| 204751_x_at | DSC2 |
| 204790_at | SMAD7 |
| 204970_s_at | MAFG |
| 205009_at | TFF1 |
| 205016_at | TGFA |
| 205032_at | ITGA2 |
| 205034_at | CCNE2 |
| 205157_s_at | JUP /// KRT17 |
| 205193_at | MAFF |
| 205239_at | AREG /// AREGB |
| 205322_s_at | MTF1 |
| 205362_s_at | PFDN4 |
| 205450_at | PHKA1 |
| 205548_s_at | BTG3 |
| 205552_s_at | OAS1 |
| 205559_s_at | PCSK5 |
| 205660_at | OASL |
| 205664_at | KIN |
| 205687_at | UBFD1 |
| 205745_x_at | ADAM17 |
| 205767_at | EREG |
| 205807_s_at | TUFT1 |
| 205854_at | TULP3 |
| 205969_at | AADAC |
| 206173_x_at | GABPB1 |
| 206632_s_at | APOBEC3B |
| 206683_at | ZNF165 |
| 206788_s_at | CBFB |
| 206829_x_at | ZNF430 |
| 206884_s_at | SCEL |
| 206969_at | KRT34 /// LOC100653049 |
| 207170_s_at | LETMD1 |
| 207467_x_at | CAST |
| 207513_s_at | ZNF189 |
| 207526_s_at | IL1RL1 |
| 207528_s_at | SLC7A11 |
| 207574_s_at | GADD45B |
| 208093_s_at | NDEL1 |
| 208103_s_at | ANP32E |
| 208290_s_at | EIF5 |
| 208309_s_at | MALT1 |
| 208328_s_at | MEF2A |
| 208370_s_at | RCAN1 |
| 208420_x_at | SUPT6H |
| 208499_s_at | DNAJC3 |
| 208611_s_at | SPTAN1 |
| 208613_s_at | FLNB |
| 208653_s_at | CD164 |
| 208671_at | SERINC1 |
| 208702_x_at | APLP2 |
| 208708_x_at | EIF5 |
| 208744_x_at | HSPH1 |
| 208785_s_at | MAP1LC3B |
| 208786_s_at | MAP1LC3B |
| 208791_at | CLU |
| 208792_s_at | CLU |
| 208810_at | DNAJB6 /// TMEM135 |
| 208811_s_at | DNAJB6 /// TMEM135 |
| 208812_x_at | HLA-C |
| 208831_x_at | SUPT6H |
| 208853_s_at | CANX |
| 208868_s_at | GABARAPL1 |
| 208869_s_at | GABARAPL1 |
| 208908_s_at | CAST |
| 208925_at | CLDND1 |
| 208934_s_at | LGALS8 |
| 208936_x_at | LGALS8 |
| 208960_s_at | KLF6 |
| 208961_s_at | KLF6 |
| 208980_s_at | UBC |
| 208989_s_at | KDM2A |
| 209018_s_at | PINK1 |
| 209019_s_at | PINK1 |
| 209102_s_at | HBP1 |
| 209117_at | WBP2 |
| 209140_x_at | HLA-B |
| 209158_s_at | CYTH2 |
| 209193_at | PIM1 |
| 209222_s_at | OSBPL2 |
| 209225_x_at | TNPO1 |
| 209226_s_at | TNPO1 |
| 209260_at | SFN |
| 209270_at | LAMB3 |
| 209294_x_at | TNFRSF10B |
| 209295_at | TNFRSF10B |
| 209304_x_at | GADD45B |
| 209306_s_at | SWAP70 |
| 209345_s_at | PI4K2A |
| 209362_at | MED21 |
| 209363_s_at | MED21 |
| 209380_s_at | ABCC5 |
| 209383_at | DDIT3 |
| 209387_s_at | TM4SF1 |
| 209403_at | LOC100510707 /// LOC101060287 /// LOC101060303 /// LOC101060321 /// LOC101060351 /// LOC101060367 /// LOC101060376 /// LOC101060389 /// LOC101060403 /// LOC101060421 /// LOC101060440 /// LOC101060471 /// LOC101060489 /// LOC101060506 /// LOC101060522 /// TBC1D3 /// TBC1D3C /// TBC1D3F /// TBC1D3G /// TBC1D3H |
| 209410_s_at | GRB10 |
| 209457_at | DUSP5 |
| 209479_at | CCDC28A |
| 209653_at | KPNA4 |
| 209674_at | CRY1 |
| 209678_s_at | PRKCI |
| 209699_x_at | AKR1C2 /// LOC101060798 |
| 209744_x_at | ITCH |
| 209799_at | PRKAA1 |
| 209912_s_at | AP5Z1 /// MIR4656 |
| 209921_at | SLC7A11 |
| 209925_at | OCLN |
| 209945_s_at | GSK3B |
| 209949_at | NCF2 |
| 210018_x_at | MALT1 |
| 210041_s_at | PGM3 |
| 210087_s_at | MPZL1 |
| 210338_s_at | HSPA8 /// SNORD14C /// SNORD14D |
| 210379_s_at | TLK1 |
| 210480_s_at | MYO6 |
| 210592_s_at | SAT1 |
| 210594_x_at | MPZL1 |
| 210676_x_at | RGPD3 /// RGPD4 /// RGPD5 /// RGPD6 /// RGPD8 |
| 210732_s_at | LGALS8 |
| 210790_s_at | SAR1A |
| 210797_s_at | OASL |
| 210817_s_at | CALCOCO2 |
| 210845_s_at | PLAUR |
| 210970_s_at | IBTK |
| 210999_s_at | GRB10 |
| 211000_s_at | IL6ST |
| 211256_x_at | BTN2A1 |
| 211296_x_at | UBC |
| 211404_s_at | APLP2 |
| 211423_s_at | SC5D |
| 211458_s_at | GABARAPL1 /// GABARAPL3 |
| 211653_x_at | AKR1C2 /// LOC101060798 |
| 211707_s_at | IQCB1 |
| 211719_x_at | FN1 |
| 211764_s_at | UBE2D1 |
| 211924_s_at | PLAUR |
| 211928_at | DYNC1H1 |
| 211936_at | HSPA5 |
| 211947_s_at | PRRC2C |
| 211968_s_at | HSP90AA1 |
| 212008_at | UBXN4 |
| 212099_at | RHOB |
| 212102_s_at | KPNA6 |
| 212130_x_at | EIF1 |
| 212196_at | IL6ST |
| 212201_at | ANKLE2 |
| 212202_s_at | TMEM87A |
| 212227_x_at | EIF1 |
| 212236_x_at | JUP /// KRT17 |
| 212264_s_at | WAPAL |
| 212286_at | ANKRD12 |
| 212288_at | FNBP1 |
| 212365_at | MYO1B |
| 212400_at | FAM102A |
| 212408_at | TOR1AIP1 |
| 212409_s_at | TOR1AIP1 |
| 212428_at | KIAA0368 |
| 212436_at | TRIM33 |
| 212450_at | SECISBP2L |
| 212470_at | SPAG9 |
| 212593_s_at | MIR4680 /// PDCD4 |
| 212612_at | RCOR1 |
| 212635_at | TNPO1 |
| 212657_s_at | IL1RN |
| 212689_s_at | KDM3A |
| 212711_at | CAMSAP1 |
| 212712_at | CAMSAP1 |
| 212724_at | RND3 |
| 212756_s_at | UBR2 |
| 212780_at | SOS1 |
| 212840_at | UBXN7 |
| 213000_at | MORC3 |
| 213017_at | ABHD3 |
| 213024_at | TMF1 |
| 213026_at | ATG12 |
| 213038_at | RNF19B |
| 213076_at | ITPKC |
| 213112_s_at | SQSTM1 |
| 213115_at | ATG4A |
| 213124_at | ZNF473 |
| 213134_x_at | BTG3 |
| 213272_s_at | TMEM159 |
| 213341_at | FEM1C |
| 213349_at | TMCC1 |
| 213359_at | HNRNPD |
| 213418_at | HSPA6 |
| 213506_at | F2RL1 |
| 213530_at | RAB3GAP1 |
| 213532_at | ADAM17 |
| 213554_s_at | CDV3 |
| 213940_s_at | FNBP1 |
| 213951_s_at | PSMC3IP |
| 213988_s_at | SAT1 |
| 214150_x_at | ATP6V0E1 |
| 214155_s_at | LARP4 |
| 214168_s_at | TJP1 |
| 214356_s_at | KIAA0368 |
| 214359_s_at | HSP90AB1 |
| 214430_at | GLA |
| 214459_x_at | HLA-C |
| 214684_at | MEF2A |
| 214696_at | MIR22 /// MIR22HG |
| 214845_s_at | CALU |
| 214866_at | PLAUR |
| 214875_x_at | APLP2 |
| 214919_s_at | ANKHD1 /// ANKHD1-EIF4EBP3 /// EIF4EBP3 |
| 215034_s_at | TM4SF1 |
| 215044_s_at | STAM2 |
| 215127_s_at | RBMS1 |
| 215235_at | SPTAN1 |
| 216100_s_at | TOR1AIP1 |
| 216202_s_at | SPTLC2 |
| 216449_x_at | HSP90B1 |
| 216526_x_at | HLA-C |
| 216594_x_at | AKR1C1 |
| 216804_s_at | PDLIM5 |
| 217094_s_at | ITCH |
| 217127_at | CTH |
| 217144_at | UBBP1 /// UBBP1 /// UBBP4 /// UBBP4 |
| 217168_s_at | HERPUD1 |
| 217456_x_at | HLA-E |
| 217672_x_at | EIF1 |
| 217678_at | SLC7A11 |
| 217741_s_at | ZFAND5 |
| 217783_s_at | YPEL5 |
| 217789_at | SNX6 |
| 217911_s_at | BAG3 |
| 217924_at | C6orf106 |
| 217988_at | CCNB1IP1 |
| 217996_at | PHLDA1 |
| 218004_at | BSDC1 |
| 218013_x_at | DCTN4 |
| 218020_s_at | ZFAND3 |
| 218076_s_at | ARHGAP17 |
| 218085_at | CHMP5 |
| 218107_at | WDR26 |
| 218172_s_at | DERL1 |
| 218192_at | IP6K2 |
| 218193_s_at | GOLT1B |
| 218260_at | DDA1 |
| 218268_at | TBC1D15 |
| 218295_s_at | NUP50 |
| 218310_at | KCTD7 /// RABGEF1 |
| 218360_at | RAB22A |
| 218409_s_at | DNAJC1 |
| 218472_s_at | PELO |
| 218474_s_at | KCTD5 |
| 218562_s_at | TMEM57 |
| 218581_at | ABHD4 |
| 218603_at | HECA |
| 218614_at | KIAA1551 |
| 218645_at | ZNF277 |
| 218647_s_at | YRDC |
| 218757_s_at | UPF3B |
| 218823_s_at | KCTD9 |
| 218853_s_at | MOSPD1 |
| 218895_at | GPATCH3 |
| 218955_at | BRF2 |
| 218995_s_at | EDN1 |
| 219010_at | C1orf106 |
| 219045_at | RHOF |
| 219105_x_at | ORC6 |
| 219181_at | LIPG |
| 219237_s_at | DNAJB14 |
| 219297_at | WDR44 |
| 219311_at | CEP76 |
| 219321_at | MPP5 |
| 219356_s_at | CHMP5 |
| 219397_at | COQ10B |
| 219399_at | LIN7C |
| 219409_at | SNIP1 |
| 219474_at | C3orf52 |
| 219492_at | CHIC2 |
| 219551_at | EAF2 |
| 219681_s_at | RAB11FIP1 |
| 219774_at | CCDC93 |
| 219856_at | C1orf116 |
| 219901_at | FGD6 |
| 220018_at | CBLL1 |
| 220038_at | C8orf44-SGK3 /// SGK3 |
| 220240_s_at | TMCO3 |
| 221230_s_at | ARID4B |
| 221479_s_at | BNIP3L |
| 221596_s_at | RBM48 |
| 221664_s_at | F11R |
| 221667_s_at | HSPB8 |
| 221676_s_at | CORO1C |
| 221763_at | JMJD1C |
| 221773_at | ELK3 |
| 221778_at | JHDM1D |
| 221803_s_at | NRBF2 |
| 221841_s_at | KLF4 |
| 221918_at | CDK17 |
| 221962_s_at | UBE2H |
| 221985_at | KLHL24 |
| 222118_at | CENPN |
| 222200_s_at | BSDC1 |
| 222233_s_at | DCLRE1C |
| 222354_at | F11R |
| 222385_x_at | SEC61A1 |
| 222408_s_at | YPEL5 |
| 222420_s_at | UBE2H |
| 222458_s_at | AKIRIN1 |
| 222488_s_at | DCTN4 |
| 222493_s_at | ZFAND3 |
| 222583_s_at | NUP50 |
| 222620_s_at | DNAJC1 |
| 222621_at | DNAJC1 |
| 222699_s_at | PLEKHF2 |
| 222732_at | TRIM39 /// TRIM39-RPP21 |
| 222752_s_at | TMEM206 |
| 222767_s_at | C12orf49 |
| 222802_at | EDN1 |
| 222815_at | RLIM |
| 222850_s_at | DNAJB14 |
| 222872_x_at | NABP1 |
| 222989_s_at | UBQLN1 |
| 222991_s_at | UBQLN1 |
| 223000_s_at | F11R |
| 223027_at | SNX9 |
| 223028_s_at | SNX9 |
| 223054_at | DNAJB11 |
| 223070_at | SELK |
| 223078_s_at | TMOD3 |
| 223090_x_at | VEZT |
| 223144_s_at | AKIRIN2 |
| 223195_s_at | SESN2 |
| 223196_s_at | SESN2 |
| 223208_at | KCTD10 |
| 223242_s_at | MFSD11 |
| 223247_at | MED10 |
| 223263_s_at | FGFR1OP2 |
| 223309_x_at | PNPLA8 |
| 223310_x_at | PNPLA8 |
| 223336_s_at | RAB18 |
| 223380_s_at | LATS2 |
| 223394_at | SERTAD1 |
| 223494_at | MGEA5 |
| 223533_at | LRRC8C |
| 223551_at | PKIB |
| 223650_s_at | NRBF2 |
| 223675_s_at | VEZT |
| 223774_at | SNHG12 /// SNORA16A /// SNORA44 /// SNORA61 |
| 223940_x_at | MALAT1 |
| 223982_s_at | PNPLA8 |
| 224097_s_at | F11R |
| 224413_s_at | TM2D2 |
| 224480_s_at | AGPAT9 |
| 224502_s_at | KIAA1191 |
| 224558_s_at | MALAT1 |
| 224559_at | MALAT1 |
| 224565_at | MIR612 /// NEAT1 |
| 224566_at | MIR612 /// NEAT1 |
| 224567_x_at | MALAT1 |
| 224568_x_at | MALAT1 |
| 224606_at | KLF6 |
| 224624_at | LRRC8A |
| 224657_at | ERRFI1 |
| 224741_x_at | GAS5 /// SNORD44 /// SNORD47 /// SNORD76 /// SNORD77 /// SNORD79 /// SNORD80 /// SNORD81 |
| 224767_at | LOC100506548 /// RPL37 |
| 224790_at | ASAP1 |
| 224797_at | ARRDC3 |
| 224833_at | ETS1 |
| 224841_x_at | GAS5 /// SNORD44 /// SNORD47 /// SNORD76 /// SNORD77 /// SNORD79 /// SNORD80 /// SNORD81 |
| 224915_x_at | ZFAS1 |
| 224917_at | MIR21 /// VMP1 |
| 224933_s_at | JMJD1C |
| 224978_s_at | USP36 |
| 224984_at | NFAT5 |
| 225049_at | BLOC1S2 |
| 225061_at | DNAJA4 |
| 225088_at | FOPNL |
| 225142_at | JHDM1D |
| 225146_at | FAM219A |
| 225155_at | SNHG5 /// SNORD50A /// SNORD50B |
| 225231_at | CBL |
| 225234_at | CBL |
| 225239_at | MIR612 /// NEAT1 |
| 225265_at | RBMS1 |
| 225267_at | KPNA4 |
| 225268_at | KPNA4 |
| 225283_at | ARRDC4 |
| 225284_at | DNAJC3 |
| 225378_at | VPS37A |
| 225434_at | DEDD2 |
| 225557_at | CSRNP1 |
| 225570_at | SLC41A1 |
| 225698_at | EPB41L4A-AS1 |
| 225754_at | AP1G1 |
| 225765_at | TNPO1 |
| 225766_s_at | TNPO1 |
| 225768_at | NR1D2 |
| 225878_at | KIF1B |
| 225919_s_at | C9orf72 |
| 225950_at | SAMD8 |
| 226099_at | ELL2 |
| 226155_at | FAM160B1 |
| 226208_at | ZSWIM6 |
| 226227_x_at | ZFAS1 |
| 226275_at | MXD1 |
| 226291_at | ALS2 |
| 226307_at | CRTC2 |
| 226313_at | C10orf35 |
| 226430_at | RELL1 |
| 226541_at | FBXO30 |
| 226633_at | RAB8B |
| 226650_at | ZFAND2A |
| 226675_s_at | MALAT1 |
| 226817_at | DSC2 |
| 226835_s_at | ZFAS1 |
| 226968_at | KIF1B |
| 227020_at | YPEL2 |
| 227027_at | GFPT1 |
| 227062_at | MIR612 /// NEAT1 |
| 227152_at | KIAA1551 |
| 227224_at | RALGPS2 |
| 227278_at | TAF13 |
| 227304_at | SMCR8 |
| 227305_s_at | SMCR8 |
| 227309_at | YOD1 |
| 227314_at | ITGA2 |
| 227345_at | TNFRSF10D |
| 227371_at | BAIAP2L1 |
| 227443_at | LURAP1L |
| 227492_at | OCLN |
| 227511_at | SAMD4B |
| 227701_at | C10orf118 |
| 227904_at | AZI2 |
| 227990_at | SLU7 |
| 227991_x_at | ZBTB43 |
| 228135_at | C1orf52 |
| 228242_at | N4BP2 |
| 228248_at | RICTOR |
| 228356_at | ANKRD11 |
| 228374_at | R3HCC1L |
| 228505_s_at | TMEM170A |
| 228802_at | RBPMS2 |
| 228824_s_at | PTGR1 |
| 228846_at | MXD1 |
| 228865_at | C1orf116 |
| 228993_s_at | BBIP1 |
| 228996_at | RC3H1 |
| 229292_at | EPB41L5 |
| 229399_at | C10orf118 |
| 229566_at | LOC645638 |
| 229618_at | SNX16 |
| 229732_at | ZNF823 |
| 229865_at | FNDC3B |
| 229926_at | MIR3682 |
| 230031_at | HSPA5 |
| 230345_at | SEMA7A |
| 231718_at | SLU7 |
| 231817_at | USP53 |
| 231897_at | PTGR1 |
| 231907_at | ABL2 |
| 232612_s_at | ATG16L1 |
| 233085_s_at | NABP1 |
| 233487_s_at | LRRC8A |
| 233565_s_at | FKBP1A-SDCBP2 /// SDCBP2 |
| 234469_at | OR51B4 |
| 234978_at | SLC36A4 |
| 234989_at | MIR612 /// NEAT1 |
| 234997_x_at | OTTHUMG00000176477 /// RP11-488L18.10 |
| 235165_at | PARD6B |
| 235296_at | EIF5A2 |
| 235341_at | DNAJC3 |
| 235419_at | --- |
| 235463_s_at | CERS6 |
| 235871_at | LIPH |
| 236001_at | LINC00675 |
| 236140_at | GCLM |
| 237323_at | HKDC1 |
| 238528_at | UBR1 |
| 238542_at | ULBP2 |
| 238738_at | --- |
| 239648_at | DCUN1D3 |
| 241371_at | TNFRSF10A |
| 241599_at | LSM11 |
| 241898_at | LIPH |
| 242037_at | ASPH |
| 242722_at | LMO7 |
| 243147_x_at | --- |
| 244261_at | IFNLR1 |
| 36564_at | RNF19B |
| 36711_at | MAFF |
| 37028_at | PPP1R15A |
| 37152_at | PPARD |
| 40446_at | PHF1 |
| 57703_at | SENP5 |
| 91826_at | EPS8L1 |
| AFFX-M27830_5_at | --- |
| AFFX-M27830_M_at | --- |

## Supplementary Table 13. BZM downregulated signature.

The 888 probe sets, which were upregulated in HT-29 Pt cells treated with BZM.

| **Probe.ID** | **NAME** |
| --- | --- |
| 1552797_s_at | PROM2 |
| 1553118_at | THEM4 |
| 1553715_s_at | FAM195A |
| 1553959_a_at | B3GALT6 |
| 1553974_at | C22orf39 |
| 1553976_a_at | DPCD |
| 1553984_s_at | DTYMK |
| 1554436_a_at | REG4 |
| 1554452_a_at | HILPDA |
| 1554466_a_at | C16orf13 |
| 1554597_at | OTTHUMG00000179815 /// RP5-837J1.2 |
| 1554599_x_at | OTTHUMG00000179815 /// RP5-837J1.2 |
| 1555745_a_at | LYZ |
| 1555758_a_at | CDKN3 |
| 1555945_s_at | FAM120A |
| 1557411_s_at | SLC25A43 |
| 1558685_a_at | LOC158960 |
| 1559591_s_at | CHDH |
| 1568609_s_at | FLJ39739 /// LINC00623 /// LINC00869 /// LINC00875 /// LOC728875 |
| 200621_at | CSRP1 |
| 200644_at | MARCKSL1 |
| 200659_s_at | PHB |
| 200736_s_at | GPX1 |
| 200760_s_at | ARL6IP5 |
| 200761_s_at | ARL6IP5 |
| 200783_s_at | STMN1 |
| 200789_at | ECH1 |
| 200872_at | S100A10 |
| 200884_at | CKB |
| 200895_s_at | FKBP4 |
| 200923_at | LGALS3BP |
| 200991_s_at | SNX17 |
| 201005_at | CD9 |
| 201035_s_at | HADH |
| 201036_s_at | HADH |
| 201038_s_at | ANP32A |
| 201051_at | ANP32A |
| 201079_at | SYNGR2 |
| 201080_at | PIP4K2B |
| 201105_at | LGALS1 |
| 201125_s_at | ITGB5 |
| 201230_s_at | ARIH2 |
| 201270_x_at | NUDCD3 |
| 201284_s_at | APEH |
| 201286_at | SDC1 |
| 201301_s_at | ANXA4 |
| 201302_at | ANXA4 |
| 201328_at | ETS2 |
| 201341_at | ENC1 |
| 201350_at | FLOT2 |
| 201416_at | SOX4 |
| 201417_at | SOX4 |
| 201418_s_at | SOX4 |
| 201422_at | IFI30 /// PIK3R2 |
| 201432_at | CAT |
| 201448_at | TIA1 |
| 201487_at | CTSC |
| 201516_at | SRM |
| 201565_s_at | ID2 |
| 201566_x_at | ID2 |
| 201611_s_at | ICMT |
| 201614_s_at | RUVBL1 |
| 201628_s_at | RRAGA |
| 201656_at | ITGA6 |
| 201674_s_at | AKAP1 |
| 201675_at | AKAP1 |
| 201677_at | C3orf37 |
| 201692_at | SIGMAR1 |
| 201708_s_at | NIPSNAP1 |
| 201709_s_at | NIPSNAP1 |
| 201746_at | TP53 |
| 201816_s_at | GBAS |
| 201847_at | LIPA |
| 201853_s_at | CDC25B |
| 201896_s_at | PSRC1 |
| 201904_s_at | CTDSPL |
| 201906_s_at | CTDSPL |
| 201930_at | MCM6 |
| 201968_s_at | PGM1 |
| 201969_at | NASP |
| 202053_s_at | ALDH3A2 |
| 202054_s_at | ALDH3A2 |
| 202090_s_at | TMEM54 /// UQCR11 |
| 202096_s_at | TSPO |
| 202117_at | ARHGAP1 |
| 202139_at | AKR7A2 |
| 202144_s_at | ADSL |
| 202217_at | C21orf33 |
| 202245_at | LSS |
| 202295_s_at | CTSH |
| 202308_at | SREBF1 |
| 202418_at | YIF1A |
| 202431_s_at | MYC |
| 202472_at | MPI |
| 202486_at | AFG3L2 |
| 202489_s_at | FXYD3 |
| 202528_at | GALE |
| 202564_x_at | ARL2 |
| 202659_at | PSMB10 |
| 202737_s_at | LSM4 |
| 202741_at | PRKACB |
| 202785_at | NDUFA7 |
| 202786_at | STK39 |
| 202813_at | TARBP1 |
| 202830_s_at | SLC37A4 |
| 202831_at | GPX2 |
| 202839_s_at | NDUFB7 |
| 202846_s_at | LOC100505991 /// PIGC |
| 202903_at | LSM5 |
| 202910_s_at | CD97 |
| 202936_s_at | SOX9 |
| 202942_at | ETFB |
| 202960_s_at | MUT |
| 203028_s_at | CYBA |
| 203031_s_at | UROS |
| 203033_x_at | FH |
| 203040_s_at | HMBS |
| 203126_at | IMPA2 |
| 203148_s_at | TRIM14 |
| 203152_at | MRPL40 |
| 203186_s_at | S100A4 |
| 203188_at | B3GNT1 |
| 203219_s_at | APRT |
| 203270_at | DTYMK |
| 203274_at | F8A1 /// F8A2 /// F8A3 |
| 203276_at | LMNB1 |
| 203323_at | CAV2 |
| 203365_s_at | MMP15 |
| 203386_at | TBC1D4 |
| 203387_s_at | TBC1D4 |
| 203405_at | PSMG1 |
| 203450_at | CBY1 |
| 203453_at | SCNN1A |
| 203466_at | MPV17 |
| 203474_at | IQGAP2 |
| 203509_at | SORL1 |
| 203515_s_at | PMVK |
| 203576_at | BCAT2 |
| 203625_x_at | SKP2 |
| 203638_s_at | FGFR2 |
| 203642_s_at | COBLL1 |
| 203686_at | MPG |
| 203752_s_at | JUND |
| 203824_at | TSPAN8 |
| 203837_at | MAP3K5 |
| 203939_at | NT5E |
| 203954_x_at | CLDN3 |
| 204012_s_at | LCMT2 |
| 204014_at | DUSP4 |
| 204112_s_at | HNMT |
| 204142_at | ENOSF1 |
| 204168_at | MGST2 |
| 204169_at | IMPDH1 |
| 204256_at | ELOVL6 |
| 204259_at | MMP7 |
| 204263_s_at | CPT2 |
| 204264_at | CPT2 |
| 204295_at | SURF1 |
| 204305_at | MIPEP |
| 204326_x_at | MT1X |
| 204379_s_at | FGFR3 |
| 204386_s_at | MRP63 |
| 204394_at | SLC43A1 |
| 204404_at | SLC12A2 |
| 204484_at | PIK3C2B |
| 204521_at | FAM216A |
| 204532_x_at | UGT1A1 /// UGT1A10 /// UGT1A4 /// UGT1A6 /// UGT1A8 /// UGT1A9 |
| 204602_at | DKK1 |
| 204607_at | HMGCS2 |
| 204608_at | ASL |
| 204610_s_at | CCDC85B |
| 204702_s_at | NFE2L3 |
| 204717_s_at | SLC29A2 |
| 204745_x_at | MT1G |
| 204779_s_at | HOXB7 |
| 204798_at | MYB |
| 204805_s_at | H1FX |
| 204818_at | HSD17B2 |
| 204824_at | ENDOG |
| 204839_at | POP5 |
| 204849_at | DPH3P1 /// TCFL5 |
| 204862_s_at | NME3 |
| 204867_at | GCHFR |
| 204875_s_at | GMDS |
| 204880_at | MGMT |
| 204900_x_at | SAP30 |
| 204981_at | SLC22A18 |
| 204985_s_at | TRAPPC6A |
| 205078_at | PIGF |
| 205081_at | CRIP1 |
| 205129_at | NPM3 |
| 205311_at | DDC |
| 205313_at | HNF1B |
| 205353_s_at | PEBP1 |
| 205406_s_at | SPA17 |
| 205417_s_at | DAG1 |
| 205436_s_at | H2AFX |
| 205449_at | SAC3D1 |
| 205466_s_at | HS3ST1 |
| 205768_s_at | SLC27A2 |
| 205769_at | SLC27A2 |
| 205774_at | F12 |
| 205967_at | HIST1H4A /// HIST1H4B /// HIST1H4C /// HIST1H4D /// HIST1H4E /// HIST1H4F /// HIST1H4H /// HIST1H4I /// HIST1H4J /// HIST1H4K /// HIST1H4L /// HIST2H4A /// HIST2H4B /// HIST4H4 |
| 206066_s_at | RAD51C |
| 206094_x_at | UGT1A1 /// UGT1A10 /// UGT1A3 /// UGT1A4 /// UGT1A5 /// UGT1A6 /// UGT1A7 /// UGT1A8 /// UGT1A9 |
| 206204_at | GRB14 |
| 206239_s_at | SPINK1 |
| 206261_at | ZNF239 |
| 206399_x_at | CACNA1A |
| 206461_x_at | MT1H |
| 206463_s_at | DHRS2 |
| 206469_x_at | AKR7A3 |
| 206599_at | SLC16A5 |
| 206600_s_at | SLC16A5 |
| 206698_at | XK |
| 206734_at | JRKL |
| 206790_s_at | NDUFB1 |
| 207088_s_at | SLC25A11 |
| 207126_x_at | UGT1A1 /// UGT1A10 /// UGT1A4 /// UGT1A6 /// UGT1A8 /// UGT1A9 |
| 207335_x_at | ATP5I |
| 207414_s_at | LOC100507472 /// PCSK6 |
| 207717_s_at | PKP2 |
| 207843_x_at | CYB5A |
| 208051_s_at | PAIP1 |
| 208079_s_at | AURKA |
| 208161_s_at | ABCC3 |
| 208581_x_at | MT1X |
| 208596_s_at | UGT1A1 /// UGT1A10 /// UGT1A3 /// UGT1A4 /// UGT1A5 /// UGT1A6 /// UGT1A7 /// UGT1A8 /// UGT1A9 |
| 208651_x_at | CD24 |
| 208669_s_at | EID1 |
| 208670_s_at | EID1 |
| 208725_at | EIF2S2 |
| 208760_at | UBE2I |
| 208814_at | HSPA4 |
| 208817_at | COMT |
| 208818_s_at | COMT |
| 208833_s_at | ATXN10 |
| 208873_s_at | REEP5 |
| 208886_at | H1F0 |
| 208890_s_at | PLXNB2 |
| 208891_at | DUSP6 |
| 208892_s_at | DUSP6 |
| 208893_s_at | DUSP6 |
| 208937_s_at | ID1 |
| 208939_at | SEPHS1 |
| 208940_at | SEPHS1 |
| 208944_at | TGFBR2 |
| 208955_at | DUT |
| 208956_x_at | DUT |
| 208972_s_at | ATP5G1 |
| 208998_at | UCP2 |
| 208999_at | 42621 |
| 209001_s_at | ANAPC13 |
| 209003_at | SLC25A11 |
| 209009_at | ESD |
| 209064_x_at | PAIP1 |
| 209090_s_at | SH3GLB1 |
| 209173_at | AGR2 |
| 209201_x_at | CXCR4 |
| 209204_at | LMO4 |
| 209205_s_at | LMO4 |
| 209262_s_at | NR2F6 |
| 209263_x_at | TSPAN4 |
| 209272_at | NAB1 |
| 209366_x_at | CYB5A |
| 209377_s_at | HMGN3 |
| 209389_x_at | DBI |
| 209431_s_at | PATZ1 |
| 209449_at | LSM2 |
| 209472_at | CCBL2 |
| 209492_x_at | ATP5I |
| 209505_at | NR2F1 |
| 209529_at | PPAP2C |
| 209577_at | PCYT2 |
| 209581_at | PLA2G16 |
| 209585_s_at | MINPP1 |
| 209605_at | TST |
| 209623_at | MCCC2 |
| 209630_s_at | FBXW2 |
| 209641_s_at | ABCC3 |
| 209731_at | NTHL1 |
| 209771_x_at | CD24 |
| 209864_at | FRAT2 |
| 209892_at | FUT4 |
| 209903_s_at | ATR |
| 209932_s_at | DUT |
| 209971_x_at | AIMP2 |
| 210010_s_at | SLC25A1 |
| 210046_s_at | IDH2 |
| 210145_at | PLA2G4A |
| 210220_at | FZD2 |
| 210276_s_at | TRIOBP |
| 210377_at | ACSM3 |
| 210524_x_at | --- |
| 210544_s_at | ALDH3A2 |
| 210567_s_at | SKP2 |
| 210825_s_at | PEBP1 |
| 210868_s_at | ELOVL6 |
| 211042_x_at | MCAM |
| 211376_s_at | NSMCE4A |
| 211456_x_at | MT1HL1 |
| 211518_s_at | BMP4 |
| 211569_s_at | HADH |
| 211698_at | EID1 |
| 211730_s_at | POLR2L |
| 211941_s_at | PEBP1 |
| 211962_s_at | ZFP36L1 |
| 211984_at | CALM1 /// CALM2 /// CALM3 |
| 211985_s_at | CALM1 /// CALM2 /// CALM3 |
| 212070_at | GPR56 |
| 212096_s_at | MTUS1 |
| 212109_at | HN1L |
| 212113_at | ATXN7L3B |
| 212114_at | ATXN7L3B |
| 212149_at | EFR3A |
| 212150_at | EFR3A |
| 212155_at | RNF187 |
| 212185_x_at | MT2A |
| 212224_at | ALDH1A1 |
| 212228_s_at | COQ9 |
| 212239_at | PIK3R1 |
| 212240_s_at | PIK3R1 |
| 212256_at | GALNT10 |
| 212285_s_at | AGRN |
| 212320_at | TUBB |
| 212327_at | LIMCH1 |
| 212331_at | RBL2 |
| 212352_s_at | TMED10 |
| 212459_x_at | SUCLG2 |
| 212500_at | ADO |
| 212560_at | SORL1 |
| 212573_at | ENDOD1 |
| 212608_s_at | --- |
| 212611_at | DTX4 |
| 212614_at | ARID5B |
| 212655_at | ZCCHC14 |
| 212660_at | PHF15 |
| 212737_at | GM2A |
| 212841_s_at | PPFIBP2 |
| 212858_at | PAQR4 |
| 212859_x_at | MT1E |
| 212890_at | SLC38A10 |
| 212905_at | CSTF2T |
| 212910_at | THAP11 |
| 212993_at | NACC2 |
| 213029_at | NFIB |
| 213032_at | NFIB |
| 213041_s_at | ATP5D |
| 213107_at | TNIK |
| 213129_s_at | GCSH /// LOC101060817 |
| 213132_s_at | MCAT |
| 213133_s_at | GCSH /// LOC101060817 |
| 213147_at | HOXA10 |
| 213150_at | HOXA10 |
| 213188_s_at | MINA |
| 213189_at | MINA |
| 213227_at | PGRMC2 |
| 213237_at | KNOP1 |
| 213246_at | TMEM251 |
| 213279_at | DHRS1 |
| 213333_at | MDH2 |
| 213365_at | ERI2 |
| 213379_at | COQ2 |
| 213457_at | MFHAS1 |
| 213511_s_at | MTMR1 |
| 213581_at | PDCD2 |
| 213587_s_at | ATP6V0E2 |
| 213590_at | SLC16A5 |
| 213629_x_at | MT1F |
| 213664_at | SLC1A1 |
| 213677_s_at | PMS1 |
| 213880_at | LGR5 |
| 213889_at | PIGL |
| 213892_s_at | APRT |
| 213897_s_at | MRPL23 |
| 213975_s_at | LYZ |
| 214079_at | DHRS2 |
| 214106_s_at | GMDS |
| 214198_s_at | DGCR2 |
| 214246_x_at | MINK1 |
| 214259_s_at | AKR7A2 |
| 214484_s_at | SIGMAR1 |
| 214774_x_at | TOX3 |
| 214835_s_at | SUCLG2 |
| 214965_at | SPATA2L |
| 215030_at | GRSF1 |
| 215096_s_at | ESD |
| 215108_x_at | TOX3 |
| 215125_s_at | UGT1A1 /// UGT1A10 /// UGT1A3 /// UGT1A4 /// UGT1A5 /// UGT1A6 /// UGT1A7 /// UGT1A8 /// UGT1A9 |
| 215535_s_at | AGPAT1 |
| 215726_s_at | CYB5A |
| 215772_x_at | SUCLG2 |
| 215884_s_at | UBQLN2 |
| 215947_s_at | FAM136A |
| 216210_x_at | TRIOBP |
| 216262_s_at | TGIF2 |
| 216336_x_at | MT1P3 |
| 216379_x_at | CD24 |
| 216381_x_at | AKR7A3 |
| 216583_x_at | --- |
| 216593_s_at | LOC100505991 /// PIGC |
| 216623_x_at | TOX3 |
| 216862_s_at | CMC4 |
| 216973_s_at | HOXB7 |
| 217028_at | CXCR4 |
| 217165_x_at | MT1F |
| 217226_s_at | SFXN3 |
| 217499_x_at | OR7E37P |
| 217551_at | OR7E14P |
| 217728_at | S100A6 |
| 217752_s_at | CNDP2 |
| 217833_at | SYNCRIP |
| 217853_at | TNS3 |
| 217892_s_at | LIMA1 |
| 217900_at | IARS2 |
| 217906_at | KLHDC2 |
| 217912_at | DUS1L |
| 217948_at | FAM127B |
| 217973_at | DCXR |
| 217983_s_at | RNASET2 |
| 217984_at | RNASET2 |
| 218005_at | ZNF22 |
| 218006_s_at | ZNF22 |
| 218019_s_at | PDXK |
| 218024_at | MPC1 |
| 218034_at | FIS1 |
| 218112_at | MRPS34 |
| 218149_s_at | ZNF395 |
| 218176_at | MAGEF1 |
| 218186_at | RAB25 |
| 218190_s_at | UQCR10 |
| 218206_x_at | SCAND1 |
| 218216_x_at | ARL6IP4 |
| 218224_at | PNMA1 |
| 218265_at | SECISBP2 |
| 218270_at | MRPL24 |
| 218285_s_at | BDH2 |
| 218290_at | PLEKHJ1 |
| 218291_at | LAMTOR2 |
| 218307_at | RSAD1 |
| 218322_s_at | ACSL5 |
| 218341_at | PPCS |
| 218357_s_at | TIMM8B |
| 218377_s_at | RWDD2B |
| 218427_at | SDCCAG3 |
| 218460_at | HEATR2 |
| 218477_at | TMEM14A |
| 218491_s_at | THYN1 |
| 218507_at | HILPDA |
| 218563_at | NDUFA3 |
| 218572_at | CHMP4A |
| 218633_x_at | ABHD10 |
| 218653_at | SLC25A15 |
| 218664_at | MECR |
| 218697_at | NCKIPSD |
| 218704_at | RNF43 |
| 218741_at | CENPM |
| 218755_at | KIF20A |
| 218756_s_at | DHRS11 |
| 218765_at | SIDT2 |
| 218773_s_at | MSRB2 |
| 218788_s_at | SMYD3 |
| 218795_at | ACP6 |
| 218862_at | ASB13 |
| 218865_at | 42430 |
| 218872_at | TESC |
| 218882_s_at | WDR3 |
| 218885_s_at | GALNT12 |
| 218938_at | FBXL15 |
| 218958_at | C19orf60 |
| 218982_s_at | MRPS17 |
| 218992_at | PLGRKT |
| 219014_at | PLAC8 |
| 219041_s_at | REPIN1 |
| 219067_s_at | NSMCE4A |
| 219076_s_at | PXMP2 |
| 219109_at | SPAG16 |
| 219127_at | PRR15L |
| 219166_at | DNAAF2 |
| 219169_s_at | TFB1M |
| 219188_s_at | MACROD1 |
| 219255_x_at | IL17RB |
| 219283_at | C1GALT1C1 |
| 219289_at | HEATR3 |
| 219329_s_at | ATRAID |
| 219373_at | DPM3 |
| 219411_at | ELMO3 |
| 219491_at | LRFN4 |
| 219517_at | ELL3 |
| 219518_s_at | ELL3 |
| 219575_s_at | COG8 /// PDF |
| 219620_x_at | TOR4A |
| 219622_at | RAB20 |
| 219654_at | PTPLA |
| 219709_x_at | FAM173A |
| 219733_s_at | SLC27A5 |
| 219756_s_at | POF1B |
| 220192_x_at | SPDEF |
| 220547_s_at | FAM35A |
| 220597_s_at | ARL6IP4 |
| 220647_s_at | COA4 |
| 220865_s_at | PDSS1 |
| 220934_s_at | TMEM223 |
| 220942_x_at | FAM162A |
| 221081_s_at | DENND2D |
| 221227_x_at | COQ3 |
| 221245_s_at | FZD5 |
| 221263_s_at | SF3B5 |
| 221483_s_at | ARPP19 |
| 221543_s_at | ERLIN2 |
| 221575_at | SCLY |
| 221675_s_at | CHPT1 |
| 221688_s_at | IMP3 |
| 221692_s_at | MRPL34 |
| 221702_s_at | TM2D3 |
| 221727_at | SUB1 |
| 221786_at | C6orf120 |
| 221797_at | OXLD1 |
| 221884_at | MECOM |
| 222001_x_at | LINC00623 /// LOC728875 |
| 222013_x_at | FAM86A |
| 222280_at | LOC100506469 |
| 222403_at | MTCH2 |
| 222450_at | PMEPA1 |
| 222457_s_at | LIMA1 |
| 222468_at | KIAA0319L |
| 222499_at | MRPS16 |
| 222530_s_at | MKKS |
| 222549_at | CLDN1 |
| 222631_at | LOC285540 /// PI4K2B |
| 222662_at | PPP1R3B |
| 222685_at | HAUS6 |
| 222731_at | ZDHHC2 |
| 222750_s_at | SRD5A3 |
| 222781_s_at | C9orf40 |
| 222824_at | NUDT5 |
| 222845_x_at | TMBIM4 |
| 222853_at | FLRT3 |
| 222931_s_at | THNSL1 |
| 222986_s_at | SHISA5 |
| 223035_s_at | FARSB |
| 223051_at | SSU72 |
| 223095_at | MARVELD1 |
| 223103_at | STARD10 |
| 223112_s_at | NDUFB10 |
| 223166_x_at | RABL6 |
| 223172_s_at | MTFP1 |
| 223182_s_at | AGPAT3 |
| 223184_s_at | AGPAT3 |
| 223193_x_at | FAM162A |
| 223204_at | FAM198B |
| 223223_at | ARV1 |
| 223226_x_at | SSBP4 |
| 223227_at | BBS2 |
| 223228_at | LDOC1L |
| 223264_at | MESDC1 |
| 223272_s_at | NTPCR |
| 223275_at | PRMT6 |
| 223306_at | EBPL |
| 223312_at | PRADC1 |
| 223320_s_at | ABCB10 |
| 223349_s_at | BOK |
| 223363_at | PSMG3 |
| 223407_at | ENKD1 |
| 223411_at | MIF4GD |
| 223423_at | GPR160 |
| 223436_s_at | TRPT1 |
| 223447_at | REG4 |
| 223470_at | PIGM |
| 223485_at | HAGHL |
| 223515_s_at | COQ3 |
| 223576_at | C6orf203 |
| 223592_s_at | RNF135 |
| 223694_at | TRIM7 |
| 223892_s_at | TMBIM4 |
| 224250_s_at | SECISBP2 |
| 224301_x_at | H2AFJ |
| 224345_x_at | FAM162A |
| 224428_s_at | CDCA7 |
| 224436_s_at | NIPSNAP3A |
| 224447_s_at | MIEN1 |
| 224448_s_at | MNF1 |
| 224464_s_at | NUDT22 |
| 224478_s_at | C7orf50 |
| 224511_s_at | TXNDC17 |
| 224535_s_at | MRP63 |
| 224571_at | IRF2BP2 |
| 224576_at | ERGIC1 |
| 224581_s_at | NUCKS1 |
| 224582_s_at | NUCKS1 |
| 224584_at | TMEM230 |
| 224595_at | SLC44A1 |
| 224596_at | SLC44A1 |
| 224597_at | LINC00657 |
| 224598_at | MGAT4B |
| 224602_at | C4orf3 |
| 224604_at | C4orf3 |
| 224653_at | EIF4EBP2 |
| 224677_x_at | C11orf31 |
| 224698_at | ESYT2 |
| 224702_at | TMEM167A |
| 224711_at | YY1 |
| 224715_at | WDR34 |
| 224716_at | MIR4647 /// SLC35B2 |
| 224718_at | YY1 |
| 224749_at | ITFG3 |
| 224755_at | TM9SF3 |
| 224784_at | MLLT6 |
| 224788_at | ARF6 |
| 224818_at | SORT1 |
| 224820_at | COX20 |
| 224821_at | ABHD14B |
| 224837_at | FOXP1 |
| 224838_at | FOXP1 |
| 224847_at | CDK6 |
| 224851_at | CDK6 |
| 224867_at | MINOS1 |
| 224870_at | DANCR |
| 224876_at | C5orf24 |
| 224879_at | C9orf123 |
| 224880_at | RALA |
| 224881_at | VKORC1L1 |
| 224890_s_at | LAMTOR4 |
| 224932_at | CHCHD10 |
| 224962_at | C9orf69 |
| 224998_at | CMTM4 |
| 225009_at | CMTM4 |
| 225021_at | ZNF532 |
| 225052_at | TMEM203 |
| 225060_at | LRP11 |
| 225068_at | KLHL12 |
| 225078_at | EMP2 |
| 225108_at | AGPS |
| 225133_at | KLF3 |
| 225171_at | ARHGAP18 |
| 225173_at | ARHGAP18 |
| 225179_at | UBE2K |
| 225216_at | FAM199X |
| 225240_s_at | MSI2 |
| 225245_x_at | H2AFJ |
| 225276_at | GSPT1 |
| 225295_at | SLC39A10 |
| 225298_at | PNKD |
| 225311_at | IVD |
| 225325_at | MFSD6 |
| 225342_at | AK4 /// LOC100507855 |
| 225343_at | TMED8 |
| 225358_at | DNAJC19 |
| 225363_at | PTEN |
| 225377_at | RABL6 |
| 225380_at | PKDCC |
| 225390_s_at | KLF13 |
| 225391_at | LOC93622 |
| 225403_at | RPP25L |
| 225409_at | COA5 |
| 225410_at | COA5 |
| 225419_at | MPLKIP |
| 225420_at | GPAM |
| 225424_at | GPAM |
| 225425_s_at | MRPL41 |
| 225457_s_at | LINC00263 /// PP7080 |
| 225460_at | SEC22C |
| 225469_at | LYRM5 |
| 225478_at | MFHAS1 |
| 225532_at | CABLES1 |
| 225534_at | SMIM19 |
| 225545_at | EEF2K /// LOC101060570 |
| 225556_at | VMA21 |
| 225568_at | TMEM141 |
| 225579_at | PQLC3 |
| 225645_at | EHF |
| 225650_at | SAMD1 |
| 225657_at | NCBP2-AS2 |
| 225666_at | TMTC4 |
| 225687_at | FAM83D |
| 225688_s_at | PHLDB2 |
| 225716_at | BRI3BP |
| 225723_at | CCDC167 |
| 225733_at | B3GALT6 |
| 225739_at | RAB11FIP4 |
| 225764_at | ETV6 |
| 225777_at | SAPCD2 |
| 225823_at | C19orf70 |
| 225826_at | MMAB |
| 225835_at | SLC12A2 |
| 225845_at | ZBTB44 |
| 225861_at | FAM195A |
| 225898_at | WDR54 |
| 225902_at | PPIG |
| 225908_at | IAH1 |
| 225936_at | EID2 |
| 225967_s_at | C17orf89 |
| 225987_at | STEAP4 |
| 226016_at | CD47 |
| 226019_at | OMA1 |
| 226020_s_at | DAB1 /// OMA1 |
| 226068_at | SYK |
| 226107_at | C1GALT1 |
| 226121_at | DHRS13 |
| 226129_at | FAM83H |
| 226134_s_at | MSI2 |
| 226213_at | ERBB3 |
| 226238_at | MCEE |
| 226254_s_at | KIAA1430 |
| 226272_at | RCAN3 |
| 226301_at | SLC18B1 |
| 226336_at | LOC101060363 /// PPIA |
| 226343_at | DPP8 |
| 226350_at | CHML |
| 226360_at | ZNRF3 |
| 226374_at | CXADR |
| 226377_at | NFIC |
| 226405_s_at | ARRDC1 |
| 226410_at | CTU2 |
| 226414_s_at | ANAPC11 |
| 226420_at | MECOM |
| 226434_at | PPP1R35 |
| 226438_at | SNTB1 |
| 226456_at | RMI2 |
| 226461_at | HOXB9 |
| 226479_at | KBTBD6 |
| 226482_s_at | TSTD1 |
| 226488_at | RCCD1 |
| 226499_at | NRARP |
| 226529_at | TMEM106B |
| 226534_at | KITLG |
| 226586_at | ANKS6 |
| 226594_at | ENTPD5 |
| 226597_at | REEP6 |
| 226616_s_at | NDUFV3 |
| 226642_s_at | NUDCD2 |
| 226649_at | PANK1 |
| 226691_at | TNRC18 |
| 226707_at | NAPRT1 |
| 226710_at | C8orf82 |
| 226727_at | CISD3 |
| 226767_s_at | FAHD1 |
| 226779_at | LMBRD2 |
| 226780_s_at | C7orf55 |
| 226781_at | C7orf55 /// C7orf55-LUC7L2 |
| 226813_at | NTPCR |
| 226831_at | SLC25A46 |
| 226850_at | SUMF1 |
| 226870_at | COMTD1 |
| 226896_at | CHCHD1 |
| 226912_at | ZDHHC23 |
| 226917_s_at | ANAPC4 |
| 226943_at | C12orf73 |
| 226965_at | DENND6A |
| 226980_at | DEPDC1B |
| 227008_at | HDDC3 |
| 227042_at | YDJC |
| 227049_at | ZADH2 |
| 227052_at | --- |
| 227139_s_at | HPS3 |
| 227158_at | DTD2 |
| 227163_at | GSTO2 |
| 227186_s_at | MRPL41 |
| 227211_at | PHF19 |
| 227212_s_at | PHF19 |
| 227291_s_at | BOLA3 |
| 227342_s_at | MYEOV |
| 227356_at | --- |
| 227369_at | SERBP1 |
| 227378_x_at | C16orf13 |
| 227442_at | COX18 |
| 227466_at | FAM200B |
| 227522_at | CMBL |
| 227625_s_at | STUB1 |
| 227748_at | RBMXL1 |
| 227792_at | ITPRIPL2 |
| 227806_at | C16orf74 |
| 227856_at | C4orf32 |
| 227877_at | ANXA2R |
| 227960_s_at | FAHD1 |
| 227968_at | PDDC1 |
| 227977_at | ZADH2 |
| 228077_at | MRI1 |
| 228114_x_at | C16orf13 |
| 228129_at | SERBP1 |
| 228142_at | UQCR10 |
| 228168_at | ATP5G3 |
| 228217_s_at | PSMG4 |
| 228283_at | CMC1 |
| 228434_at | BTNL9 |
| 228488_at | TBC1D16 |
| 228535_at | RAD1 |
| 228543_at | PET117 |
| 228544_s_at | CSRP2BP /// PET117 |
| 228614_at | LINC00116 |
| 228650_at | OTTHUMG00000184130 /// RP5-935K16.1 |
| 228654_at | SPIN4 |
| 228726_at | SERPINB1 |
| 228760_at | SRSF8 |
| 228762_at | LFNG |
| 228791_at | LOC100129502 |
| 228841_at | LYRM7 |
| 228930_at | OTTHUMG00000172405 /// RP11-752G15.7 |
| 228933_at | NHS |
| 228955_at | LRP8 |
| 228956_at | UGT8 |
| 228977_at | LOC729680 |
| 228987_at | FAM49B |
| 229083_at | HNRNPA0 |
| 229099_at | C11orf83 |
| 229113_s_at | C1orf86 |
| 229126_at | TMEM19 |
| 229139_at | JPH1 |
| 229269_x_at | SSBP4 |
| 229276_at | IGSF9 |
| 229310_at | KLHL29 |
| 229332_at | HPDL |
| 229377_at | GRTP1 |
| 229394_s_at | ARHGAP35 |
| 229429_x_at | LINC00623 /// LOC728875 |
| 229498_at | MBNL3 |
| 229666_s_at | CSTF3 |
| 229742_at | C15orf61 |
| 229744_at | SSFA2 |
| 229872_s_at | LOC100132999 /// LOC100996473 /// LOC100996720 /// LOC100996740 /// LOC101060404 /// LOC101060562 /// LOC101060645 /// LOC101060698 /// LOC642441 /// LOC730256 |
| 229874_x_at | OTTHUMG00000002204 /// RP11-108M9.4 |
| 230005_at | SVIP |
| 230006_s_at | SVIP |
| 230179_at | LOC285812 |
| 230259_at | FUOM |
| 230398_at | TNS4 |
| 230399_at | --- |
| 231045_x_at | C11orf31 |
| 231059_x_at | SCAND1 |
| 231319_x_at | KIF9 |
| 231727_s_at | MIF4GD |
| 231810_at | BRI3BP |
| 231835_at | FAM213B |
| 231878_at | PAGR1 |
| 231887_s_at | PALD1 |
| 231995_at | CAAP1 |
| 232075_at | WDR61 |
| 232164_s_at | EPPK1 |
| 232165_at | EPPK1 |
| 232269_x_at | METRN |
| 232322_x_at | STARD10 |
| 232524_x_at | ANAPC4 |
| 232652_x_at | SCAND1 |
| 232899_at | LOC101060680 /// MGC2752 /// RPL23AP7 /// RPL23AP82 |
| 233049_x_at | STUB1 |
| 233167_at | SELO |
| 233341_s_at | POLR1B |
| 233571_x_at | PPDPF |
| 234107_s_at | DTD1 |
| 234672_s_at | NDC1 |
| 234998_at | RAB11A |
| 235010_at | ZBED5-AS1 |
| 235109_at | ZBED3 |
| 235134_at | LOC100996578 |
| 235256_s_at | GALM |
| 235545_at | DEPDC1 |
| 235612_at | --- |
| 235749_at | UGGT2 |
| 235931_at | METTL21A |
| 238058_at | LOC150381 |
| 238419_at | PHLDB2 |
| 238431_at | --- |
| 238571_at | HOXA13 |
| 238623_at | --- |
| 238762_at | MTHFD2L |
| 238768_at | C2orf68 |
| 238846_at | TNFRSF11A |
| 239069_s_at | --- |
| 239153_at | HOTAIR |
| 240344_x_at | LYRM7 |
| 241727_x_at | DHFRL1 |
| 242069_at | CBX5 |
| 242354_at | OTTHUMG00000172899 /// RP11-532F12.5 |
| 242888_at | PRRT3-AS1 |
| 242931_at | LONRF3 |
| 266_s_at | CD24 |
| 35820_at | GM2A |
| 36830_at | MIPEP |
| 39248_at | AQP3 |
| 39817_s_at | DNPH1 |
| 45714_at | HCFC1R1 |
| 47560_at | LPHN1 |
| 50374_at | OXLD1 |
| 51200_at | C19orf60 |
| 53968_at | INTS5 |
| 56256_at | SIDT2 |
| 64486_at | CORO1B |
| 90265_at | ADAP1 |
